# Genomic Introgression and Adaptation of Southern Seabird Species Facilitate Recent Polar Colonization

**DOI:** 10.1093/molbev/msaf053

**Published:** 2025-03-20

**Authors:** Josefina Jorquera, Lucila Morales, Elize Y X Ng, Daly Noll, Luis R Pertierra, Patricio Pliscoff, Ulises Balza, Thierry Boulinier, Amandine Gamble, Tatiana Kasinsky, Julie C McInnes, Juan Carlos Marín, Silvia Olmastroni, Pierre Pistorius, Richard A Phillips, Jacob González-Solís, Louise Emmerson, Elie Poulin, Rauri C K Bowie, Christopher P Burridge, Juliana A Vianna

**Affiliations:** Pontificia Universidad Católica de Chile, Facultad de Ciencias Biológicas, Instituto para el Desarrollo Sustentable, Santiago, Chile; Center for Bioinformatics and Integrative Biology (CBIB), Facultad de Ciencias de la Vida, Universidad Andrés Bello, Santiago, Chile; Millennium Institute Center for Genome Regulation (CGR), Santiago, Chile; Millennium Institute of Biodiversity of Antarctic and Subantarctic Ecosystems (BASE), Santiago, Chile; School of Natural Sciences, University of Tasmania, Hobart, Australia; Millennium Institute Center for Genome Regulation (CGR), Santiago, Chile; Millennium Institute of Biodiversity of Antarctic and Subantarctic Ecosystems (BASE), Santiago, Chile; Millennium Institute of Biodiversity of Antarctic and Subantarctic Ecosystems (BASE), Santiago, Chile; Department of Biogeography and Global Change, Spanish Museo Nacional de Ciencias Naturales (MNCN-CSIC), Madrid, Spain; Millennium Institute of Biodiversity of Antarctic and Subantarctic Ecosystems (BASE), Santiago, Chile; Centro de Estudios Territoriales, Universidad de Los Andes, Santiago, Chile; Centro Austral de Investigaciones Científicas (CADIC-CONICET), Ushuaia, Argentina; Centre d’Ecologie Fonctionnelle et Evolutive (CEFE), UMR 5175, CNRS, Université Montpellier, EPHE, IRD, Montpellier, France; Department of Public and Ecosystem Health, Cornell University, Ithaca, NY, USA; Centro para el Estudio de Sistemas Marinos, CONICET, Puerto Madryn, Chubut, Argentina; Institute for Marine and Antarctic Studies, University of Tasmania, Hobart, Australia; Science Branch, Australian Antarctic Division, Kingston, Tasmania, Australia; Universidad del Bio-Bío, Departamento de Ciencias Básicas, Facultad de Ciencias, Laboratorio de Genómica y Biodiversidad, Chillán, Chile; Department of Physical, Earth and Environmental Sciences, University of Siena, Siena, Italy; Museo Nazionale dell’Antartide “F. Ippolito,”, Siena, Italy; Marine Apex Predator Research Unit (MAPRU), Institute for Coastal and Marine Research and Department of Zoology, Nelson Mandela University, Port Elizabeth, South Africa; British Antarctic Survey, Natural Environment Research Council, Cambridge, UK; Institut de Recerca de la Biodiversitat (IRBio), Dept. Biologia Evolutiva, Ecologia i Ciències Ambientals, Universitat de Barcelona, Barcelona, Spain; Science Branch, Australian Antarctic Division, Kingston, Tasmania, Australia; Millennium Institute of Biodiversity of Antarctic and Subantarctic Ecosystems (BASE), Santiago, Chile; Laboratorio de Ecología Molecular (LEM), Departamento de Ciencias Ecológicas, Facultad de Ciencias, Universidad de Chile, Santiago, Chile; Museum of Vertebrate Zoology & Department of Integrative Biology, University of California, Berkeley, CA, USA; School of Natural Sciences, University of Tasmania, Hobart, Australia; Pontificia Universidad Católica de Chile, Facultad de Ciencias Biológicas, Instituto para el Desarrollo Sustentable, Santiago, Chile; Millennium Institute Center for Genome Regulation (CGR), Santiago, Chile; Millennium Institute of Biodiversity of Antarctic and Subantarctic Ecosystems (BASE), Santiago, Chile; Millennium Nucleus of Patagonian Limit of Life (LiLi), Santiago, Chile

**Keywords:** comparative population genomics, speciation, positive selection, future niche projections, polar regions

## Abstract

Genomic adaptation and introgression can occur during the speciation process, enabling species to diverge in their frequencies of adaptive alleles or acquire new alleles that may promote adaptation to environmental changes. There is limited information on introgression in organisms from extreme environments and their responses to climate change. To address these questions, we focused on the 3 southern skua species, selected for their widespread distribution across the Southern Hemisphere and their complex history of speciation and introgression events. Our genomic data reveal that these skuas underwent diversification around the Penultimate Glacial Period, followed by subsequent demographic expansion. We identified a geographic region of introgression among species that followed a directional pattern sourced from the Antarctic continent, South America, and east to west in subantarctic islands, all converging towards the Antarctic Peninsula. The 3 skua species and admixed individuals exhibited a unique pattern of putative genes under selection, allowing adaptation to extreme conditions. Individuals with a higher proportion of Brown Skua ancestry showed signs of selection on genes related to reproductive isolation, while admixed individuals with a higher proportion of South Polar Skua ancestry displayed patterns resembling those of the South Polar Skua. Introgression may be a key mechanism of adaptation for many species that may help buffer against the ongoing climate change.

## Graphical Abstract

**Graphical Abstract:**
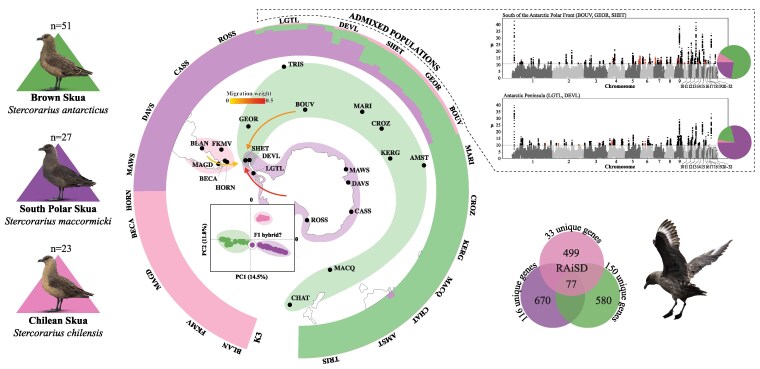

## Introduction

During speciation, ecological and genetic divergence in the presence of gene flow becomes likely when reproductive isolation is driven by divergent selection toward new habitats (Egan et al. 2015). In this context, hybridization between lineages can play a crucial role in the speciation process, leading to 2 possible outcomes: homogenization of the gene pool or production of new combinations of genetic variants from different lineages (Singhal et al. 2021). Hybridization is generally less frequent in groups of species that develop reproductive barriers more rapidly (Meier et al. 2017). However, the biogeographic and environmental context of speciation is also important, as species originating in close proximity to one another have more opportunities for hybridization (Hamlin et al. 2020). Therefore, understanding patterns in which hybridization across different geographic regions, habitats, or lineages can shed light on the dynamics of speciation.

The role of introgression in evolution remains controversial due to the challenges involved in accurately identifying introgressed genomic regions and distinguishing them from incomplete lineage sorting (ILS; Durand et al. 2011; Solís-Lemus and Ané 2016; Vianna et al. 2020; Choi et al. 2021). The retention of ancestral polymorphisms between species (ILS) and introgression are common phenomena observed in recent speciation events (Edelman et al. 2019). For this reason, it is very important to distinguish between both and to determine the degree to which hybridization shapes the genomes of species. Since the extent of the introgressed region can change over time, understanding the processes through which genomes resist ongoing introgression or stabilize after hybridization events can help us understand speciation on different temporal scales. Different admixture events also allow us to ask questions about the early and late stages of speciation, as well as the stabilization of the genome after hybridization. Episodes of introgression among species allow lineages to gain adaptive variation faster than would be expected through mutation (Ding et al. 2014). Therefore, the introduction of novel advantageous alleles followed by generations of recombination and natural selection should allow us to identify the location of those alleles and monitor their frequency in the parental or hybrid populations (Schumer et al. 2018). Therefore, natural hybrid zones make it possible to identify the mechanisms that facilitate and inhibit speciation.

Genome-wide data can offer valuable insights into adaptation and introgression. To achieve a comprehensive spatial and temporal understanding of introgression, it is essential to sample broadly across species' ranges (Blanco-Pastor 2022). However, introgression studies often face limitations due to insufficient geographic representation of the taxa and evaluate only single representatives of a species (e.g. Mikkelsen and Irwin 2021). While these studies can offer useful estimates of introgression, their results may be misleading if introgression varies through space, if there are taxonomic uncertainties regarding some populations, if areas of introgression are not well-documented, or if admixture varies among one or more taxa. Therefore, accurately quantifying the magnitude and directionality of introgression between species using genomic data can depend heavily on geographic sampling.

An appealing candidate system for testing adaptations via introgression is provided by the skuas (*Stercorarius* spp.), a group of seabirds that are generalist predators and scavengers, distributed in both hemispheres. At present, 3 species have been described for the Southern Hemisphere with somewhat distinct breeding ranges: the Chilean Skua (*Stercorarius chilensis*) breeding on South America, the South Polar Skua (*S. maccormicki*) breeding on Antarctica, and the Brown Skua (*S. antarcticus*) breeding mostly on subantarctic islands. Additionally, 3 subspecies are delimited for the Brown Skua: the Subantarctic skua (*S. a. lonnbergi*), Tristan Skua (*S. a. hamiltoni*), and Falklands Skua (*S. a. antarcticus*). Skua classification at the level of species and subspecies have been under debate since the first studies of mtDNA and nuclear markers, which were unable to identify a resolved phylogeny even though samples were collected at multiple locations (Cohen et al. 1997; Braun and Brumfield 1998; Andersson 1999; Ritz et al. 2006; Chu et al. 2009; Mikkelsen and Weir 2023; Mota et al. 2023). As such, a single taxonomic group has been suggested (Ritz et al. 2008). However, failure to confidently resolve phylogenetic and taxonomic relationships may reflect ILS, introgression among species, or the limitations of using few markers. ILS and introgression result in incongruences between gene and species/population trees, which can be better estimated and distinguished by different methods using genomic data (Durand et al. 2011; Malinsky et al. 2021).

Recent introgression among Southern Hemisphere skuas is supported on observations in the field of apparent hybrid backcrossing with parental species to produce fertile offspring. Male South Polar Skuas and female Brown Skuas have been observed to interbreed (Trivelpiece et al. 1980; Ritz et al. 2006), but there is no genetic evidence of the directionality of gene flow between these species. These species breed in sympatry over an area of >500 km across the Antarctic Peninsula to as far north as the South Orkney Islands and are suspected to have diverged recently (Hemmings 1984; Ritz et al. 2006). There are also isolated records of mixed pairs of Chilean and South Polar Skuas at King George Island, Antarctica (Reinhardt et al. 1997) and rare hybridization between Chilean and Falkland Skuas on the coast of Argentina (Devillers 1977; Gandini and Frere 1998). The potential for historical introgression among these taxa is also suggested by their breeding distributions both north and south of the Antarctic Polar Front (APF) and Subtropical Front (STF). The APF and STF separate Antarctic, subantarctic, and sub-tropical waters and represent biogeographical barriers that promote the isolation, speciation, and local adaptation of numerous marine taxa (Griffiths et al. 2009; Frugone et al. 2018). During the Pleistocene climatic oscillations, the sea-ice extent was highly variable and the geographic position of the fronts changed, associated with alternating population bottlenecks and periods of expansion (Fraser et al. 2012). There is evidence that this resulted in secondary contact and hybridization among seabird species, including giant petrels (Techow et al. 2010), prions (Masello et al. 2019), and skuas (Ritz et al. 2006). This spatial and temporal habitat heterogeneity offers a special opportunity to investigate the extent of molecular adaptation to climatic extremes across and within species and, therefore, introgressive adaptation. Finally, while hybridization and introgression of skua species has been documented genetically (Mikkelsen and Weir 2023), this has never been extensively evaluated for Southern Hemisphere skuas using genomic data.

This study aims to assess the significance of ILS, introgression, and adaptation in the speciation and evolutionary history of Southern Hemisphere skuas across their entire distribution. We examined the spatial extent of introgressive hybridization, as well as the pattern and directionality of introgression among taxa. To achieve this, we first clarified the evolutionary history of skuas, including the timing of speciation and secondary contact, and delimited taxonomic groups, their geographic distribution, and the genomic signatures of selection. By reconstructing demographic history, we revealed the timing of diversification and the impact of past climate oscillations on effective population size, which is often associated with the degree and effects of introgression. Finally, we applied species distribution models under different climatic change scenarios to predict possible range expansions, contractions, overlaps, and impacts in the area of introgression, and the potential consequences for differentiation among the extant skua taxa.

## Results

**Fig. 1.**
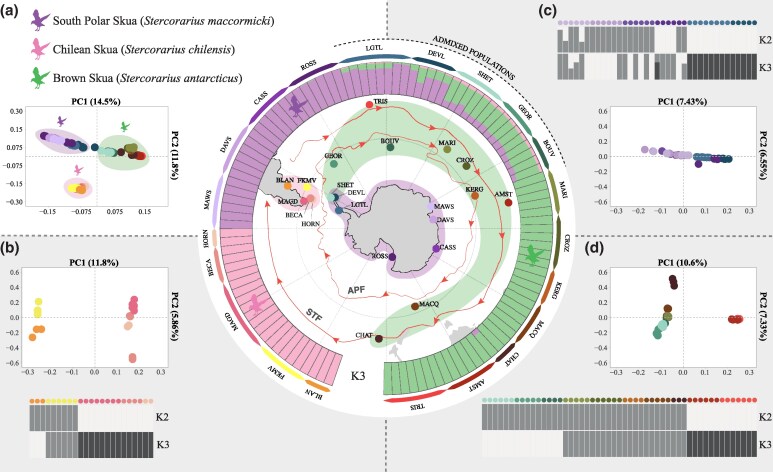
Geographic distribution, population structure, and genetic diversity of the southern skua complex. The colored dots in the map indicate the sampling sites included in this study. The red arrows represent the path of the Subtropical Front (STF) as described by Orsi et al. (1995) and the Antarctic Polar Front (AFP) as described by Civel-Mazens et al. (2021). The basemap was constructed using the Norwegian Polar Institute's Quantarctica package (Matsuoka et al. 2021). Locations for Chilean Skuas: Magdalena Island (MAGD, *n* = 8), Becasses Island (BECA, *n* = 4), Hornos Island (HORN, *n* = 2), Blancas Islands (BLAN, *n* = 3), Falkland/Malvinas Islands (FKMV, *n* = 6); Brown Skuas: South Shetland Islands (SHET, *n* = 6), South Georgia Island (GEOR, *n* = 5), Bouvet Island (BOUV, *n* = 4), Marion Island (MARI, *n* = 5), Crozet Islands (CROZ, *n* = 6), Kerguelen Islands (KERG, *n* = 4), Macquarie Island (MACQ, *n* = 5), Chatham Islands (CHAT, *n* = 3), Amsterdam Island (AMST, *n* = 6), Tristan da Cunha archipelago (TRIS, *n* = 7); South Polar Skuas: Mawson Station (MAWS, *n* = 6), Davis Station (DAVS, *n* = 6), Casey Station (CASS, *n* = 6), Ross Sea (ROSS, *n* = 6), Devil Island (DEVL, *n* = 5), Lagotellerie Island (LGTL, *n* = 8). Population structure inferred from ADMIXTURE analysis among all individuals (Central area of the Figure), supporting 3 main genetic groups with the lowest cross-validation error at *K* = 3. The semicircular color-coded bars around the ADMIXTURE indicate each of the 21 sampling locations included in the analysis. PCA among all individuals a) with the first 2 principal components (PC1 and PC2) explaining 14.5% and 11.8% of the variation, respectively. PCA and ADMIXTURE for Chilean Skua b), South Polar Skua c), and Brown Skua d). *Stercorarius* icon by Liam Quinn (photo) and Albertonykus (silhouette).

We analyzed whole-genome sequences of 111 skua individuals sampled from 21 geographic locations. Our spatial extent of sampling represents the entirety of the species' distribution ranges in the Southern Hemisphere (Fig. 1 and supplementary tables S1 and S2, Supplementary Material online). The average sequencing coverage was 15.1× and the total number of reads analyzed was ∼13 billion (supplementary table S3, Supplementary Material online).

### Population Genetic Structure and Differentiation

Following quality filtering and linkage disequilibrium (LD) pruning, approximately 992,654 high-quality biallelic single nucleotide polymorphisms (SNPs; supplementary table S4, Supplementary Material online) were used to explore genetic differentiation among the Southern Hemisphere skuas. Principal component analysis (PCA) revealed 3 distinct genomic groups supporting the currently described species boundaries between the South Polar Skua, Brown Skua, and Chilean Skua, with 14.5% and 11.8% of the variance explained on the first 2 axes (Fig. 1a, supplementary fig. S1, Supplementary Material online). Results of the interspecific ADMIXTURE analysis agreed with those of the PCA, as the optimal number of ancestral populations identified with the lowest cross-validation error was *K* = 3 (Fig. 1, supplementary fig. S2, Supplementary Material online). However, almost all individuals of the South Polar Skua from populations at the Antarctic Peninsula (Devil Island and Lagotellerie Island) and every Brown Skua from populations south of the Antarctic Polar Front (APF; South Shetland Islands, South Georgia Island, Bouvet Island), shared ancestry components from the 3 genetic clusters (Fig. 1, supplementary table S5, Supplementary Material online). We refer to these 5 localities as “admixed populations”, although it is important to note that the contribution of South Polar Skua and Brown Skua ancestry is different, with the latter predominant in the populations south of the APF (SAPF) and the former in those at the Antarctic Peninsula (ANTP; Fig. 1). Of note, we identified an admixed individual from Devil Island (DEVL2) that showed a nearly equal contribution of South Polar and Brown Skua ancestry and an intermediate placement between the 2 species groups on the PCA, a pattern characteristic of F1 hybrids (Fig. 1a). The most striking result observed in both interspecific grouping analyses is that the Falkland Skua populations formed a unique group with the Chilean Skua populations, contrary to the pattern expected for a supposed subspecies of the Brown Skua. Therefore, Falkland Skuas will now be conspecific with Chilean Skua for all results described in this section. Furthermore, we found that the skuas from Tristan da Cunha archipelago, currently classified as a subspecies of the Brown Skua, formed a distinct cluster with individuals from Amsterdam Island (supplementary fig. S2, Supplementary Material online; *K* = 4), suggesting they should be considered part of the Tristan Skua subspecies distribution.

The PCA and ADMIXTURE analysis performed within each of the 3 main groups separately revealed finer population structure (Fig. 1). PCA differentiated the Chilean and Falkland populations along the first principal component, which explained 11.8% of the variance (Fig. 1b). Sub-structure was observed within the South Polar Skua, with the populations from the Antarctic Peninsula and elsewhere on the continent (Mawson Station, Davis Station, Casey Station, Ross Sea) forming distinct genetic groups (Fig. 1c). Brown Skuas were split into 3 geographic subgroups (Fig. 1d). Specifically, the first principal component distinguished the Amsterdam and Tristan da Cunha populations, explaining 10.6% of the variance. The second principal component accounted for 7.3% of the variance and separated the Chatham Islands population. These PCA inferences based on subsets of the data are also supported by ADMIXTURE results (supplementary figs. S3 to S5, Supplementary Material online).

The inferred Maximum-likelihood (ML) phylogeny supports the Chilean Skuas as sister taxa to the Brown and South Polar Skuas (supplementary fig. S6, Supplementary Material online). The ML phylogeny places the Amsterdam and Tristan da Cunha individuals within the same clade, and the Falkland Skua clustered as a sister clade to the other Chilean Skua individuals. For the Brown Skua clade, the branching pattern is laddered from south to north of the APF, and then north of the STF. In contrast, the branching of South Polar Skuas occurs from the Antarctic Peninsula to Continental Antarctica.

The phylogeny and population structure of all skua samples based on analyses of nuclear SNPs revealed 7 phylogeographic groups (supplementary fig. S6, Supplementary Material online): Southern Chile (CHIL; Magdalena Island, Becasses Island, Hornos Island); Southwestern Atlantic (FKLD; Blancas Islands, Falkland/Malvinas Islands); Antarctic Peninsula (ANTP; Devil Island, Lagotellerie Island); Continental Antarctica (ANTC; Mawson Station, Davis Station, Casey Station, Ross Sea); South of the APF (SAPF; South Shetland Islands, South Georgia Island, Bouvet Island); North of the APF (NAPF; Marion Island, Crozet Islands, Kerguelen Islands, Macquarie Island, Chatham Islands); North of the STF (NSTF; Tristan da Cunha archipelago, Amsterdam Island). Although located north of the STF, the Chatham Islands were not included in the NSTF phylogeographic group but rather with the NAPF group, as they grouped more closely with these samples in the PCA.

**Fig. 2.**
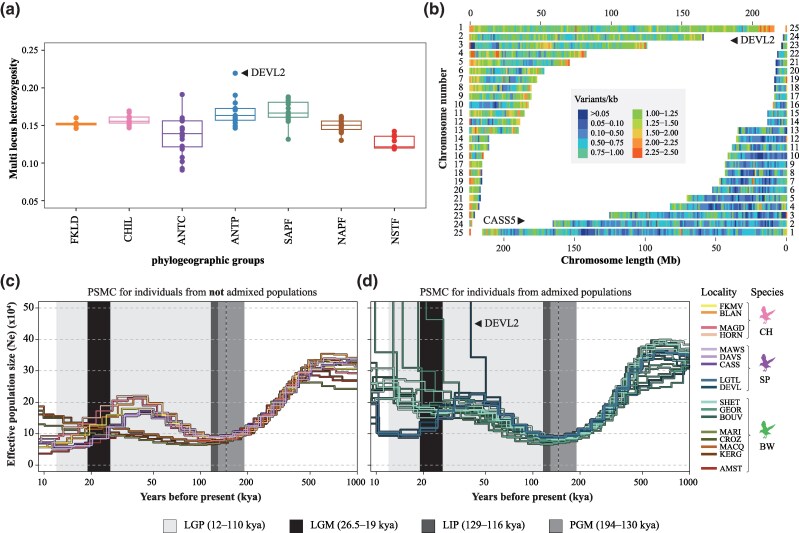
Genetic diversity and demographic history of the southern skua complex. a) Mean heterozygosity per geographic groups of the 3 species. b) Density of SNPs (per 1 Kb window) across chromosome-length scaffolds of an F1 hybrid individual (DEVL2) above and an individual of the same species without admixture (ROSS1) below. c) Estimates of the past effective population size (Ne) by PSMC for individuals of similar sequence coverage (∼14 to 15×) of each location with non-admixed populations. d) PSMC including representant individuals from the admixed populations as revealed by the intraspecific ADMIXTURE. Vertical shaded areas represent key glaciation events: penultimate glacial maximum (PGM, 194–130 ka), last interglacial period (LIG, 129–116 ka), last glacial period (LGP, 12–110 ka), last glacial maximum (LGM, 26.5–19 ka). The plots were scaled using a mutation rate (μ) of 5.9×10^−9^ mutations per site per generation and a generation time of 6 yr. CHIL: Southern Chile (MAGD, BECA), FKLD: Southwest Atlantic shelves (FKMV), ANTP: Antarctic Peninsula (DEVL, LGTL), ANTC: Continental Antarctica (MAWS, CASS, DAVS, ROSS), SAPF: South of the Antarctic Polar Front (BOUV, SHET, GEOR), NAPF: North of the Antarctic Polar Front (KERG, CROZ, MARI, MACQ), NSTF: North of the Subtropical Front (AMST, TRIS).

Pairwise F_ST_ values were significant for most localities and phylogeographic groups (supplementary figs S7 and S8, Supplementary Material online). The highest *F_ST_* values were between Brown Skuas from Tristan da Cunha archipelago and Chilean Skuas from the Falkland Islands (*F_ST_* = 0.254). The lowest *F_ST_* values, close to zero, are between South Polar Skuas colonies (*F_ST_* = 0.0004). Brown Skua individuals from populations north of the Subtropical Front (NSTF), including the Tristan da Cunha archipelago and Amsterdam Island, showed the lowest heterozygosity (Fig. 2a, supplementary figs S9 and S10, Supplementary Material online). In contrast, the highest heterozygosity was observed in individuals from populations located within the admixture zone, those located south of the APF (SAPF), and the Antarctic Peninsula (ANTP). The putative F1 hybrid from Devil Island showed the highest heterozygosity among all samples (supplementary fig. S10, Supplementary Material online). The individuals from the admixture zone demonstrates a higher SNP density along the genome when compared to individuals of the same species from a non-admixed population (Fig. 2b, supplementary fig. S11, Supplementary Material online). Tajima's D was positive for the Southwestern Atlantic (FKLD) and Southern Chile (CHIL) groups, suggesting a population contraction. In contrast, negative Tajima's D values were observed for the South Polar and Brown Skua, with the groups from the admixture zone (ANTP and NAPF) showing a stronger signal of population expansion (supplementary fig. S12, Supplementary Material online).

### Footprints of Hybridization on Demographic History

Overall, divergent patterns of population dynamics were observed following the Penultimate Glacial Maximum (PGM) that could be associated with the time of speciation (∼200 kya; Fig. 2c, supplementary figs S13 and S14, Supplementary Material online). A first decrease at the time of the PGM was observed in all populations, leading to a Ne minimum between 130 and 194 kya (Fig. 2c, d). All populations increased in Ne until ∼50 kya, but the magnitude of the increase was greater in the Chilean and South Polar populations than in the Brown Skua populations. In the last 50 kya, the Chilean and South Polar Skua populations decreased, reaching a second Ne minimum at the end of the Last Glacial Maximum (LGM), whereas Brown Skua populations continued to increase during this period, except at Amsterdam Island. In particular, the Amsterdam Island population showed the most significant drop in Ne since the LGM among all populations analyzed (Fig. 2c). The magnitude of the Ne expansion right after the Last Interglacial Period (LIG) of the South Polar and Brown Skua admixed populations was higher than their respective non-admixed populations (Fig. 2d). This Ne increase is more evident in the Brown Skua admixed populations (South Shetland Islands, South Georgia Island, Bouvet Island) and even more distinguishable in the F1 hybrid from Devil Island (DEVL2).

### Patterns of Gene Flow and Introgression

**Fig. 3.**
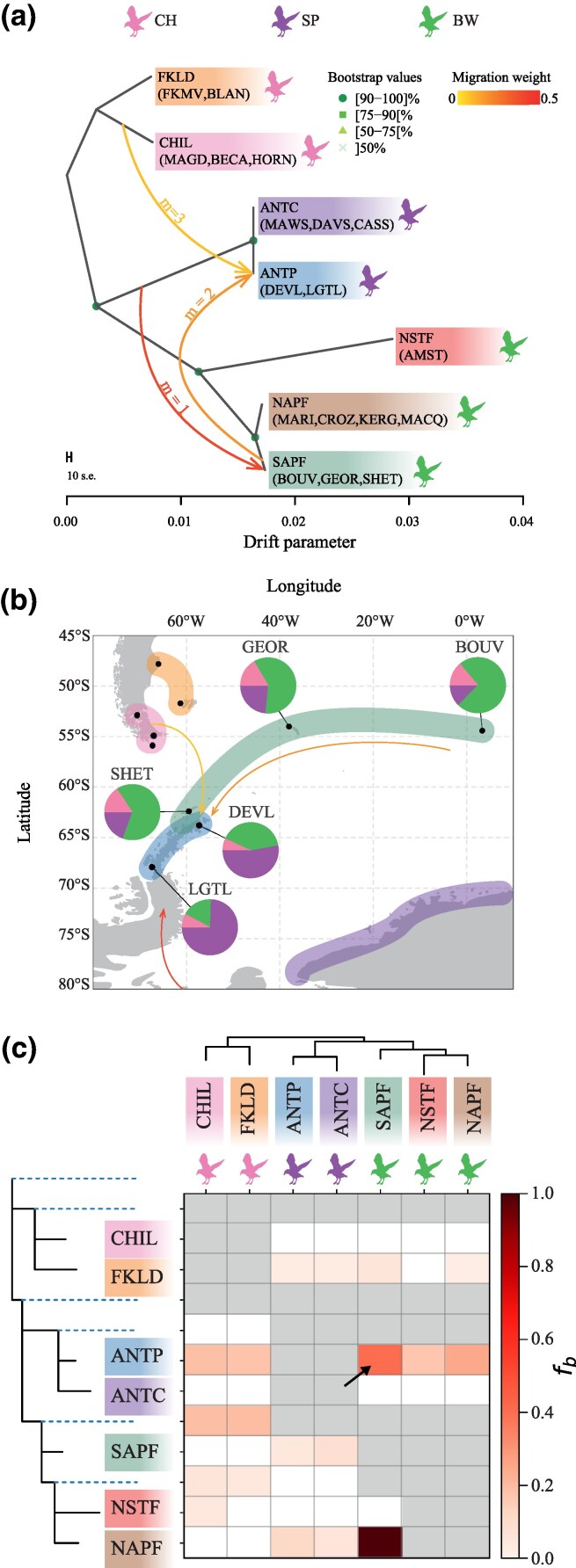
Footprints of introgression inferred across the southern skua complex. Population names are colored according to the 7 phylogeographic groups defined based on the topology of the ML tree a) Unrooted ML tree inferred by TreeMix; the arrows denote 3 migration events from the origin to the recipient locality. Migration arrows are colored according to their weight. b) Southern South America, Subantarctica, and Antarctic Peninsula map showing the admixed populations: 2 from the Antarctic Peninsula (LGTL, DEVL) with a high percentage of South Polar Skua and 3 in the maritime Antarctic (BOUV, SHET, GEOR) with a higher percentage of Brown Skua ancestry. c) Genomic introgression results for Southern Hemisphere skua using the f-branch method. Darker colors in the heat map represent increasing evidence of specific gene flow events between lineages, and gray data points in the matrix correspond to tests that are not applicable to the provided phylogeny. Dashed lines in the phylogeny represent the ancestral lineage. The *D* statistic and f4-ratio reflect evidence of excess allele exchange between P3 and P2 for each trio.

The ADMIXTURE analysis showed a total of 31 individuals with any degree of admixture between species, considered as signature of past introgression, falling into 2 patterns: (i) 2 sites on the Antarctic Peninsula with a higher percentage of South Polar Skua (70% to 96%) than Brown Skua (4% to 46%), and an always small or absent signature of Chilean Skua (0% to 6%); (ii) 3 sites from south of the APF with a higher percentage of Brown Skua (56% to 75%), a lower percentage of South Polar Skua (18% to 34%), and a small percentage of Chilean Skua (8% to 10%; Figs. 1 and  3b, supplementary table S5, Supplementary Material online). DEVL2 showed 48%, 46%, and 6% of South Polar, Brown, and Chilean Skuas, respectively. Three other individuals from other locations showed a low degree of ADMIXTURE.

To assess evolutionary divergence and test whether the phylogeny followed a simple branching tree or a network with gene flow and introgression, we reconstructed a phylogeny using TreeMix. The results infer that gene flow events occurred in the past with a north-south and east-west directionality (Fig. 3a, supplementary figs. S15 and S16, Supplementary Material online). TreeMix analysis indicates the direction of gene flow, with the following migration patterns arranged by the weight of more recent gene flow events (*m* = 3). Gene flow primarily occurred from South Polar Skuas toward the South Shetlands Islands, South Georgia, and Bouvet Island (*m* = 1, SAPF). Subsequently, gene flow moves from this region towards South Polar Skuas from Antarctic Peninsula (*m* = 2, ANTP). Additionally, the latter location also receives gene flow from Chilean Skuas (*m* = 3).

Finally, to support the evidence for introgression among skua species, we utilized an approach based on *D*-statistics. This method estimates D and f4 statistics across all possible combinations of trios in skuas and then performs an *f*-branch test to assign gene flow to specific internal branches (Fig. 3c). The matrix displays the inferred introgression proportions based on gene tree counts for introgressed species pairs, mapped to internal branches using the f-branch (fb) method. The expanded tree at the top of each matrix shows both the terminal and ancestral branches. The values in the matrix thus refer to excess allele sharing between branch (b) identified on the expanded tree on the y-axis (relative to its sister branch) and species P3 identified on the x-axis. Our results reject the null hypothesis of no gene flow between P3 and either P1 or P2, implying that introgression, rather than incomplete lineage sorting, is a more plausible explanation for the observed shared polymorphism. The *f*-branch test identified significant genetic exchange, primarily within Brown Skuas (SAPF and NAPF; Fig. 3c). A secondary event was observed between Brown Skuas populations south of the APF (SAPF) and South Polar Skuas at the Antarctic Peninsula (ANTP; Fig. 3c). Out of 35 trios tested, 5 showed a significant excess of shared alleles, with a mean excess of shared alleles greater than 0.05 (5%; supplementary fig. S17, tables S6 and S7, Supplementary Material online).

### Genomic Signatures of Adaptation

To identify genomic regions with positive selection signals, we analyzed the species using complementary methodologies: Raised Accuracy in Sweep Detection (RAiSD) software (Alachiotis and Pavlidis 2018) and XP-nSL (novel haplotype-based scan; Zachary et al. 2021). The μ statistic from RAiSD results is a composite test that evaluates genomic regions by scoring changes in the site frequency spectrum (SFS), LD, and genetic diversity across a chromosome, using a novel SNP-vector approach to optimize computational efficiency. In contrast, the XP-nSL analysis identified genes associated with regions containing extended haplotypes that were differentially selected compared to a reference population. Both approaches allow for the examination of recent and long-term signals of selection for admixed and non-admixed populations by detecting genome-wide selective sweeps that produce regions of reduced genetic diversity in the vicinity of an adaptive mutation.

**Fig. 4.**
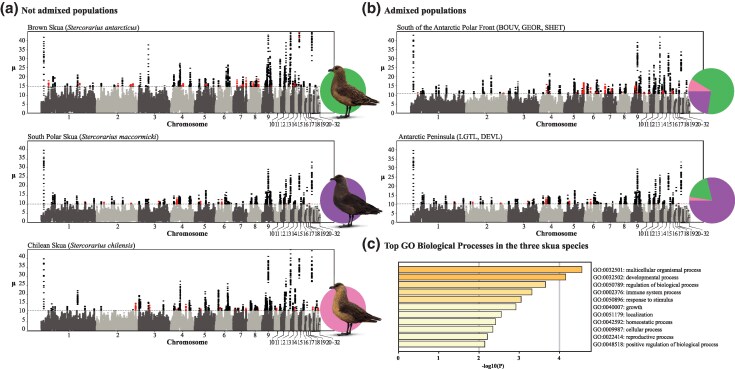
Genomic signatures of adaptation. a) Plots of genome-wide selection scans with RAiSD µ results for SNPs of the Brown, South Polar and Chilean Skuas, and b) plots for admixed individuals. The μ evaluates genomic regions by scoring changes in the site frequency spectrum (SFS), linkage disequilibrium (LD) levels, and genetic diversity across a chromosome. Dashed lines are the threshold used for µ-values >99.5% across all chromosomes. Default overlapping window size of 50 kb was used. Black dots represent SNPs shared across 2 or more skua species and red dots are private SNPs to the denoted species. c) Metascape bar graph showing the top-level GO biological processes of the common genes under selection among the 3 skua species, considering non-admixed populations. Representative images of each taxon by Rodrigo Verdugo.

Using RAiSD to analyze regions with positive selection signals, we identified a total of 670 genes in South Polar Skuas, 580 genes in Brown Skuas, and 499 genes in Chilean Skuas (supplementary table S8, Supplementary Material online). Among these genes, 77 are shared across all 3 species, and 116, 150, and 33 were unique for South Polar, Brown, and Chilean Skuas, respectively (Fig. 4a and supplementary tables S9 to S19, Supplementary Material online). The RAiSD results for the different genetic groups were as follows: for South Polar Skuas, ANTC identified 670 genes and ANTP identified 657 genes; for Chilean Skuas, CHIL identified 567 genes and FKLD identified 640 genes; and for Brown Skuas, NAPF identified 582 genes, NSTF identified 631 genes, and SAPF identified 657 genes. The dataset ANTC, ANTP, and SAPF were the same as dataset South Polar, admixed South Polar (Hyb-SP), and admixed Brown Skuas (Hyb-Br), respectively (supplementary tables S20 to S25; fig. S18, Supplementary Material online).

The distribution of genes under selection varied among chromosomes in terms of number and function. A common pattern was observed among species with a high number of genes under selection with higher µ values distributed between chromosomes 9 and 18, which were generally shared among the 3 species and had broad cellular functions (Fig. 4, black dots, supplementary figs. S19 to S21, Supplementary Material online). Some differences can be observed among species, such as in chromosome 3 for Brown Skua, or visible differences in chromosomes 3 to 6, mainly comparing Brown and Chilean Skua with South Polar Skua. The genes under selection exclusively for each of the 3 species vary in the position in the chromosomes and exhibited more specialized functions (Fig. 4, red dots). For example, the Brown Skuas show genes under selection only for this species mostly located in chromosomes 7 and 8.

By comparing the genes detected through both methodologies, we identified a common set of genes representing regions under strong and differential selection, which have reached or are nearing fixation in the focal populations (supplementary tables S10 to S25, Supplementary Material online). These genes likely indicate key local adaptations, demonstrating increased allele frequency signals (RAiSD) and extended LD patterns (XP-nSL).

After performing different enrichment analyses (RAiSD), this dataset (GO Biological Processes, WikiPathways, KEGG Pathways and Reactome Gene Sets) presents terms related to biological processes that include cellular responses, signaling pathways, and differentiation and development processes, the most statistically significant (LogP) being multicellular organismal processes (GO:0032501), developmental process (GO:0032502), and regulation of biological process (GO:0050789; Fig. 4c). The presence of repeated terms suggests a consistency in the results obtained in the different categories. Particularly in the 3 skua species, processes linked to growth (GO:0040007), reproductive processes (GO:002414), and pigmentation (GO:0043473) are statistically significant (supplementary fig. S21, Supplementary Material online).

Functional enrichment analysis of genes associated with regions exhibiting selection signals revealed that species and admixed species significantly demonstrate gene interactions related to biological processes that may facilitate thermogenic adaptations to extreme polar environments. The nature of these interactions differs depending on the specific case.

In Brown Skuas (NAPF and NSTF), several genes associated with the regulation of lipid metabolism were identified using the RAiSD and XP-nSL methodologies. These genes include SIRT1, KMT2E, TGFBR3, HDAC10, and NCOR1, which interact with the PPARα pathway (KEGG Pathway gga03320; STRING cluster CL:7560) and JMJD1C, KMT2E, NCOR1, HDAC10 involving in regulation of miRNA maturation of lipid metabolism (STRING cluster, CL:7560). Furthermore, whole-body processes related to these genes were also identified (BTO:0001489). Additionally, the interactions between GAL, CHGB, and PCSK2, which are involved in the functions of chromogranin or secretogranin and insulin metabolism, are highlighted (supplementary fig. S23, Supplementary Material online).

In South Polar Skuas (ANTC), using RAiSD analysis we observed genes such as ECI2, ACSBG1, and NUDT7 involved in metabolic processes related to fatty acid degradation (GO:0006631) and metabolic pathways (KEGG Pathway gga000712; supplementary fig. S24, Supplementary Material online). Also, the genes GRIA3 and SHISA9 are associated with the ionotropic glutamate receptor complex and postsynaptic membrane function (GO:0045211; STRING CL:21220; KEGG Pathway gga04080). In regard to genes shared in both methods (RAiSD, XP-nSL), we observed that processes linked to neuronal development (GO:0048667) and the regulation of axonogenesis (GO:0050770) are significantly enriched (supplementary fig. S24, Supplementary Material online).

In Chilean Skuas (CHIL, FKLD), the genes DGKD, PNPLA2, and DGKB are particularly notable for displaying significant results in the RAiSD analysis, as they play vital roles in glycerolipid metabolism (STRING group, CL:10116). Furthermore, the genes DOCK2, DOCK3, and DOCK11 are important for diet-induced thermogenesis (STRING group, CL:21621). This is mediated through the C2 domain present in the proteins Dock180 and Zizimin. These genes are also directly related to IGF1, PAPPA2, and IGFBP3 (STRING group, CL:12939), which are involved in the insulin-like growth factor pathway. Additionally, in genes identified by RAiSD and XP-nSL methodologies, we observe interactions involving LIX1L and PEX11B, associated with peroxisome biogenesis in fatty acid oxidation and lipid biosynthesis. Other relevant genes include GATB and FAAH2, linked to fatty acid degradation processes (supplementary fig. S25, Supplementary Material online).

In the Hyb-Br (SAPF), other notable interactions using RAISD methodology were a significant connection in fatty-acid metabolism with genes RBP7 and C3orf38, linked to the NTF2-like domain superfamily and the lipocalin/cytosolic fatty-acid binding protein family (STRING cluster CL:10516). The enrichment of interactions among proteins with the DnaJ molecular chaperone homology domain (DNAJB4, DNAJC2, DNAJA4, GIPC2) is noteworthy, associated with Mitoguardin and DnaJ C-terminal domain (STRING cluster CL:11528; supplementary fig. S26, Supplementary Material online). When analyzing the shared genes obtained with both RAiSD and XP-nSL methodologies, significant enrichment was observed for genes related to the regulation of cellular response to stress because of an exogenous stimulus (e.g. temperature, humidity, ionizing radiation; GO:0080135; Table 1, Taylor 2000, supplementary fig. S27, Supplementary Material online).

**Table 1:** Results of enrichment analysis, detailing the species studied, selected genes, associated biological processes, and relevant term IDs, along with key references. Each row presents findings for a different species or hybrid group, highlighting gene involvement in specific pathways and developmental processes

Species and Genetic Groups	Method	Highlighted Genes	Main Functions	Reference Publications (PubMed)
Brown Skuas (NAPF and NSTF)	RAiSD	SIRT1, KMT2E, TGFBR3, HDAC10, NCOR1, GAL, CHGB, PCSK2	The regulation of lipid metabolism by PPAR alpha and regulation of miRNA maturation	PMID:25719209 PMID:23359544 PMID: 35393051 PMID: 27535581
	Shared (XP-nSL & RAiSD)	JMJD1C, KMT2E, NCOR1, HDAC10		
South Polar Skuas (ANTC)	RAiSD	ECI2, ACSBG1, NUDT7	Fatty acid metabolic process	PMID:25719209 PMID:23359544 PMID:30154830 PMID: 35393051 PMID: 11896043
	…	GRIA3, SHISA9	Ionotropic glutamate receptor complex, postsynaptic membrane function	
	…	HOXC13, HOXC12, DNAJB4, DNAJA4, DNAJC5B	Hox genes, heat shock protein	
	Shared (XP-nSL & RAiSD)	DSCAM, ROBO2, MYCBP2, ATP8A2, CNTN5	Axonogenesis, cell morphogenesis involved in neuron differentiation	
Chilean Skuas (CHIL, FKLD)	RAiSD	DGKD, PNPLA2, DGKB. Dock180, Zizimin DOCK2, DOCK3, DOCK11. IGF1, PAPPA2, IGFBP3.	Diet-induced thermogenesis Insulin metabolism, Granin (secretogranin)	PMID: 31474366 PMID:30781724 PMID:33869165 PMID: 2053134 PMID: 27238638
	Shared (XP-nSL & RAiSD)	LIX1L, PEX11B. GATB, FAAH2	Fatty acid biogenesis and degradation	
Brown-HYB	RAiSD	SIN3A, KMT2E, HDAC10	Transcriptional regulation, chromatin assembly, epigenetic modifications	PMID:11041354
	…	LHX2, TBX5, HOXA13, HOXC13, IRX3, TBR1, SOX1, HOXC9, OTX1, WNT7A, KITLG, WNT8B. BRAF, RAF1, MOS, MAP2K. AURKA, MIOS, ZP4.	Wnt signaling pathway, the Hedgehog signaling pathway, the homeodomain, melanogenesis	
	Shared (XP-nSL & RAiSD)	NEO1, TNR, SMCHD1, BFAR, VPS13C, FMN2, HDAC10, ARID2, SPRED2	Regulation of cellular response to stress	PMID: 11299523
	…	ATRX, HOXA9, LRP2, SALL1, PCYT1B	Reproductive structure development, gonad development, development of primary sexual characteristics	
South Polar-HYB	RAiSD	ACSBG1, DCAF5, ETFA	Long-chain fatty acid metabolism in the brain and myelin formation	PMID: 36588745
	Shared (XP-nSL & RAiSD)	AGL, PPP2R2C, PPP2R3B, NFATC3	Glycogen synthesis and degradation	PMID: 7470349

In Hyb-SP (ANTP), the RAiSD methodology shows that several genes play important roles in several fatty acid metabolic processes. For example, the genes ACSBG1, DCAF5 and ETFA are linked to the metabolism of very long-chain fatty acids in the brain and the formation of myelin (GO:0035338, GO:0035336, GO:0046949, Table 1, Hearing 2000), as well as the biosynthesis of fatty acids (GO:0042304). Among the shared genes obtained with both methodologies (RAiSD, XP-nSL) we can observe a group involved in glycogen synthesis and degradation, processes related to the generation of metabolic energy and directly related to fatty acids (Stanley 1981; supplementary fig. S28, Supplementary Material online).

The enrichment of HOX genes is notably observed in the groups of species studied. In particular, the South Polar Skuas showed significant interactions between HOXC13 and HOXC12 genes with isoforms of the DnaJ chaperone protein, including DNAJB4, DNAJA4, and DNAJC5B. These proteins regulate mitochondrial dynamics through the Mitoguardin protein (STRING cluster, CL:12623; supplementary fig. S24, Supplementary Material online). The observed dynamics in RAiSD analysis of Hyb-Br (SAPF) suggest that a group of genes (LHX2, TBX5, HOXA13, HOXC13, IRX3, TBR1, SOX1, HOXC9, OTX1, WNT7A, KITLG, WNT8B) has been interacting with clusters associated with the Wnt signaling pathway, the Hedgehog signaling pathway (CL:13460), the homeodomain (CL:13739), and melanogenesis (KEGG Pathway: gga04916; supplementary fig. S27, Supplementary Material online). In addition, genes are significantly enriched for promoting reproductive biological processes such as BRAF, RAF1, MOS, and MAP2K, notably involving progesterone-mediated oocyte maturation (KEGG Pathway gga04914). Another group of genes (AURKA, MIOS, and ZP4), which are linked to the regulation of meiosis for oocyte development (GO:0048599), and the regulation of sperm binding to the zona pellucida (GO:2000359), were also observed to be enriched. In examining the genes common to both methodologies (RAiSD and XP-nSL), the HOXA9 gene's association with reproductive processes can be emphasized, particularly its roles in reproductive structure development (GO:0048608), gonad development (GO:0008406), and the development of primary sexual characteristics (GO:0045137; supplementary fig. S29, Supplementary Material online).

### Ecological Niche Modelling

To evaluate biogeographic redistribution processes linked to global change, future scenarios of range occupancy were generated with correlative Species Distribution Models (SDMs). Models generated depict the change in occupancy for both the hybrid and non-hybrid subpopulations, as well as both combined (supplementary fig. S30, Supplementary Material online).

**Fig. 5.**
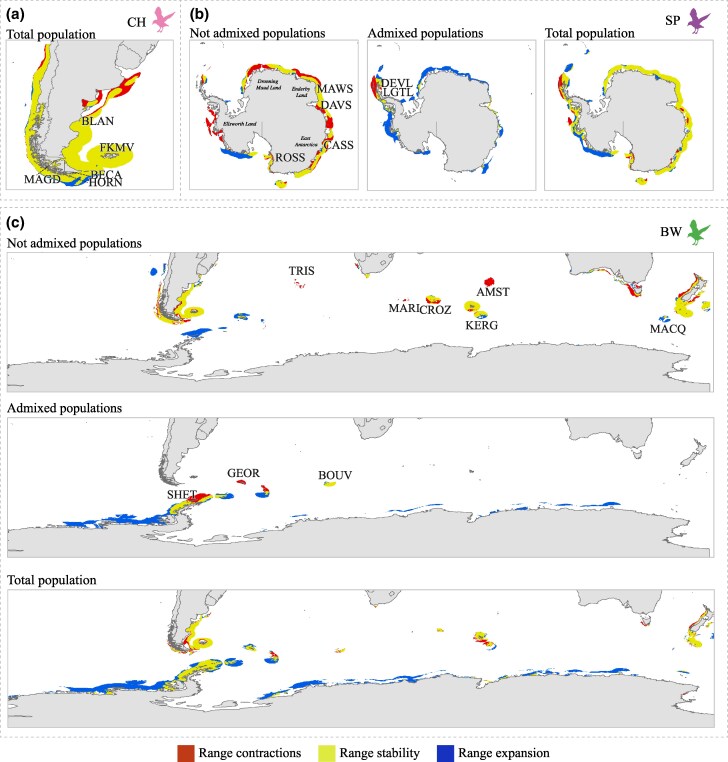
Ecological niche modelling (ENM) of the southern skua complex's potential areas of occupancy changes between present and 2050 RCP 8.5 conditions. Range contractions are shown in red, range stability in yellow, and range expansion in blue. a) ENM range change for Chilean Skuas. b) ENM range change for South Polar Skua populations. c) ENM range change for Brown Skua. The 4-letter acronyms indicate the sampling sites included in this study.

The Chilean Skua occupancy range is projected to remain relatively stable until 2050 even in the scenario of high carbon emissions (RCP 8.5); yet, the northernmost parts of the distribution will be lost, with almost no counter gain in the south due to the absence of suitable coastal habitat to breed south of Tierra de Fuego (Fig. 5a). The overall range change for the Chilean Skua was negative, ranging from −9% in 2050 RCP 2.6 to −27% in 2100 RCP 8.5 (supplementary tables S26 and S27, Supplementary Material online).

The non-hybridized populations of South Polar Skua will retain some of their range but show a contraction within Continental Antarctica, such as Ellsworth Land, Dronning Maud Land, Enderby Land and East Antarctica. In contrast, hybridized populations of the South Polar Skua are predicted to expand around Continental Antarctica. The overall range of the combined South Polar Skua population was predicted to remain stable across the Antarctic Peninsula and Continental Antarctica; however, detailed analysis reveals that this is because of the expansion of hybrid individuals, not the non-hybridized populations (Fig. 5b). The overall range change for the South Polar Skua is predicted to be positive, from 50% in 2050 RCP 2.6% to 150% in 2100 RCP 8.5 (supplementary tables S26 and S27, Supplementary Material online).

Non-hybridized populations of Brown Skua are predicted to decrease in subtropical areas including around Amsterdam Island and Tristan da Cunha and increase in the north-west Antarctic Peninsula and Scotia Sea. However, hybridized populations of Brown Skua will expand farther south in the Antarctic Peninsula and some areas of Continental Antarctica. The combined population of Brown Skuas was predicted to colonize Continental Antarctica, likely increasing competition or introgression with the South Polar Skua (Fig. 5c). The overall range change for the Brown Skua was predicted to be negative, from −1% in 2050 RCP 2.6 to −23% in 2100 RCP 8.5 (supplementary tables S26 and S27, Supplementary Material online).

## Discussion

Hybridization between species can provide insights into patterns of introgression and local adaptations that may arise due to positive selection, which may generate new adaptive phenotypes (Abbott et al. 2013). The adaptive allele maintained by selection following consecutive hybridization and backcross introgression events is highly dependent on the environment (Schmickl et al. 2017). In this context, Southern Hemisphere skuas have become an important focus of study due to phylogenetic uncertainties (Mikkelsen and Weir 2023), their relatively recent speciation (Ritz et al. 2008), and evidence of the high level of hybridization in the area of breeding sympatry at the Antarctic Peninsula (Ritz et al. 2006). Our results reveal clear patterns of introgressive hybridization among the 3 skua species and indicate that the region of genetic admixture is considerably broader than previously documented (Ritz et al. 2006, 2008; Carneiro et al. 2010; Costa and Alves 2012). Based on the demographic and evolutionary history established in our study, we support the speciation scenarios of the 3 species, and emphasize the relative importance of introgression for their adaptation. While, our results do not completely affirm the established taxonomy, they support the recognition of the 3 species, but with some modification relative to previous schemes (i.e. reassignment of Falklands Brown Skua). Building on our findings regarding phylogeny and gene flow, we explored the extent of adaptation and introgression in skuas. Our results reveal that skua species have developed multiple adaptation mechanisms in response to past environmental challenges. These mechanisms include morphological, physiological, and behavioral changes that enhance their survival under extreme environmental conditions (Mota-Rojas et al. 2021).

### Evolutionary History, Taxonomic Delimitation and Biogeography

To understand introgressive hybridization patterns, one must first determine the evolutionary history of the clade and resolve how many taxa have contributed to hybridization. The 3 different species of skua were recovered as reciprocal monophyletic clades in the ML phylogeny, with fine scale structure recovered among populations within each species. Our divergence time estimated during the last 200 kya is consistent with the TMRCA for skuas derived from mtDNA, approximately 219 to 130 kya (Ritz et al. 2008). During the Last Glacial Maximum (LGM), we observed distinct population trajectories: Brown Skua populations north of the Antarctic Polar Front (APF) experienced sustained growth, while other populations decreased. The overestimation of effective population size (Ne) in admixed individuals is a common issue in sequentially Markovian coalescence-based models like PSMC (Hawks 2017). Consequently, the observed waves of Ne increase in the admixed populations does not necessarily mean larger Ne. The timing of this Ne expansion suggests that hybridization might have occurred after the Last Interglacial Period (LIG). Our estimates of historic Ne trajectories indicate that the Ne of skuas at Amsterdam Island has historically been much lower compared to other Brown Skua populations. Thus, the observed decline at Amsterdam Island around 50 kya may reflect either the time of population isolation or a bottleneck associated with its colonization of the island.

Our results reveal taxonomic inconsistencies, especially in relation to the previous assignment of the skuas at the Falkland/Malvinas Islands as a subspecies of the Brown Skua, *S. antarcticus antarcticus*. Our data show conclusively that these correspond to Chilean Skuas and should be designated as *S. chilensis antarcticus.* This finding highlights the importance of the APF as a biogeographic barrier that isolates Chilean Skuas from Brown and South Polar Skuas. Such biogeographic barriers are considered to be a key driver of speciation in other seabirds, including the gentoo penguin (*Pygoscelis papua*) in the same region (Pertierra et al. 2020). Our data also suggest that the skuas at Amsterdam Island may warrant subspecies status.

Previous phylogeographic studies using mtDNA failed to distinguish among skua species, often suggesting either a species complex or a single taxon (Ritz et al. 2006; Mota et al. 2023). Similarly, genomic data has shown a polytomy or incongruent phylogenetic topology (Mikkelsen and Weir 2023). In contrast, our phylogenetic analysis indicates that Chilean Skuas are sister to a clade comprising Brown and South Polar Skuas, which are recovered as sister species. This result aligns with geoclimatic studies of Patagonia and the Falkland/Malvinas Islands, which indicate that during the Last Glaciation, southern Chile experienced very cold and dry conditions, accompanied by glacial fluctuations of limited extent (Mardones et al. 2011), whereas the Falkland/Malvinas Islands were never intensively glaciated, with only ice dome formation at higher elevations (McDowall 2005).

Based on morphological data, the Falkland Skua occurs not only on the Falklands/Malvinas Islands but also along the north coast of Argentina (Punta Tombo and Camarones) in the Atlantic Ocean. In contrast, Chilean Skua is predominantly located on the southern coast of Tierra del Fuego and the Pacific coast of Chile (Devillers 1978). Notably, samples collected from Blancas Islands, near Camarones, were morphologically identified as Chilean Skua and clustered genetically with Falkland/Malvinas Island Skuas, slightly distinct from those in the southernmost regions. The intraspecific divergence between Chilean Skuas from Falkland/Malvinas Island and Blancas Islands from the Chilean Skua locations on the continent is comparable to the divergence between Brown Skuas from Amsterdam and Tristan da Cunha Islands compared to all other populations. Tristan da Cunha and Amsterdam Islands are grouped together and isolated by the Subtropical Front (STF) from other Brown Skua breeding populations. Since Tristan Skua are classified as a distinct subspecies (*S. a. hamiltoni*), skuas from Amsterdam Island should be considered part of this taxonomic group. The Falkland/Malvinas Current and STF are crucial in isolating Chilean and Brown Skuas, respectively, analogous to the population differentiation observed in southern rockhopper penguins (*Eudyptes chrysocome*) between the Falklands/Malvinas and the South American continent, and between northern rockhopper penguins (*E. moseleyi*) north and south of the STF (Frugone et al. 2018; Lois et al. 2020). The distribution of northern rockhopper penguins mirrors that of skuas north of the STF, with similar patterns observed in Amsterdam and Tristan da Cunha Islands.

### Adaptive Selection in Extreme Cold Environments

Divergent selection promotes distinctive biological traits in species which can be observed in the genomes of the 3 skuas and admixed individuals (Fig. 4). This source of variation can be generated by de novo mutations, existing genetic variation, or genetic introgression events, modifying the evolutionary dynamics of a trait (Campagna and Toews 2022).

In extreme environments, such as the cold and windy climates found in subtropical islands and subantarctic and Antarctic regions, the genetic basis of adaptive phenotypes allow us to identify the direction of gene flow between species, which genes are unique to each species, and which of them are maintained by selective pressures in hybrids. Thermoregulation is a vital physiological process that enables survival in extremely cold regions. In this context, the species studied display selection signals that appear to enhance their ability to endure extreme temperatures, prolonged periods without food, and various environmental pressures.

In Brown skuas, candidate genes were found to be enriched in pathways associated with miRNA-regulated PPARα fatty acid biosynthesis. Li et al. (2016) showed that specific miRNAs significantly influence lipid accumulation in chickens and act as regulators in both steroid biosynthesis and the synthesis of unsaturated fatty acids. Since adipose tissue plays a crucial role in regulating energy balance, the interactions among the identified genes could enhance the regulation of physiological processes, including appetite and satiety, fat distribution, insulin sensitivity, reproduction, and immune response (Daković et al. 2014). Bornelöv et al. (2018) explored these potential relationships and demonstrated a direct link between gene expression in visceral fat and both the reproductive system and energy balance.

The enrichment of HOX, Wnt, and Hg genes in South Polar Skuas, along with processes linked to fatty acid metabolic, suggests a role in promoting the differentiation of white and brown adipose tissues, critical for maintaining body temperature in harsh conditions. Studies in humans (Karastergiou et al. 2013) indicate that certain HOX genes regulate this differentiation, supporting the inference of a similar mechanism in the studied skua species. These candidate genes are likely to experience selective pressure in Antarctic environments, enhancing survival capacity by improving thermogenesis.

In Chilean Skuas, the genes DOCK2, DOCK3, and DOCK11 play a crucial role in diet-induced thermogenesis and are associated with the insulin-like growth factor pathway. In humans, brown adipose tissue (BAT) activation has been linked to improved whole-body insulin sensitivity (Lee et al. 2013; Chondronikola et al. 2014). Similarly, studies in rodents have shown that cold acclimation increases BAT activity and decreases fasting glucose concentrations, an indirect measure of insulin sensitivity (Hanssen et al. 2015). These findings suggest a potential link between BAT function and enhanced insulin sensitivity, indicating a possible metabolic advantage in Chilean Skuas surviving in their ecological niche.

Genes associated with neuronal cold perception and fatty acid metabolism were enriched in both South Polar and Hyb-SP skuas (Barnes-Vélez et al. 2022). The brain, a highly metabolic organ, plays a critical role in generating heat and maintaining metabolic homeostasis in extreme cold (Thornton 2003; Howarth et al. 2012; Yu et al. 2012). The expression of genes involved in lipid metabolism in cold-sensitive brain regions suggests adaptations that prevent fatty acid toxicity and maintain neuronal function. Min et al. (2025) highlight how cold acclimation modulates the expression of lipolytic and thermogenic enzymes in these regions, ensuring metabolic balance and promoting survival. Moreover, studies in mice (Tao et al. 2015; Liang et al. 2019) demonstrate that chronic exposure to cold induces adipogenesis in subcutaneous white adipose tissue. The presence of positive selection signals in genes related to fatty acid metabolism further underscores their essential role in thermogenesis.

In Hyb-Br skuas, heat shock proteins of the DnaJ family seem to modulate cellular stress caused by temperature fluctuations. These proteins facilitate cell recovery under heat stress conditions, a process analogous to that seen in mammals subjected to non-lethal heat stress (Jäättelä 1999). Studies in mice reveal that the absence of a heat shock factor such as Hsf1 reduces the expression of Ucp1, impairing thermogenesis in white and brown adipose tissues (Sonna et al. 2002). This evidence suggests that the DnaJ proteins in Hyb-Br skuas may directly contribute to heat generation and survival under extreme cold conditions.

A further distinction in Hyb-Br skuas involves the enrichment of putative genes related to reproductive success (e.g. ATRX, HOXA9, LRP2, SALL1, PCYT1B), including the development of reproductive structures, gonads, and primary sexual characteristics. This suggests selective pressures driving adaptations to reproductive processes alongside thermoregulation in this population. León et al. (2024) have suggested that signals of positive selection on genes could be related to reproductive isolation mediated by temperature during the recent speciation of the Galápagos penguin (*Spheniscus mendiculus*) from the Humboldt penguin (*S. humboldti*).

The interaction between thermoregulation, energy metabolism, and reproductive isolation reflects the complexity of genetic adaptations in the skua species studied. These findings underscore the critical role of selective pressures in shaping adaptive phenotypes under extreme environmental conditions. Furthermore, the contrasting genetic enrichment between Hyb-Br and Hyb-SP skuas demonstrates how environmental factors and mechanisms of reproductive isolation influence speciation processes.

### Future Scenario for Southern Skuas and Hybrid Zone

SDM suggests changes in the species ranges of all 3 skua species, their overlap, and the future extent of the area occupied by hybrid individuals. Introgression might increase with higher species overlap resulting from the expansion of Brown Skuas into the South Polar Skua distribution, which was also predicted when modelling the distribution of the introgressed individuals. This forecast aligns with the analysis of the genes under selection for each of the species where the South Polar Skuas and the Brown Skuas were the taxa that presented the greatest differentiation of GO terms related to physiological processes associated with their adaptation to the extreme Antarctic environment. Even introgressed genes under selection from gene flow between both species could be identified in the hybrids. However, Brown Skuas will be strongly affected in the north of the STF, and are predicted to disappear in the Tristan da Cunha and Amsterdam Islands, which are different taxa from the remaining locations. This is consistent with the genomic results of the skuas from Tristan da Cunha and Amsterdam Island, which already show lower genetic diversity and smaller effective population size. Therefore, the possible increase in the extent of the area of introgression is reflected in an overall positive increase in Brown Skuas (negative change in the incidence of South Polar Skuas).

Expansion of introgression areas can trigger an erosion of differentiation between species, through increased introgressive hybridization due to the weakening of previously stratified divergent selection regimes. This can seriously affect local native ecological adaptations and strategies that appeared during speciation. This meltdown may be reinforced by additional changes in abiotic and biotic environmental conditions capable of altering the direction, strength and form of selection that generates and maintains species differences and coexistence, particularly between recently divergent species. Overall, the balance between disruptive selection and homogenization via gene flow will determine the degree of genetic differentiation achieved and its genomic distribution (Taylor and Larson 2019; Quilodrán et al. 2020). The selective pressures on genes associated with reproductive isolation in admixed individuals (Hyb-Br) appear to influence the dynamics of the hybrid zone's expansion. On one hand, reproductive barriers that act during the embryonic stage may restrict hybrid expansion, helping to maintain species boundaries and preventing genetic homogenization. On the other hand, as hybrids spread into new environments, such as Antarctica, there may be increased selective pressure to reduce reproductive barriers. This adaptation could enhance hybrid viability and fertility, enabling the hybrid population to expand more effectively into previously unoccupied niches.

The predictions of the macroclimatic SDMs must be taken with caution as the results may overpredict the potential range by disregarding complex abiotic processes such as new competition among species or shifts in prey availability; yet, these correlative techniques are used to indirectly account for secondary biotic effects. Nonetheless, Brown Skuas could face an even more significant impact because they breed on subantarctic islands and depend primarily on terrestrial resources such as penguins for their diet for successful reproduction (Pietz 1987; Parmelee 1988). In the Antarctic Peninsula, current research has predicted an even more significant impact and a decline in the colonies of species such as the Adélie (*Pygoscelis adeliae*) and Chinstrap penguins (*P. antarcticus*) that are found in the same habitat (Cimino et al. 2016; Strycker et al. 2020; Schmidt et al. 2023). Furthermore, although positive changes are observed in the general distribution of the South Polar Skua, possibly due to its adaptation to the extreme Antarctic environment, its reproductive success throughout Antarctica is closely linked to marine resources (Young 1963; Parmelee 1988; Mund and Miller 1995). This success could be compromised by the reduction in krill growth, influenced by changes in ocean temperatures and the ecological impacts derived from the fishery in the Southern Ocean (Veytia et al. 2020).

## Materials and Methods

### Sample Acquisition

We collected carcasses, shed feathers, or blood samples from breeding adults or chicks from the entire distribution of *Stercorarius* in the Southern Hemisphere. We generated high-coverage whole genome resequencing data from 105 skua individuals and complemented these data with 6 publicly available whole genomes recovered from the NCBI Sequence Read Archive (SRA; Mikkelsen and Weir 2023). The compiled dataset consisted of 111 skua individuals from 21 geographic locations (Fig. 1 and supplementary tables S1 and S2, Supplementary Material online). The genome of the razorbill (*Alca torda*), a phylogenetically close charadriiform species, was included as an outgroup [SRR25384210, SRR25384211, SRR25384217, SRR25384218] (Černý and Natale 2022).

Adult skuas were captured by hand or with long-handled nets, sampled and then released at the capture site. Blood samples were taken from the brachial vein or external metatarsal vein and stored on FTA cards or in tubes with 96% ethanol. Only one chick per territory was sampled, and samples were not taken from parents of sampled chicks. Handling times were always < 15 min, and usually < 10 min.

### DNA Isolation and Whole Genome Sequencing

High-quality genomic DNA was isolated from tissue samples following a salt extraction protocol (Aljanabi and Martinez 1997) with minor modifications (see supplementary Text, Supplementary Material online) and delivered to MedGenome (Delaware, USA) for library preparation and whole genome sequencing. Samples showing no DNA degradation and at least 50 ng/μL concentration were selected for library construction using the TruSeq DNA Nano High Throughput Library Prep Kits (Illumina, cat. 20015965) following the manufacturer's instructions. Whole genome sequencing (∼10 to 20 GB per sample) was performed using the 150 bp pair-end protocol on the Illumina NovaSeq6000 platforms.

### Data Generation and Processing

The number of unique reads and initial quality of the raw sequences were assessed using FastQC (version 0.11.9; Andrews 2010). Based on the quality reports generated, the reads were preprocessed by trimming the adapter sequences and low quality bases using Fastp (version 0.23.2; Chen et al. 2018) with the default settings. To verify that filtering was performed successfully, we evaluated the read quality of sequencing with FastQC and visualized the results with MultiQC (version 1.13; Ewels et al. 2016).

Initially, we generated 2 working datasets by separately mapping trimmed reads from each individual to 2 different reference genomes using the BWA-MEM2 tool (version 0.7.17-r1188; Vasimuddin et al. 2019) with default settings. The first dataset, referred to going forward as the “razorbill” dataset, consisted of reads from all skua samples plus reads from the razorbill as an outgroup, mapped to the razorbill reference assembly “bAlcTor1″ (GenBank assembly accession: GCA_008658365.1). Because the razorbill, a member of the Alcidae, is the closest species to Stercorariidae (Černý and Natale 2022) with an available chromosome-level reference genome, it was used for population structure and genetic differentiation analyses. Razorbill was not used for selection analyses, as it did not have publicly available gene annotation required as of September 2024. Therefore, a second dataset was produced (“kittiwake” dataset), mapping all skua samples to the black-legged kittiwake (*Rissa tridactyla*) reference assembly “bRisTri1” (RefSeq assembly accession: GCF_028500955.1; Sozzoni et al. 2023). The black-legged kittiwake is a less closely related shorebird (Černý and Natale 2022), but possess an available annotated chromosome-level reference genome associated with a gene annotation file, both required for the positive selection analyses described below.

We used SAMtools (version 0.1.18; Danecek et al. 2021) to convert the resulting SAM files into sorted and indexed BAM files, keeping only high-quality properly paired reads. After marking potential PCR duplicates with the option “MarkDuplicates” in Picard (version 2.27.4; http://broadinstitute.github.io/picard/), thereby enabling downstream tools to ignore these reads, we implemented the Genome Analysis Toolkit (GATK; version 4.1.7.0; McKenna et al. 2010) to perform local realignment of reads near insertion and deletion polymorphisms. We first used the “RealignerTargetCreator” tool to identify regions where realignment was needed, then produced a new set of realigned BAM files using the “IndelRealigner” tool. The final mean coverage and coverage distribution across each reference genome were assessed using QualiMap2 (version.2.3; Okonechnikov et al. 2016). The mean sequencing coverage was 16.2× (±7.9) using the razorbill genome as a reference and 11.3× (±5.4) using the black-legged kittiwake as a reference. The relative coverage of the Z chromosome was used to identify the sex of the individuals, revealing that the dataset comprised of 58 male and 53 female skuas (supplementary table S3, Supplementary Material online).

### Variant Discovery and Filtering

SNPs were called per chromosome for the razorbill and kittiwake datasets using the BCFtools “mpileup” option (-q 30 -Q 30 -a SP, DP, AD options) and the BCFtools “call” (-f GQ -mv options) option implemented in BCFtools (version 1.13; Danecek et al. 2021). The variant calling of the razorbill dataset resulted in 26 variant call format (VCF) files containing a total of 90,761,514 SNPs distributed across 25 autosomes and the Z sex chromosome. The variant calling of the kittiwake dataset resulted in 32 VCF files containing a total of 110,609,108 SNPs distributed across 30 autosomes and Z and W sex chromosomes.

The VCF files generated for individual chromosomes in each dataset were concatenated and indexed using the “concat” and “index” functions of BCFtools, respectively. Sex chromosomes were excluded from all datasets. The resulting merged VCF files for each dataset were normalized using the BCFtools “norm” function and further filtered with specific VCFtools (version 0.1.17; Danecek et al. 2021) parameters to meet the requirements of the different analyses (see supplementary Text, Supplementary Material online). A detailed description of the number of individuals, number of localities and SNPs used for different analyses can be found in the supplementary Material (see supplementary Text; table S4), Supplementary Material online.

### Genomic Population Structure and Differentiation

We first investigated the population structure among all 111 Southern Hemisphere skua samples with a data set of 992,654 unlinked biallelic SNPs with 5% missing data, derived from filtering the razorbill dataset. A PCA on the genomic data was performed using PLINK2 (version 2.00a2; Purcell et al. 2007), followed by a Tracy–Widom test implemented in LEA (version 3.10.2; Frichot and François 2015) to determine the significance level of the eigenvectors. The first 3 most significant principal components were plotted using the R package (version 4.2.3; https://www.r-project.org/) ggplot2 (version. 3.3.6; Wickham 2011). Individual ancestry proportions were estimated using a maximum likelihood (ML) approach implemented in ADMIXTURE (version 1.3.0; Alexander et al. 2009) by setting the number of ancestral populations (K) from 1 to 7 (supplementary table S5, Supplementary Material online). The mean cross-validation (CV) error for each value of K was used to select the optimal number of ancestral populations (K). The R package ggplot2 and the Ancestry Painter (version 5.0; Feng et al. 2018) software were used to visualize and organize the ADMIXTURE results.

Based on the patterns of population structure observed among all individuals, additional PCA and ADMIXTURE analyses were performed on the originally identified groups to inspect the variation within them. The first group contained all individuals of the South Polar Skua (*n* = 37), the second group contained all individuals of the Brown Skua (*n* = 51), and the third consisted of tightly clustered Chilean and Falkland Skua (*n* = 23).

We used RAxML-NG (v. 1.1; Kozlov et al. 2019) to investigate genealogical relationships among skuas. To achieve this, we converted a LD-filtered VCF derived from the razorbill dataset to the PHYLIP format using the vcf2phy.py script (https://github.com/edgardomortiz/vcf2phylip). Subsequently, we employed the ascbias.py script (https://github.com/btmartin721/raxml_ascbias) to remove all invariant sites and performed ascertainment bias corrections. RAxML-NG was run using the GTR + ASC_LEWIS model and 500 bootstrap replicates and plotted using the FigTree software (version v1.4.4; https://github.com/rambaut/figtree/releases). The samples from Blancas Islands (BLAN), Hornos Island (HORN), and Chatham Island (CHAT) were excluded from this analysis due to the low number of individuals. The individuals from Ross Sea (ROSS) were excluded due to low coverage.

Based on the topology of the ML tree (supplementary fig. S6, Supplementary Material online) and the global genetic structuring given by the PCA and ADMIXTURE analysis, we defined 7 phylogeographic groups for additional analyses (see population genetic structure and differentiation results).

Estimates of pairwise genetic differentiation (F_ST_), heterozygosity and Tajima's D were calculated with a LD-filtered VCF derived from the razorbill dataset. The SNP-based heterozygosity and the average Weir and Cockerham pairwise F_ST_ (Weir and Cockerham 1984) were calculated with VCFtools. These analyses were conducted for 2 different datasets: one with samples grouped by locality and the other by phylogeographic group. Tajima's D was calculated only for the phylogeographic groupings with the “tajima” function (vk tajima 50,000 25,000) of the utility program VCF-kit (https://vcf-kit.readthedocs.io/en/latest/, accessed December 2023). Finally, we used the VCFtools “snpden” function with a window size of 1Mb to identify the density and distribution of heterozygous SNPs across the whole genome of one high coverage (14 to 15×) representative individual per phylogeographic group. Prior to running VCFtools, a filtered VCF with 0% missing data was split into samples using the BCFtools “view” option. The SNP density plots were generated in R using a custom script (https://github.com/henriquevf/snpden_plot).

### Reconstruction of Demographic History

We used the PSMC approach (v.0.6.5-r67; Nadachowska-Brzyska et al. 2016) to infer historical population sizes and divergence times of the skuas based on whole genome sequences. We followed the general pipeline suggested by the developer to produce input files. The scaffolds, mitochondrial sequences, and sex chromosomes were removed from the consensus sequences using the Seqtk tool (version 1.4-r122; https://github.com/lh3/seqtk), keeping only 25 autosomes. PSMC was carried out with parameters set as –N25 –t15 –r5 –p “4 + 25*2 + 4 + 6″ for all individuals. To avoid biases due to differences in genome coverage, we repeated PSMC analyses on selected individuals with mean coverage between ∼14 and 15× of each population to generate consensus sequences. To increase the number of individuals that meet this criterion, we subsampled those with read alignment coverage greater than 15× using SAMtools. To estimate the uncertainty in the estimates of changes in the effective population size over time, 50 bootstraps were performed for one representative individual per population.

PSMC outputs were visualized in R, with an average generation time of 6 or 12 yr, assuming generation time from age at first reproduction, which has been shown to predict generation time (Lehtonen and Lanfear 2014) and a mutation rate per site per generation (μ) estimated for 3 closely related avian species: Atlantic puffin (μ = 1,7125×10^−9^; *Fratercula arctica*; Kersten et al. 2023), Southern giant petrel (μ = 5.9×10^−9^*; Macronectes giganteus*; Kim et al. 2021), and Northern fulmar (μ = 2.89 × 10^−9^*; Fulmarus glacialis*; Nadachowska-Brzyska et al. 2016). Results were scaled using a generation time of 6 yr with the mutation rate calculated by Kim et al (2021), as estimated using sequence divergence based on comparisons between Northern fulmar and Southern giant petrel genomes. Additionally, key glaciation events were represented, including the penultimate glacial maximum (PGM, 194–130 ka; Stauch and Lehmkuhl 2010), last interglacial period (LIG, 129–116 ka; Barnett et al. 2023), last glacial period (LGP, 12–110 ka; Gómez et al. 2022), and last glacial maximum (LGM, 26.5–19 ka; Wilmes et al. 2023).

### Footprint of Introgression and Gene Flow

To further evaluate population splits and historical gene flow between the 7 phylogeographic groups, an unrooted ML phylogenetic tree was inferred using TreeMix (version 1.13; Pickrell and Pritchard 2012). The allele frequency input file for TreeMix was generated using the vcf2treemix.sh script (https://github.com/speciationgenomics/scripts/blob/master/vcf2treemix.sh). For this analysis we used a LD-filtered VCF containing only high coverage sequencing data (∼14 to 15×) derived from the razorbill dataset. The optimal value of migration events (m) in population trees was inferred from the second-order rate in likelihood (Δm) across incremental values of m using the OptM package in R (version 0.1.6; Fitak 2021). To generate the likelihood files analyzed using the default Evanno method implemented in OptM, we ran TreeMix for 10 independent replicates varying the value of m from 1 to 7, with a global set of rearrangements (-global) and a randomly selected window size (k) of between 100 and 1000 SNPs in 50 SNP increments. Then we followed the bootstrap procedure implemented in the R package BITE (version 2.0.0; Milanesi et al. 2017) and the publicly available scripts on the GitHub website (https://github.com/carolindahms/TreeMix) to perform 30 independent TreeMix runs, each using a new random seed, the optimal estimated value of migration events (*m* = 3), 100 SNP windows (k), and a consensus tree obtained with PHYLIP (version 3.697; Felsenstein 1993) using a starting tree (-tf) derived from the consensus of 100 bootstrap pseudoreplicates. Finally, the matrices representing the residuals and drift estimates, as well as the final tree with the highest likelihood, were plotted in R using the BITE package, indicating bootstrap values for each node and migration weight.

To detect regions with genetic introgression across the genome, we estimated Patterson's D statistic (Durand et al. 2011) and related Dsuite statistics (version 0.5 r52; Malinsky et al. 2021). The “Dtrios” tool implemented in Dsuite was used to calculate the sum of 3 different patterns (BABA, BBAA, and ABBA) and the D and f4 ratio statistics for the 35 possible triplets (supplementary table S6, Supplementary Material online). Furthermore, we used the “Fbranch” tool in Dsuite to estimate the introgression rate for each putative introgression event using the f-branch statistic (fb) and detect gene flow involving internal or terminal branches when the level of gene flow was greater than ∼1% (supplementary table S7, Supplementary Material online). We then visualized the results using Dsuite's *dtools.py* script.

### Genomic Signatures of Adaptation

To identify genomic regions under positive selection, we analyzed a normalized VCF (kittiwake dataset) generated by performing variant calling of all skua samples mapped to the black-legged kittiwake reference assembly “bRisTri1” (GenBank assembly accession: GCF_028500815.1). To assign functional information to the variants, biallelic SNP sites were annotated using snpEff (version 5.2; Cingolani et al. 2012).

To identify signatures of positive selection in the skuas, 2 complementary approaches were applied: RAiSD and XP-nSL. The analysis with RAiSD (Raised Accuracy in Sweep Detection; version 2.9, Alachiotis and Pavlidis 2018) was performed to detect selective sweeps within each species and genetic group independently, using as a basis a set of filtered and biallelic variants derived from whole genome data. RAiSD calculates the metric μ, based on the combination of multiple genomic signatures associated with positive selection, such as the allele frequency spectrum (SFS), LD, and variance in nucleotide diversity (VAR). This approach allowed to identify genomic regions under strong and fixed selection in each species and for each genetic group. Results were filtered using a 99.7 percentile threshold to select the most statistically significant signals and default window sizes of 50 Kb (empirically determined).

XP-nSL (Cross-Population Nucleotide Site Loss; version 2.0.2, Szpiech and Hernandez 2014) was used to detect signals of differential selection between populations by comparing extended LD (EHH) in a focal population with respect to a reference population, allowing the identification of differentially selected long haplotypes, even in early stages of a selective sweep. Comparisons were made focusing on each of the hybrid populations (South Polar Hybrid and Brown Hybrid) against their parental species (South Polar and Brown) and between both species from polar and temperate climates. The results were filtered using a 99.7 percentile threshold to select the most statistically significant signals and considering the statistical value of F_ST_ between the genetic groups of study to link genetic differentiation (F_ST_) with specific selection patterns detected by XP-nSL. This approach allows the detection of differentially selected long haplotypes, even at early stages of positive selection. Unlike RAiSD, XP-nSL does not require specifying genomic windows, as it operates directly on the continuity of haplotypes associated with SNPs.

For both RAiSD and XP-nSL, the coordinates of the selected regions were converted to BED format and compared with genomic annotations to identify known genes (without considering unannotated regions) associated with selection signals using BEDTools “intersect” option (version 2.18; Quinlan and Hall 2010). In addition, the results of XP-nSL and RAiSD were superimposed to identify common regions under strong and differential selection. These strategies allowed to explore the genetic basis of local adaptations and the selective processes that shape parental and hybrid populations.

To identify the underlying biological processes and cellular functions of genes under selection, gene ontology (GO) enrichment analysis was performed (www.geneontology.org) using the PANTHER classification system (version 18.0; www.pantherdb.org; Mi et al. 2013). Furthermore, to validate the functional importance of the selected genes and to better understand the relationships between them, further enrichment analysis was carried out using metabolic pathways from the Kyoto Encyclopedia of Genes and Genomes (KEGG) database (https://www.genome.jp/kegg/pathway.html; Kanehisa and Goto 2000). A third enrichment was performed using the STRING database (https://string-db.org/), which systematically collects and integrates protein-protein interactions as well as functional associations (Szklarczyk et al. 2023).

### Ecological Niche Modelling

Ecological niche modelling (ENM) for *Stercorarius* spp. in the Southern Hemisphere were generated with respect to the habitat suitability of its reproductive zones. The results were projected under 4 climate change scenarios, comparing the areas of future niche potential expansion, stability, and contraction through a binarization of the model outputs. First, we constructed a spatial occurrence dataset for the 3 skua species (Chilean Skua, Brown Skua and South Polar Skua), with subsetting of the admixture and not admixed populations based on the results from the interspecific ADMIXTURE analysis (Fig. 1). Consequently, Falkland Skua occurrences were added to the Chilean Skua dataset, and Tristan Skua occurrences were added to the Brown Skua dataset. The occurrences were obtained from the Global Biodiversity Information Facility (GBIF 2023; www.gbif.org) and cleaned by filtering records outside a 200 km range around the breeding area, defined by the Birds of the World database (https://birdsoftheworld.org/). This step enabled us to remove taxonomically and geographically dubious records as well as non-breeding occurrences in other areas of the globe linked to feeding migration movements.

The final dataset consisted of 10,285 cleaned records, representing altogether the 3 skua species within the admixed and not admixed populations (supplementary fig. S30, Supplementary Material online). We used the blockCV R package (version 3.1-3; Valavi et al. 2019) to partition records into spatial blocks. In this way, the modeling incorporates the spatial and environmental structure of the data, which further reduces autocorrelation in species occurrence datasets and biases, as this is known to improve the predictive power of distribution models. The spatial blocks were then incorporated into the spatialMaxent software (version 3.4.4; Bald et al. 2023). This software is built around the maximum entropy modelling algorithm, while also implementing cross-validation of observations and generating results that minimize overfitting of spatially and environmentally biased observations. A model of the present potential niche was generated for each species and the admixed individuals using a set of 4 oceanic environmental variables obtained from BioOracle (version 2.2; Assis et al. 2018): chlorophyll, ice thickness, salinity, and sea surface temperature. The potential niche models constructed for the present period were then projected to the years 2050 and 2100, under low (RCP 2.6) and high (RCP 8.5) emission scenarios. The present and future projections obtained from spatialMaxent were binarized using the maximum specificity plus the sensitivity threshold. This threshold is meant to reduce the rates of omission (false negative) and commission (false positive) to the minimum, thus maximizing predictive reliability (Liu et al. 2005). The final predictions for the different scenarios were visually set around a buffer area of 200 km from the coast and mapped using ArcMap software (version 10.8.1, 2020; Desktop 2020), to identify areas of future potential niche change (expansion, stability, and contraction) of the modeled hybridization and non-hybridization areas. The changes in range size between the present and future scenarios of the skuas depicting the change in occupancy areas were calculated with the biomod2 package (version 4.2-4; Thuiller et al. 2016).

## Supplementary Material

msaf053_Supplementary_Data

## Data Availability

The data underlying this article are available in GenBank database (raw fastq reads) and can be accessed with Biosamples SAMN38698868-SAMN38698944, SAMN43185484-SAMN43185490 and SAMN35654692- SAMN35654709. All code used for the genomic analyses are available on the first author's GitHub (https://github.com/JorqueraJ/skuas_genomics). A list of all software used for the different data analysis is available in supplementary table S28, Supplementary Material online. All other data needed are provided in either the main text or the Supplementary material.
