# Supplementary material for: Genomic Introgression and Adaptation of Southern Seabird Species Facilitate Recent Polar Colonization
Source: Mol Biol Evol. 2025 Mar 20;42(3):msaf053. doi: 10.1093/molbev/msaf053 (PMC11954569; doi:10.1093/molbev/msaf053)
Supplement: msaf053_Supplementary_Data [file msaf053_supplementary_data.zip › MBE_Jorquera_2024_Supplementary_21Nov.pdf]

Genomic introgression and adaptation of southern seabird species facilitate recent polar colonization

Supplementary Materials

Supplementary Text

1. DNA isolation and Whole Genome sequencing

Genomic DNA was isolated from tissue samples following the salt extraction protocol by Aljanabi & Martinez, 1997. The extraction buffer for each sample was prepared with 10 mM Tris-HCl pH 7.8, TNE 1X and 25% SDS instead of 10 mM Tris–HCl pH 8.0, 0.4 M NaCl, 2 mM EDTA pH 8.0 and 20% SDS. The samples were incubated at 56ºC for 12 hours with the extraction buffer and 10 µL of proteinase K, after which 600 µL of 10 M ammonium acetate was used to precipitate DNA instead of 6 M NaCl. After extraction, DNA was resuspended in nuclease-free H2O and stored at -20°C.

Samples were quantified on the Qubit® Fluorometer 3.0 using the Qubit® dsDNA Kit (Thermo Fisher Scientific) and visually controlled for degradation by standard 1% agarose gel electrophoresis. We selected samples showing no DNA degradation and at least 50 ng/µL concentration for the preparation of libraries with ~350 bp insertions using TruSeq DNA Nano High Throughput Library Prep Kits (Illumina, cat. 20015965) and Whole Genome Sequencing on the Illumina Novaseq 6000 with a paired-end read length of 150 bp on four lanes at Medgenome (Delaware, USA).

SNP filtering and Linkage disequilibrium (LD) pruning

The resulting merged VCF file derived from the razorbill dataset was first normalized using the BCFtools norm (Danecek et al., 2021) function and further filtered using VCFtools (v.0.1.14) (Danecek et al., 2011) with specific parameters to retain SNPs with a minimum depth of 3 (-min-meanDP 3) and a minimum quality score of 30 (-minQ 30), kept only biallelic loci (--min-alleles 2 --max-alleles 2), remove indels (-remove-indels), and tolerated 5% of missing data (-max-missing 0.95). To remove loci in high linkage disequilibrium (LD), the VCF was filters for minor allele frequency (maf) > 0.05 and pruned using PLINK2 (version 2.00a2.3)(Purcell et al., 2007), filtering for squared correlation coefficient greater than 0.1 in a sliding window of 50 SNPs, shifted and recalculated every 5 SNPs. The unlinked autosomal SNPs set obtained, was used for PCA, Admixture, SNPdensity, average heterozygosity and private alleles analysis. A second LD-filtered VCF with no missing data was generated for the TreeMix analysis, and a filter for minor allele count > 2 (–mac 2) was applied instead of the filter for biallelic loci. After pruning, this VCF was converted into allele frequency input file for TreeMix using the vcf2treemix.sh script (https://github.com/speciationgenomics/scripts/blob/master/vcf2treemix.sh).

To retain as many sites as possible for positive selection and adaptive introgression analyzes performed using Dsuite (Malinsky et al., 2021) and RAiSD (Alachiotis & Pavlidis, 2018) respectively, the resulting merged VCF file derived from the kittiwake dataset was

normalized and only filtered for a minimum depth of 3 (–min-meanDP 3) and a minimum quality score of 30 (–minQ 30).

Supplementary Figures

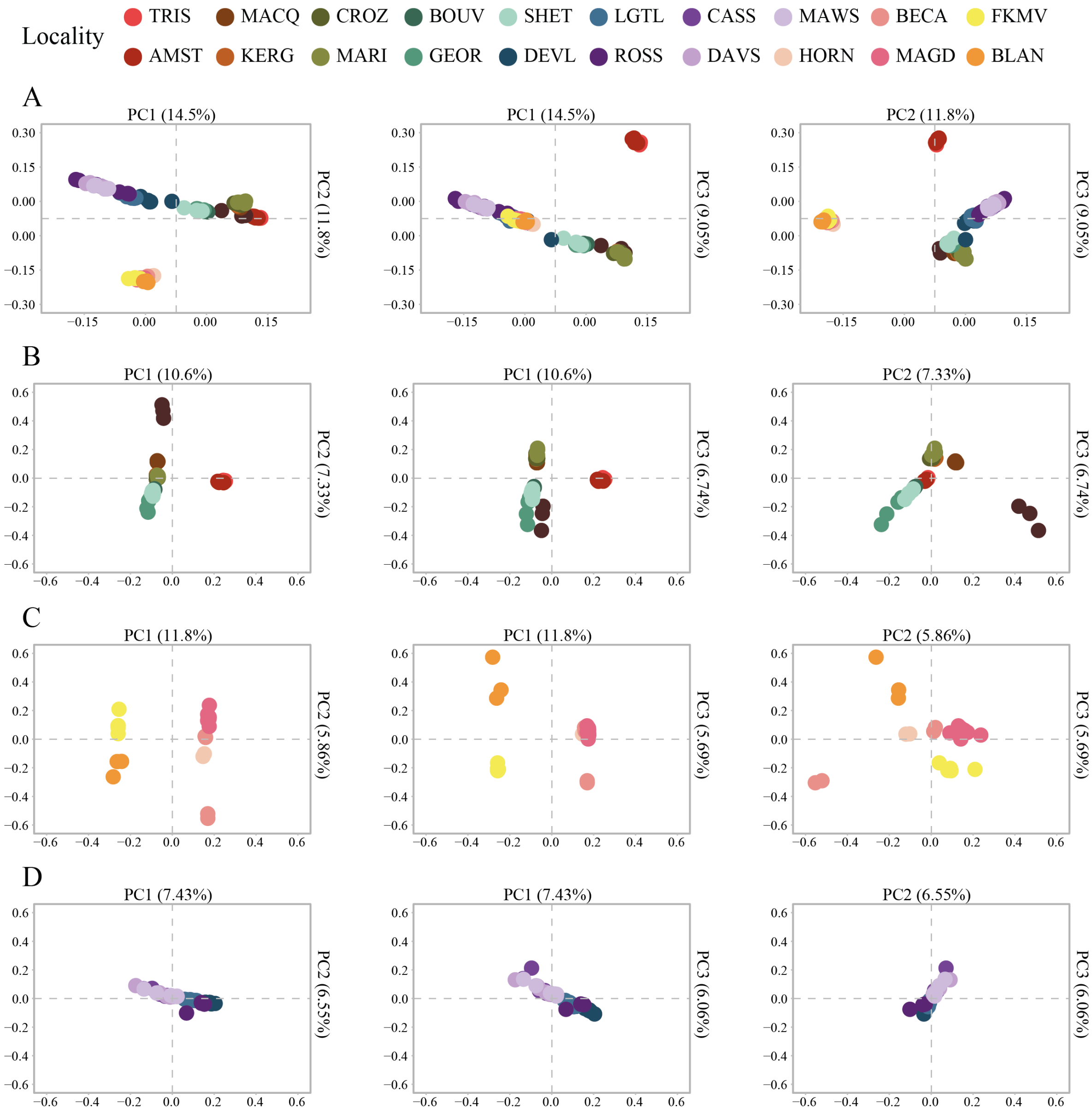

Fig. S1. Principal Component Analysis (PCA). PCA results which include graphs of PC2 vs PC1, PC2 vs PC3, PC3 vs PC1 inferred from different data sets of unlinked autosomal SNPs with 0% missing data (A) for 111 samples from 21 localities, (B) Brown skuas, (C) Chilean skuas and (C) South Polar skuas. Percentage of variation explained by each PC axis is given within parenthesis.

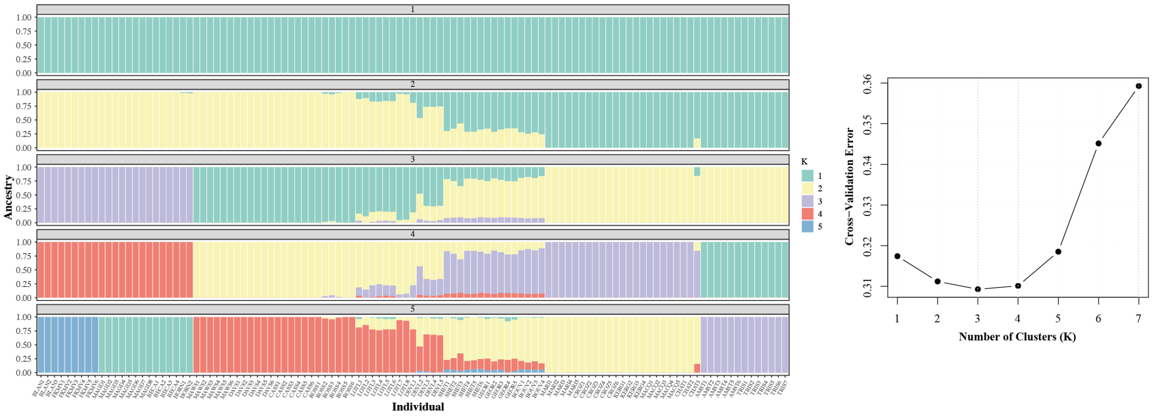

Figure. S2. Population structure inferred from ADMIXTURE analysis among all individuals. Each sample is denoted by a single vertical bar partitioned into K colors according to its proportion of ancestry in each of the clusters. Ancestral contributions are plotted for K = 1 to K = 7. According to the cross-validation errors, 3 was the optimum number of clusters.

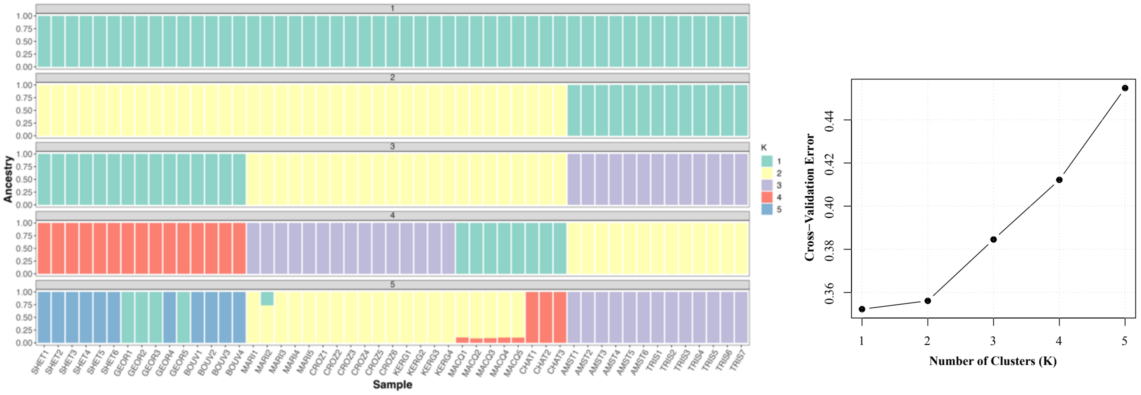

Fig. S3. Population structure inferred from ADMIXTURE analysis for the Brown skua individuals. Each sample is denoted by a single vertical bar partitioned into K colors according to its proportion of ancestry in each of the clusters. Ancestral contributions are plotted for K = 1 to K = 9. According to the cross-validation errors, 1 was the optimum number of clusters.

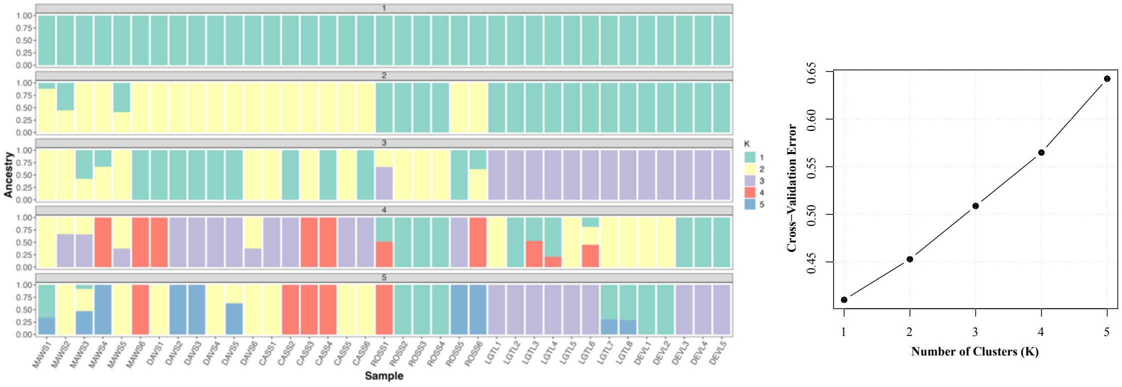

Fig. S4. Population structure inferred from ADMIXTURE analysis for the South Polar skua individuals. Each sample is denoted by a single vertical bar partitioned into K colors according to its proportion of ancestry in each of the clusters. Ancestral contributions are plotted for K = 1 to K = 6. According to the cross-validation errors, 1 was the optimum number of clusters.

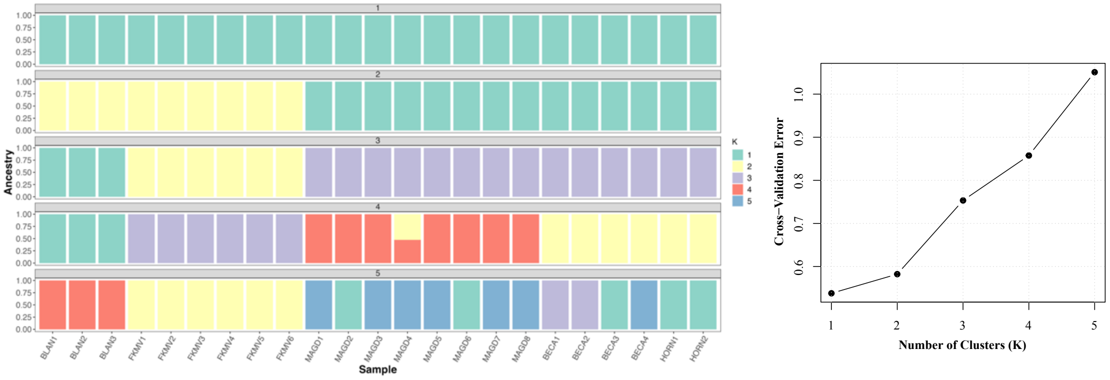

Fig. S5. Population structure inferred from ADMIXTURE analysis for the Chilean and Falkland skua individuals. Each sample is denoted by a single vertical bar partitioned into K colors according to its proportion of ancestry in each of the clusters. Ancestral contributions are plotted for K = 1 to K = 6. According to the cross-validation errors, 1 was the optimum number of clusters.

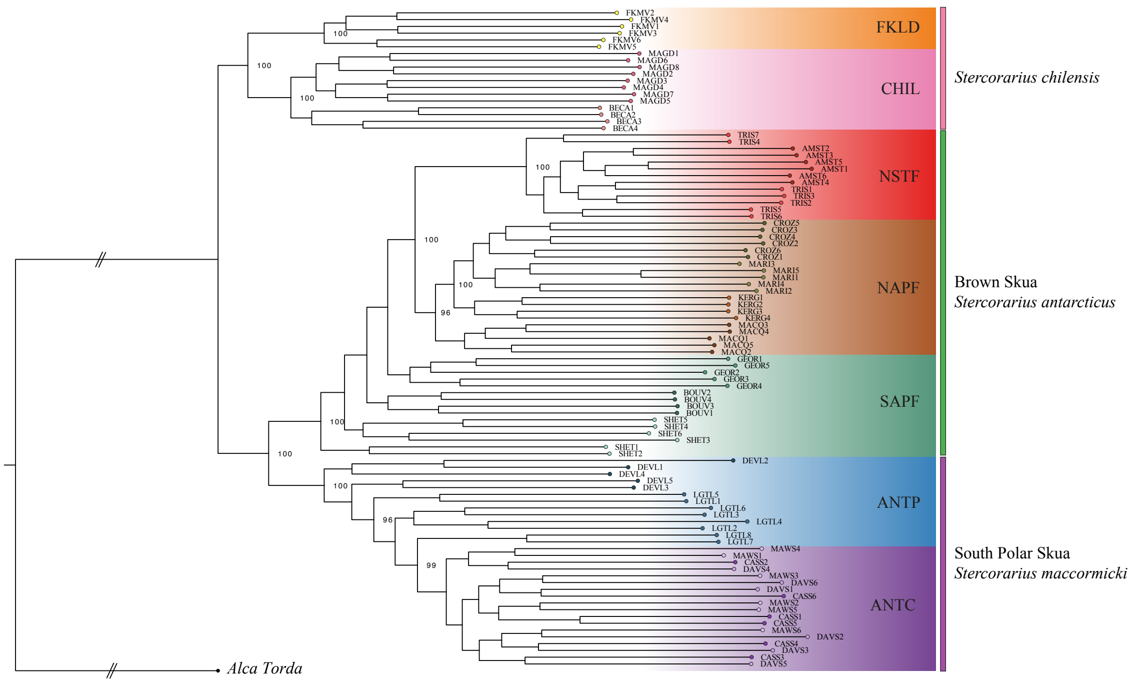

Fig. S6. Maximum likelihood phylogenetic tree of four skua taxa, using the razorbill (*Alca torda*) as outgroup for rooting. Constructed in RAxML using genome-wide single-nucleotide polymorphisms (SNPs), without considering the sex chromosomes. **CHIL:** Southern Chile (MAGD, BECA), **FKLD:** Southwest Atlantic shelves (FKMV), **ANTP:** Antarctic Peninsula (DEVL, LGTL), **ANTC:** Continental Antarctica (MAWS, CASS, DAVS), **SAPF:** South of the Antarctic Polar Front (BOUV, SHET, GEOR), **NAPF:** North of the Antarctic Polar Front (KERG, CROZ, MARI, MACQ), **NSTF:** North of the Subtropical Front (AMST,TRIS).

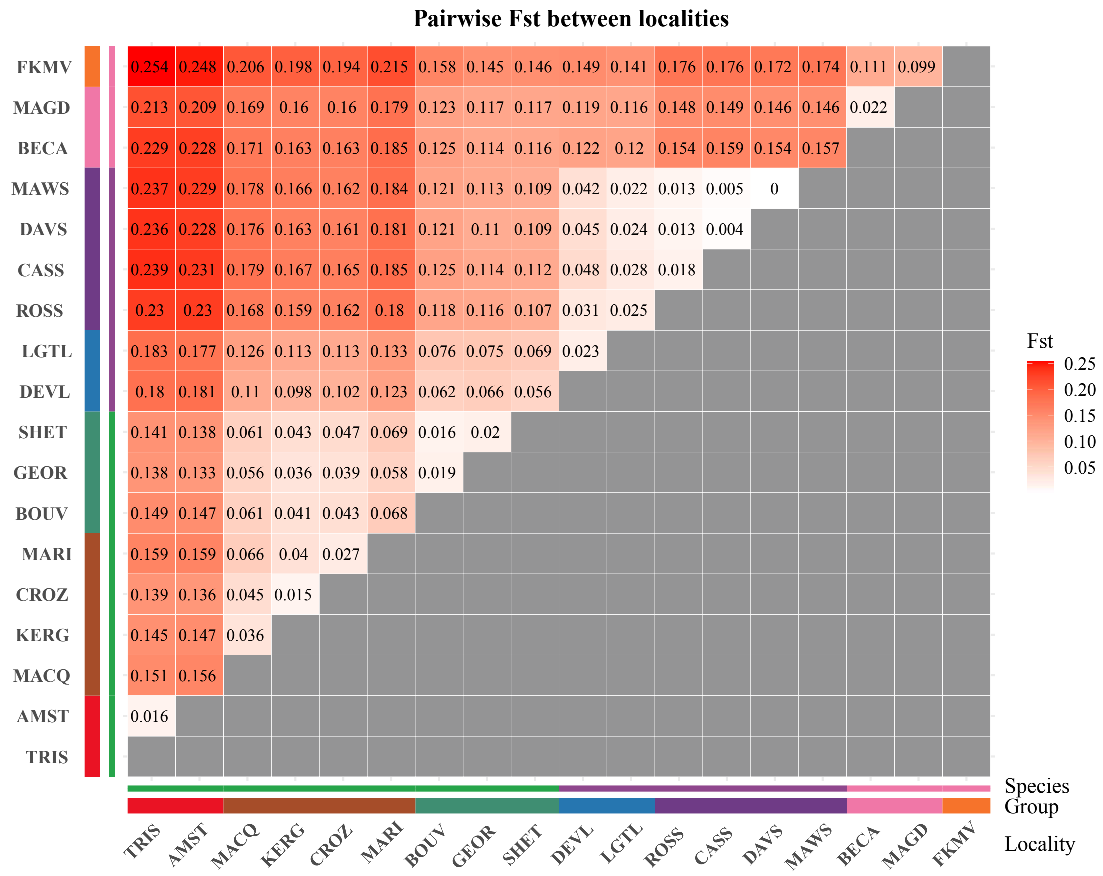

Fig. S7. Pairwise *Fst* values based on Weir and Cockerham's unbiased genetic distances (1984) for 17 localities. Darker red *Fst* values can be observed, indicating further greater divergence. The abbreviations correspond to the names of the localities in **Table S1**. Parameters were assessed genome-wide in 50kb sliding windows (25kb slide). **Note:** The localities BLAN, HORN, and CHAT were excluded from this analysis due to a low number of individuals.

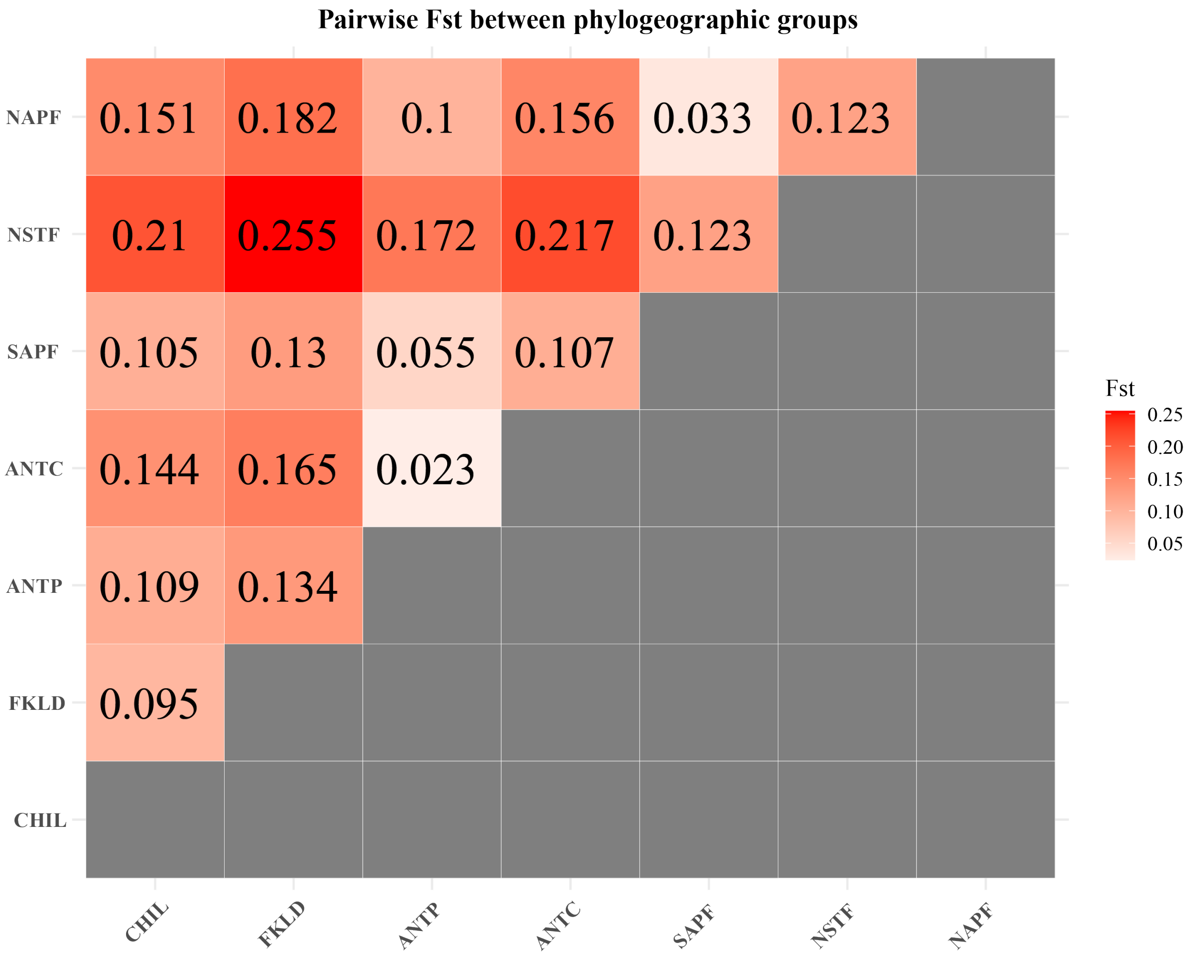

Fig. S8. Pairwise Fst values based on Weir and Cockerham's unbiased genetic distances (1984) for 7 phylogeographic groups. Darker red Fst values can be observed, indicating further greater divergence. The abbreviations correspond to the phylogeographic groups defined in the main text. Parameters were assessed genome-wide in 50kb sliding windows (25kb slide). **Note:** The localities BLAN, HORN, and CHAT were excluded from this analysis due to a low number of individuals.

A

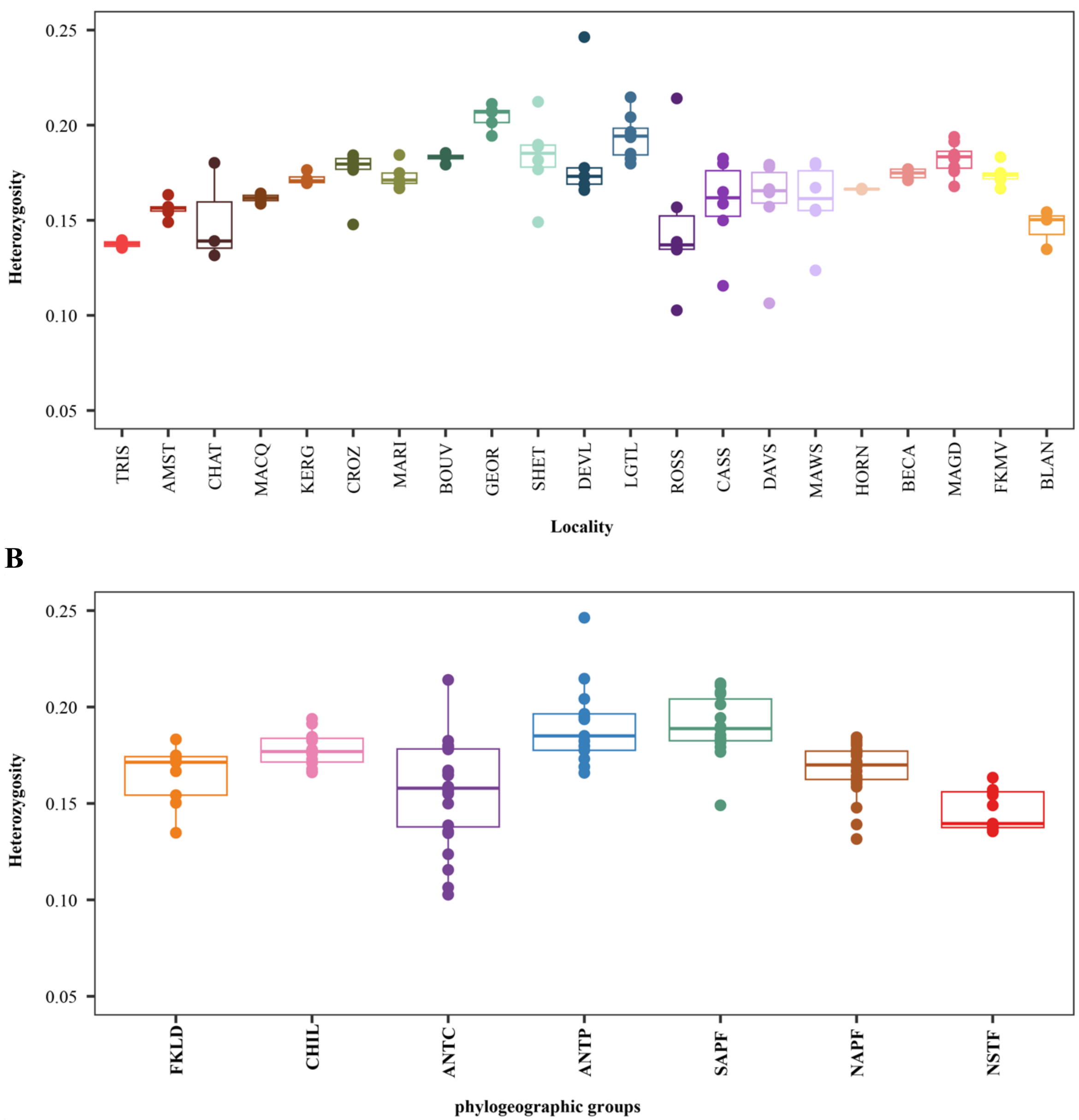

Fig. S9. Average local heterozygosity of skua across all samples (A) and genetic groups (B).

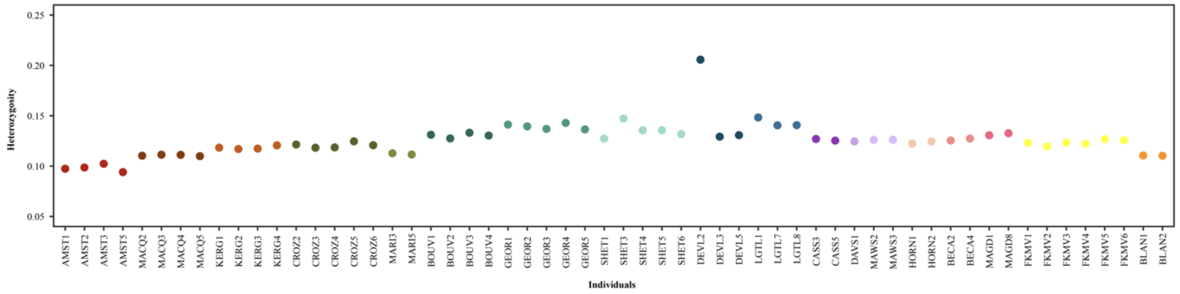

Fig. S10. Genome-wide heterozygosity of 58 skua individuals calculated using only high coverage sequencing data (14-15X).

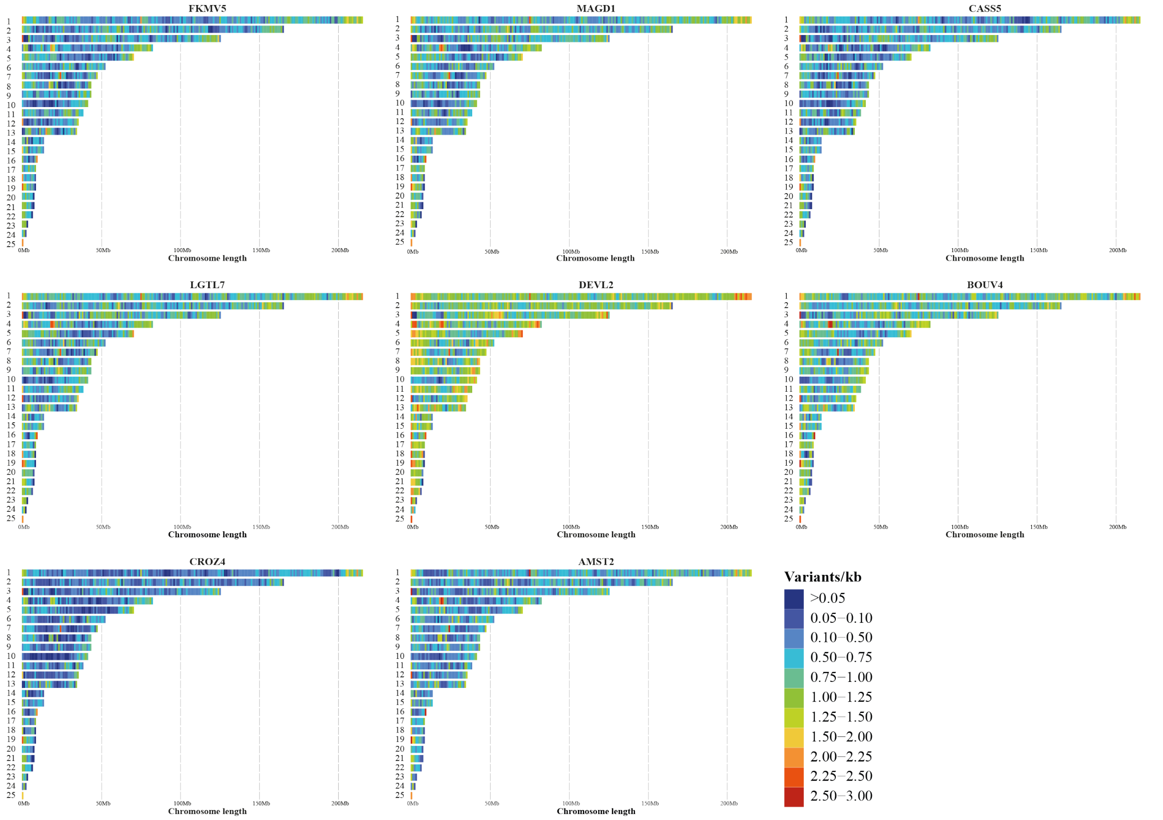

Fig. S11. The autosomal SNP density plots. Top are the SNP density plot from different locations of Chilean skua, followed by South Polar skua and Brown skuas.

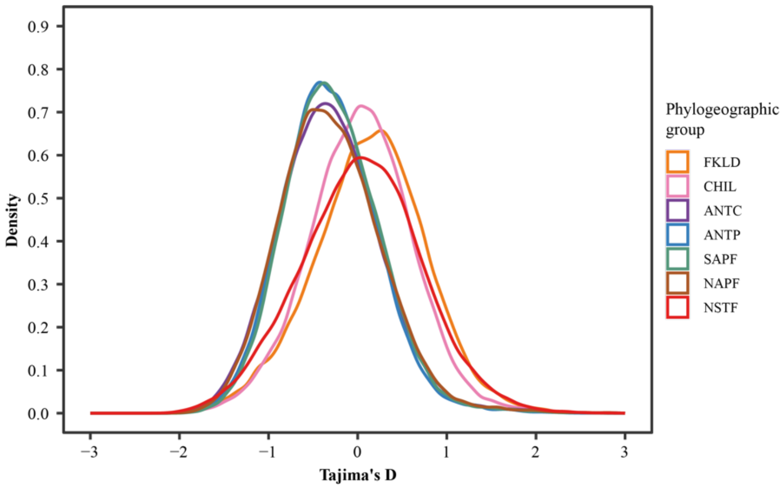

Fig. S12. Genome-wide Tajima’s D for the geographic groups for the 7 phylogeographic groups.

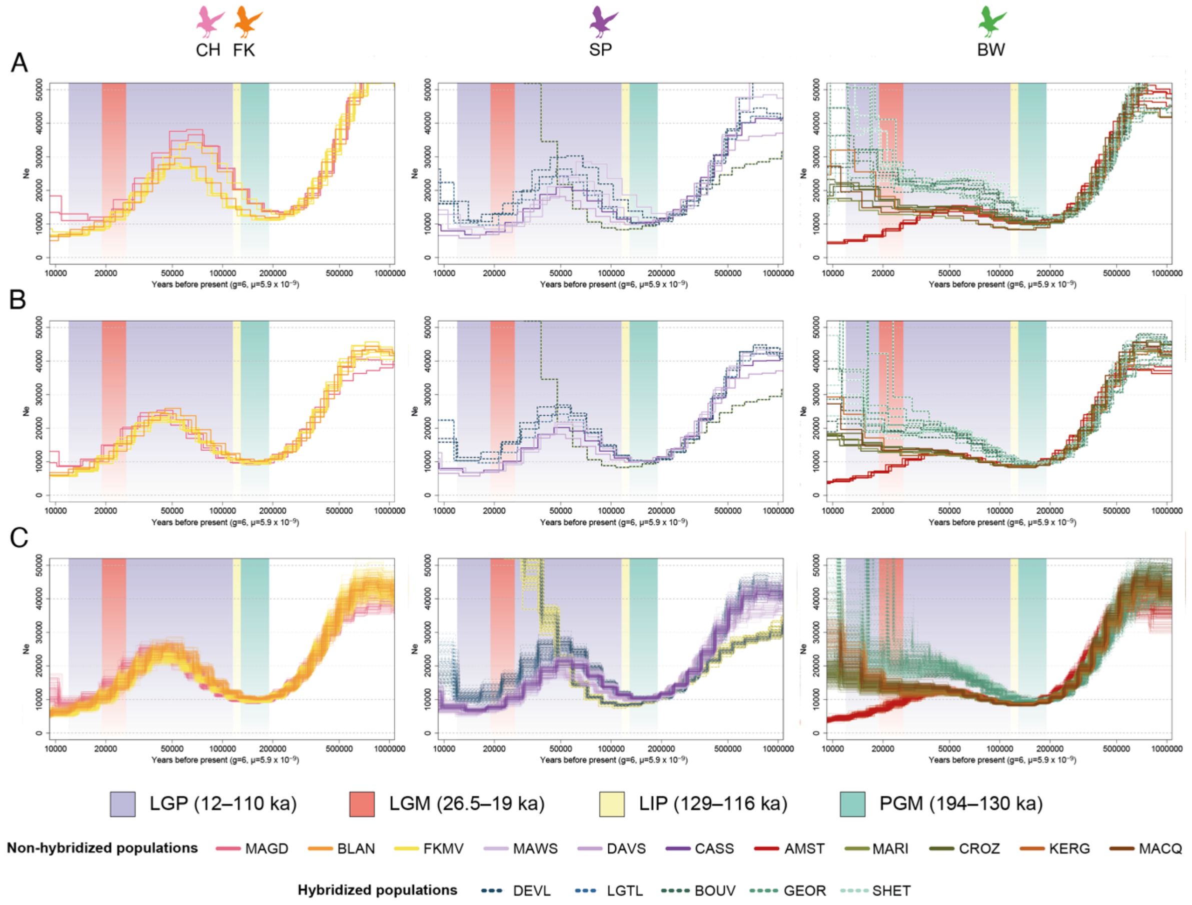

Figure S13. Influence of differences in genome coverage on the estimation of past effective population size (Ne) by PSMC analysis. (A) individuals of different sequence coverage (~8-30X) and (B) similar sequence coverage (~14-15X). The coloured lines represent the demographic histories of representative individuals of each genetic group observed in the intraspecific PCA analyses. (C) Bootstrap replicates for each individual. The dashed lines represent individuals from admixed populations as revealed by the intraspecific ADMIXTURE. Vertical shaded areas represent key glaciation: Penultimate Glacial Maximum (**PGM**, 194–130 ka), Last Interglacial Period (**LIG**, 129–116 ka), Last Glacial Period (**LGP**, 12–110 ka), Last Glacial Maximum (**LGM**, 26.5–19 ka). The plots were scaled using a mutation rate (μ) of 5.9×10^{-9} mutations per site per generation and a generation time of 6 years.

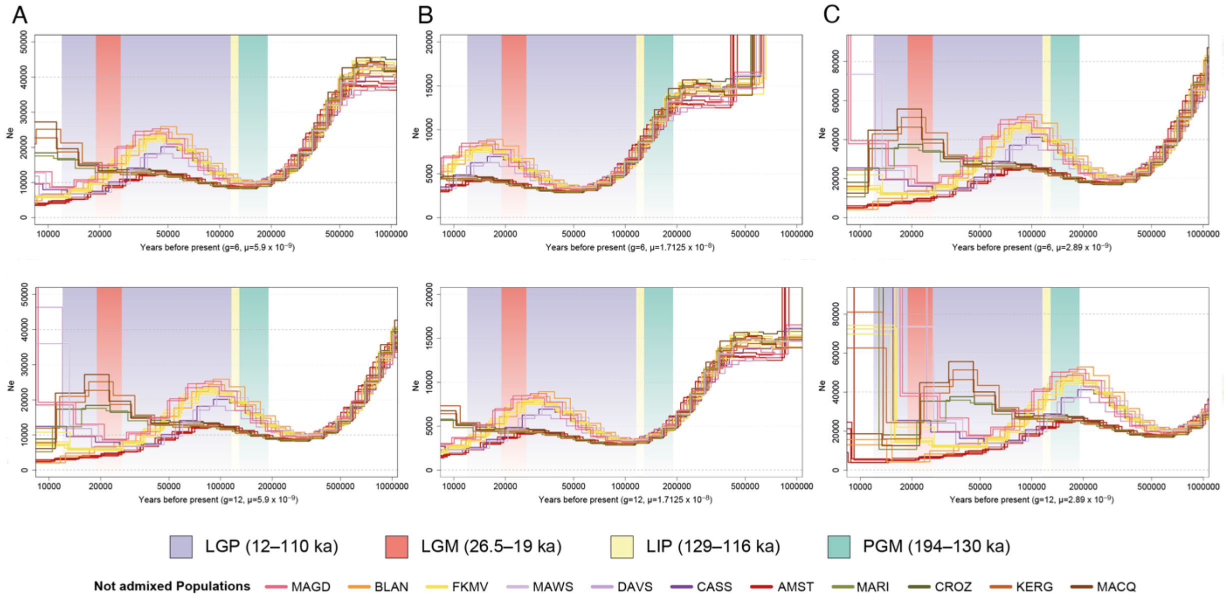

Figure S14. Influence of differences in mutation rates and generation times on the estimation of past effective population size (Ne) by PSMC analysis. The coloured lines represent the demographic histories of representative individuals of each genetic group observed in the intraspecific PCA analyses. For better visualization, only individuals of similar sequence coverage (~14-15X) representative of not admixed populations were included. The mutation rates (μ) for three closely related avian species were compared: (A) southern giant petrel ($\mu = 5.9 \times 10^{-9}$), (B) Atlantic puffin ($\mu = 1{,}7125 \times 10^{-9}$ and (C) northern fulmar ($\mu = 2.89 \times 10^{-9}$). The top plots were scaled using a generation time of 6 years, while bottom plots were scaled using a generation time of 12 years. Vertical shaded areas represent key glaciation: Penultimate Glacial Maximum (**PGM**, 194–130 ka), Last Interglacial Period (**LIG**, 129–116 ka), Last Glacial Period (**LGP**, 12–110 ka), Last Glacial Maximum (**LGM**, 26.5–19 ka). The plots were scaled using a mutation rate (μ) of 5.9×10^{-9} mutations per site per generation and a generation time of 6 years.

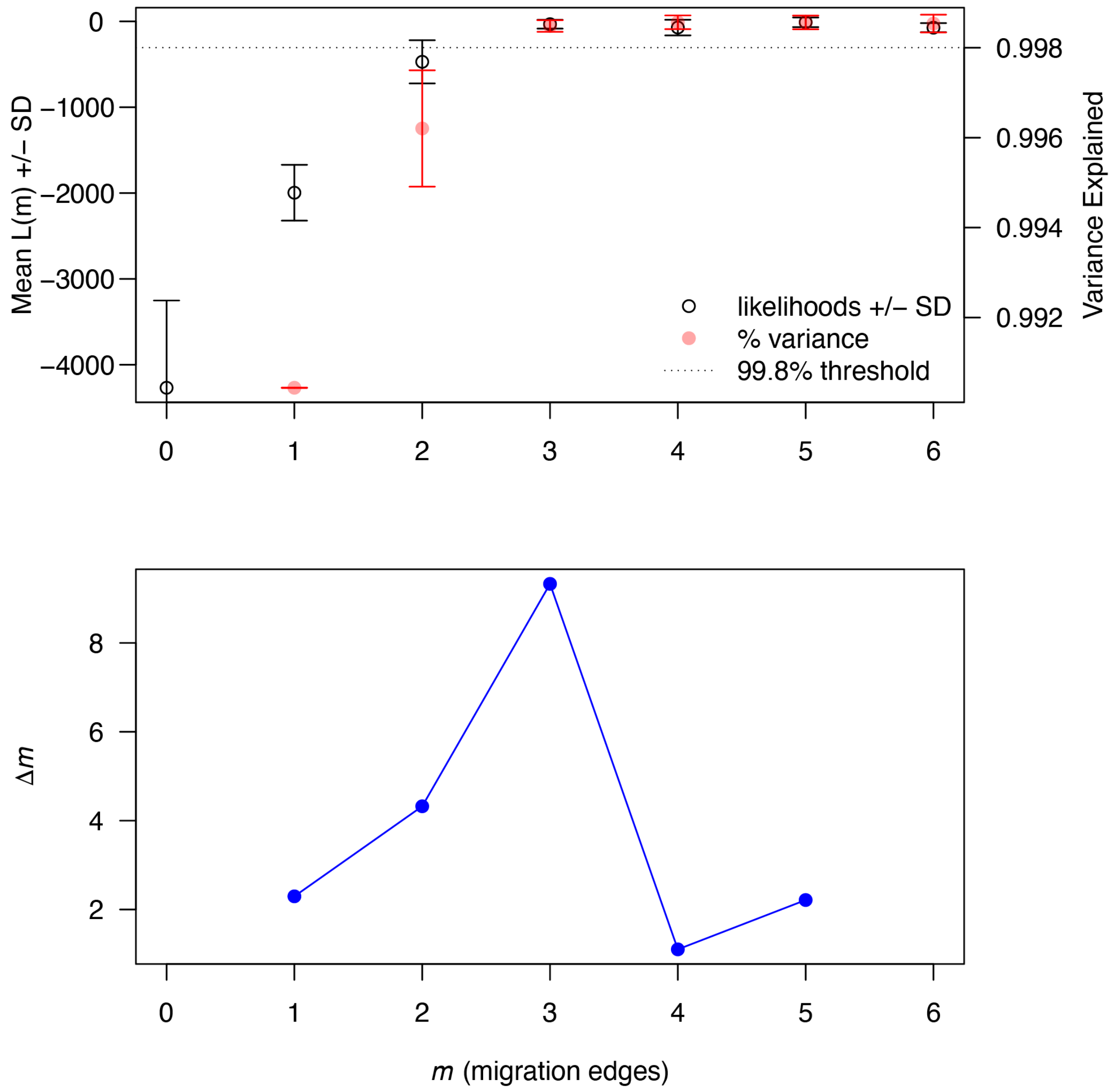

Fig. S15. The mean and standard deviation (SD) and proportion of variance explained obtained for Treemix. The mean and standard deviation (SD) across 10 iterations for the composite likelihood L(m) (left axis, black circles) and proportion of variance explained (right axis, red circles) (top); and the second-order rate of change (Δm) across values of m (bottom). Both figures are the output produced by the R package 'OptM'.

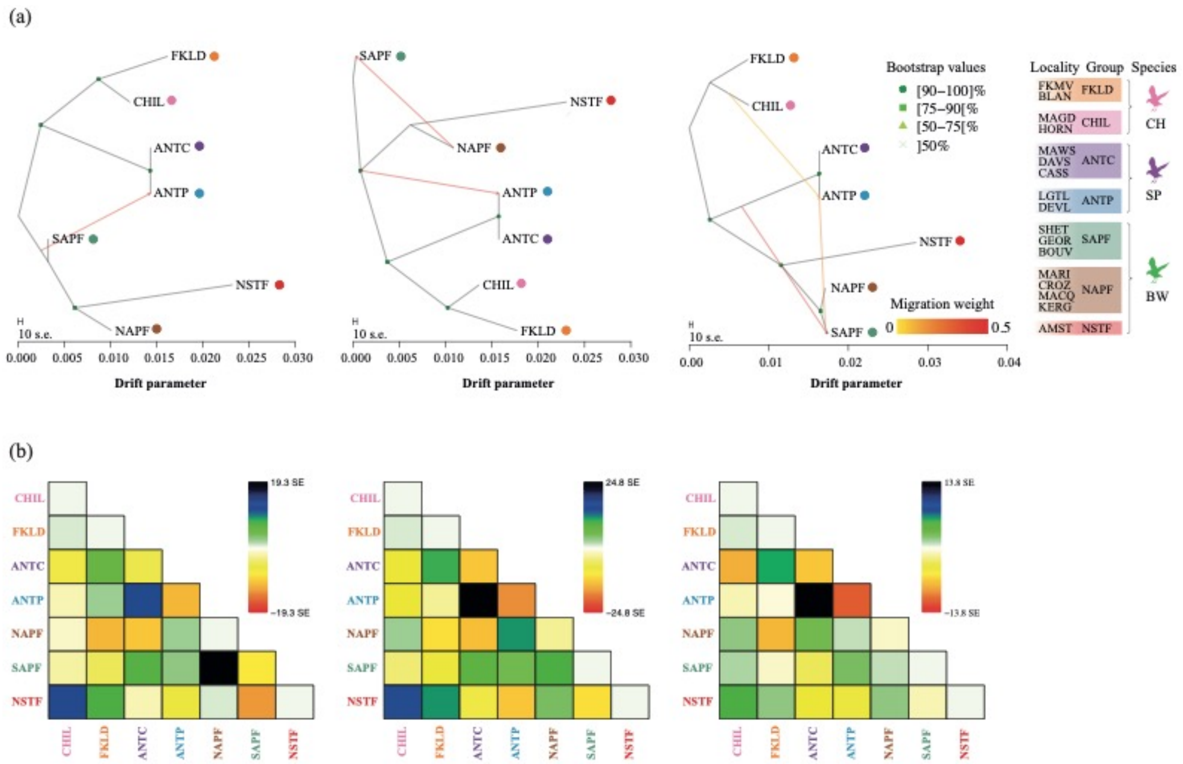

Fig. S16. TreeMix unrooted maximum likelihood tree plots of skua populations and their corresponding residual fit. Positive residuals (blue/black colours) indicate populations pairs that are more closely related to each other than suggested by the tree and may indicate potential admixture events. Migration arrows are colored according to their weight based on the proportion of alleles in the descendent population that are derived from the ancestral population.

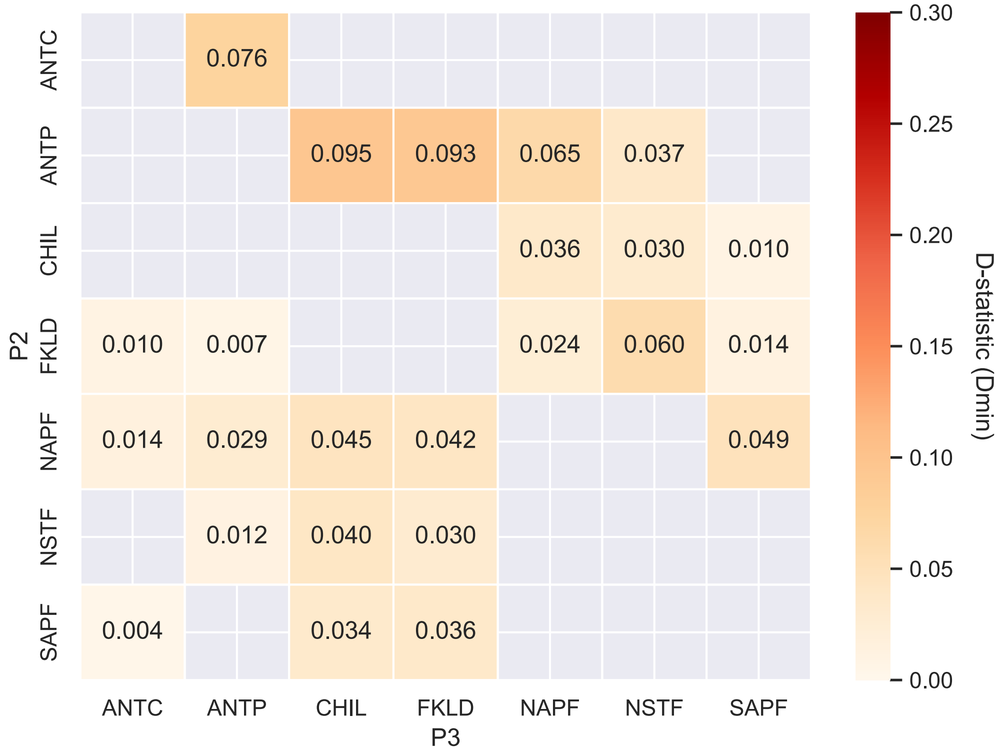

P2
ANTC
ANTP
CHIL
FKLD
NAPF
NSTF
SAPF
0.076
0.095
0.093
0.065
0.037
0.036
0.030
0.010
0.010
0.007
0.024
0.060
0.014
0.014
0.029
0.045
0.042
0.049
0.012
0.040
0.030
0.004
0.034
0.036
ANTC
ANTP
CHIL
FKLD
NAPF
NSTF
SAPF
P3
0.30
0.25
0.20
0.15
0.10
0.05
0.00
D-statistic (Dmin)

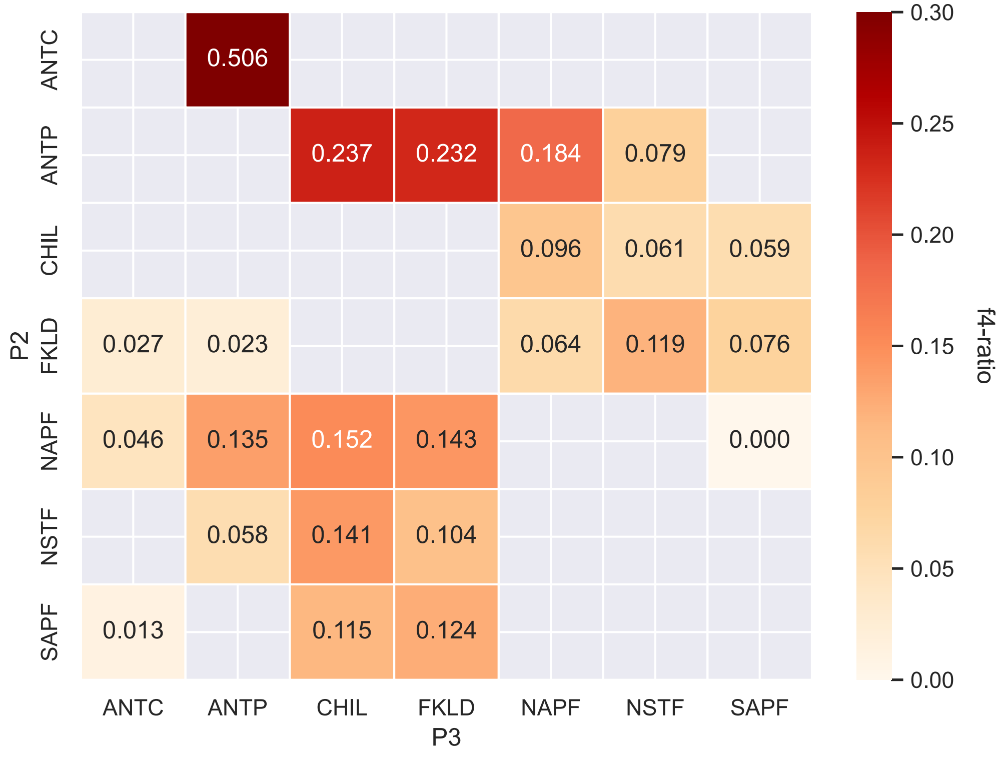

P2
ANTC
ANTP
CHIL
FKLD
NAPF
NSTF
SAPF
0.506
0.237
0.232
0.184
0.079
0.096
0.061
0.059
0.027
0.023
0.064
0.119
0.076
0.046
0.135
0.152
0.143
0.000
0.058
0.141
0.104
0.013
0.115
0.124
ANTC
ANTP
CHIL
FKLD
NAPF
NSTF
SAPF
P3
0.30
0.25
0.20
0.15
0.10
0.05
0.00
f4-ratio

Fig S17. D statistic and the f4-ratio method. Darker colors in heat map represent increasing evidence of gene flow between lineages, and gray data points in the matrix correspond to tests that are not applicable to the provided phylogeny. The D statistic and f4-ratio reflect evidence of excess allele exchange between P3 and P2 for each trio. **CHIL:** Southern Chile (MAGD, BECA, HORN), **FKLD:** Southwest Atlantic shelves (FKMV, BLAN), **ANTP:** Antarctic Peninsula (DEVL, LGTL), **ANTC:** Continental Antarctica (MAWS, CASS, DAVS, ROSS), **SAPF:** South of the Antarctic Polar Front (BOUV, SHET, GEOR), **NAPF:** North of the Antarctic Polar Front (KERG, CROZ, MARI, MACQ), **NSTF:** North of the Subtropical Front (CHAT, AMST), **STF:** Subtropical Front, **AFP:** Antarctic Polar Front.

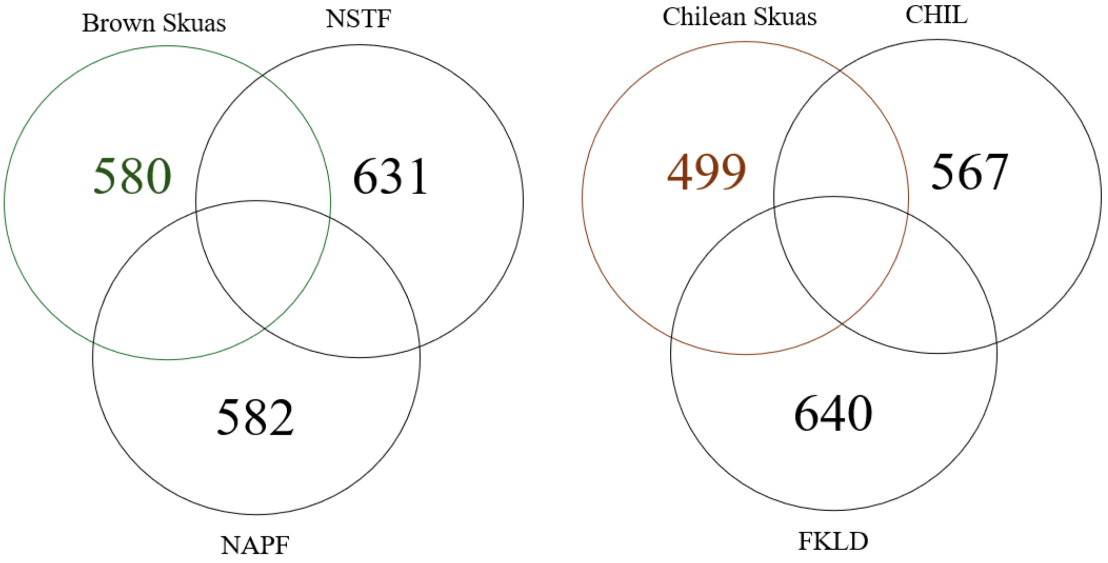

Fig S18. Comparison of unique and shared genes obtained with RAiSD when analyzing individuals as a species, as well as part of a genetic group. In this case, the comparison was made for Brown skua against NSTF, NAPF and Chilean skua against FKLD, CHIL.

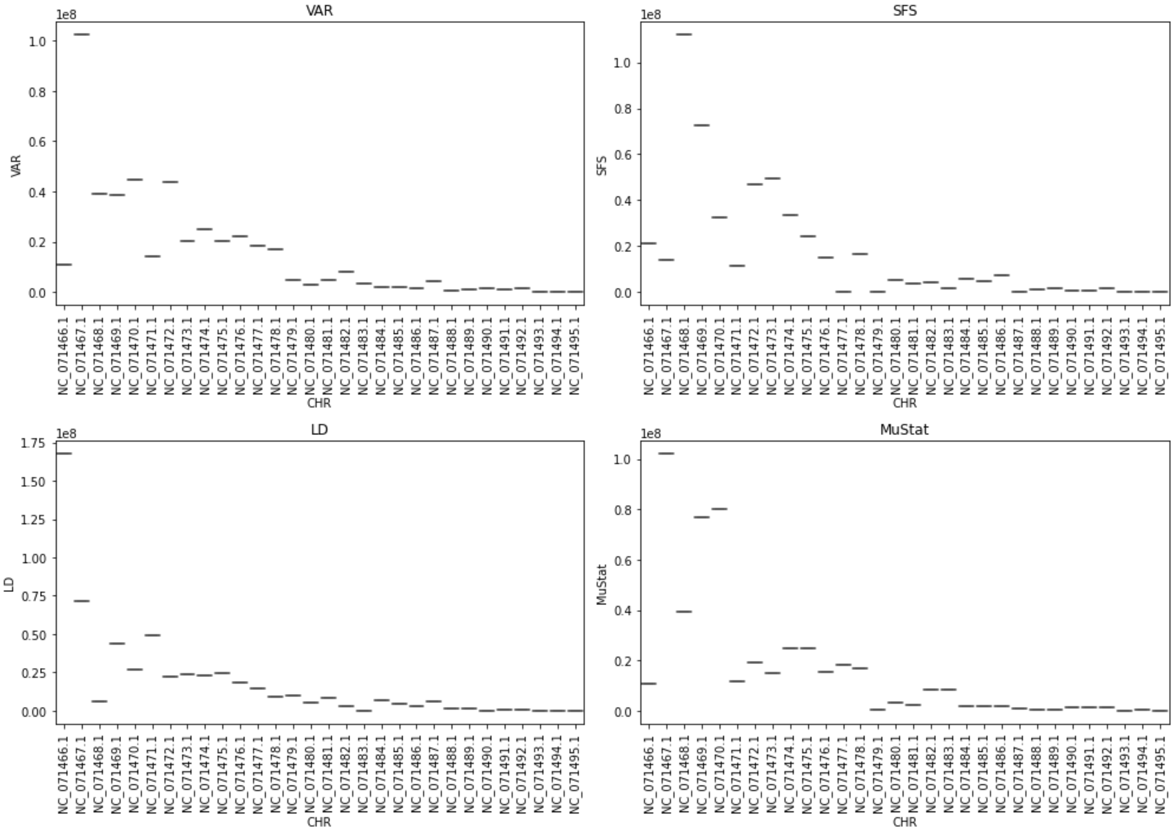

Fig. S19. Distribution of VAR, SFS, LD and MuStat values along different chromosomes (CHR) in the analysed genomes of South polar skuas. Each graph has the X-axis labeled with chromosomes from NC_071466.1 to NC_071495.1, while the Y-axis shows the corresponding values of each parameter. Some parameters, such as VAR and MuStat, show higher variability in the early chromosomes compared to the others.

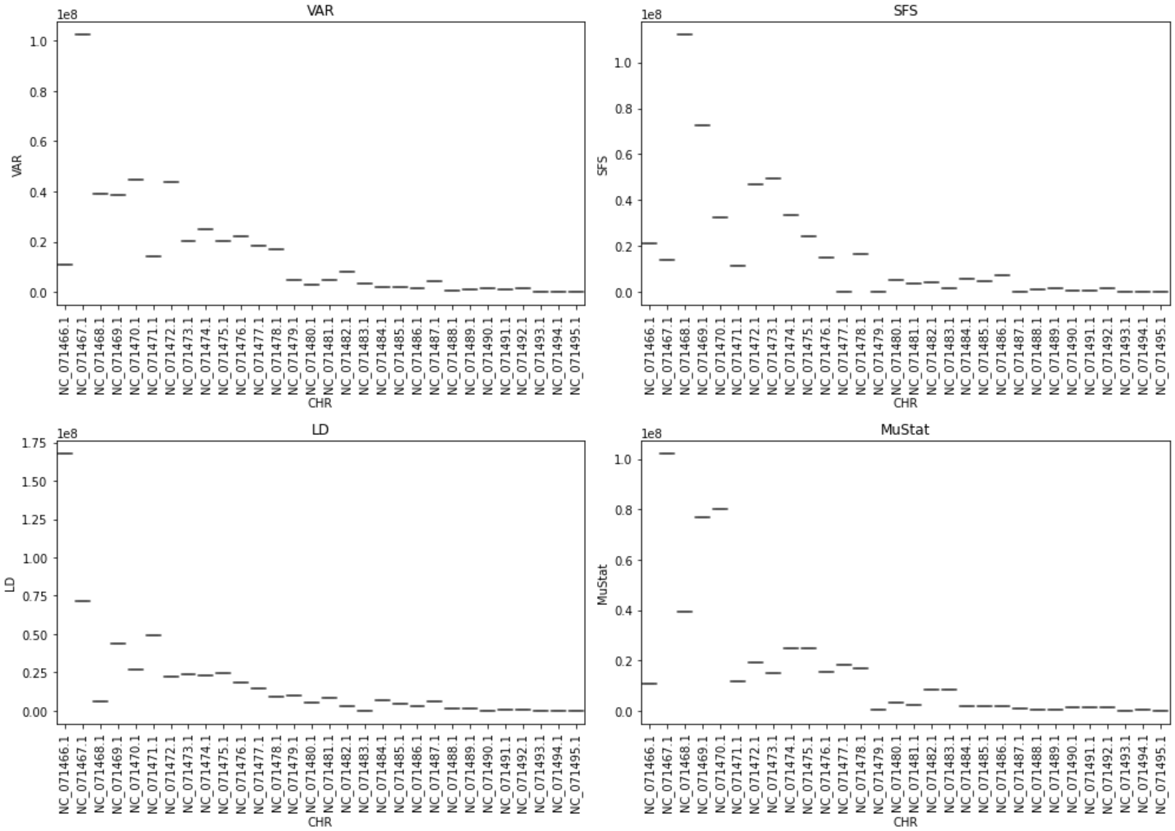

Fig. S20. Distribution of VAR, SFS, LD and MuStat values along different chromosomes (CHR) in the analysed genomes of Chilean skuas. Each graph has the X-axis labeled with chromosomes from NC_071466.1 to NC_071495.1, while the Y-axis shows the corresponding values of each parameter. Some parameters, such as VAR and MuStat, show higher variability in the early chromosomes compared to the others.

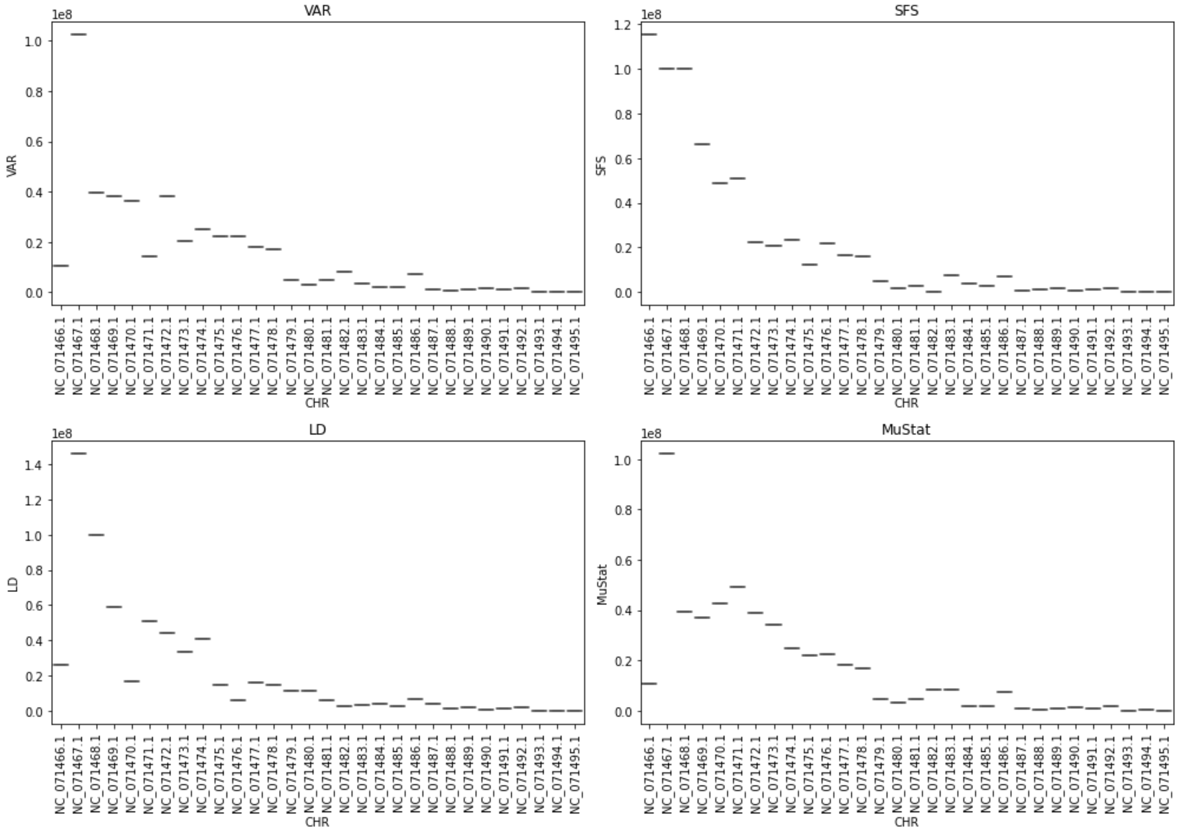

Fig. S21. Distribution of VAR, SFS, LD and MuStat values along different chromosomes (CHR) in the analyzed genomes of Brown skuas. Each graph has the X-axis labeled with chromosomes from NC_071466.1 to NC_071495.1, while the Y-axis shows the corresponding values of each parameter. Some parameters, such as VAR and MuStat, show higher variability in the early chromosomes compared to the others.

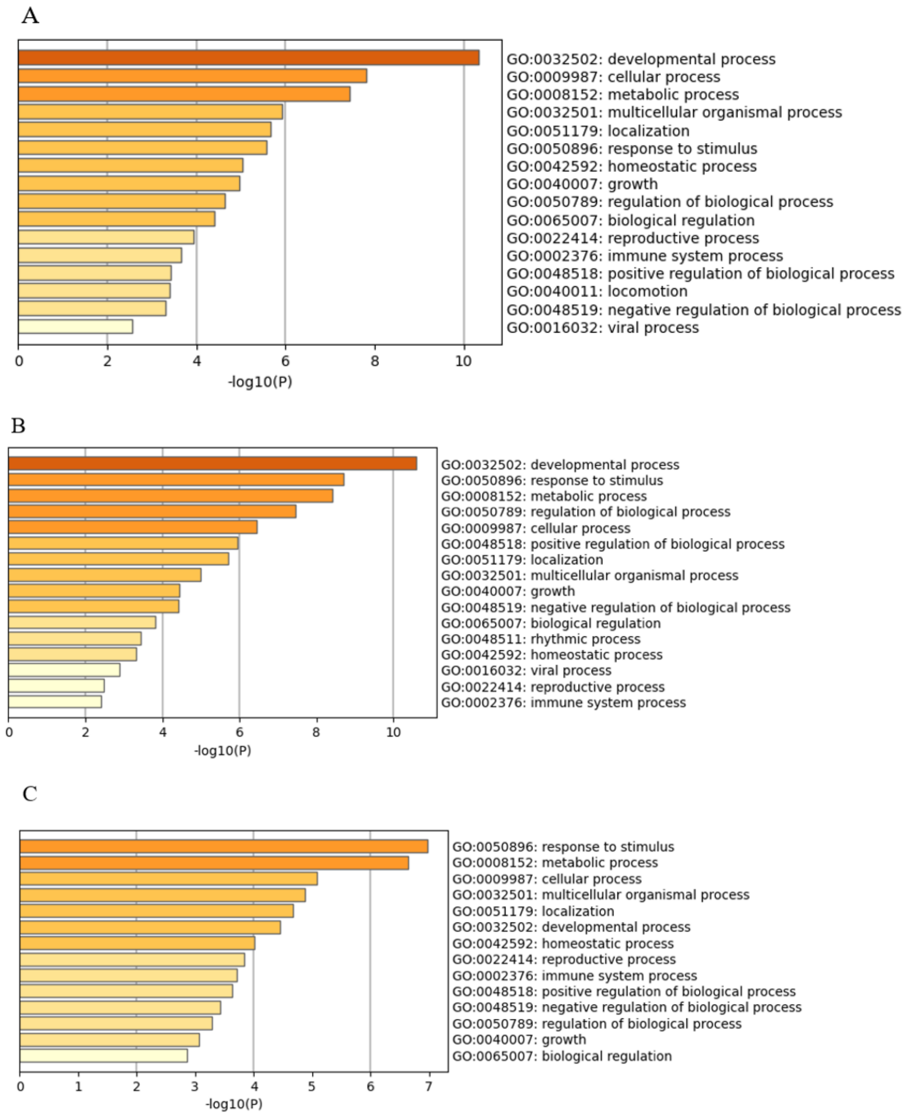

Fig. S22. GO term enrichment analysis of the genes under selection obtained for skua species, using RAiSD. Brown skuas (A), South Polar skuas (B) and Chilean skuas (C) with no admixed individuals. The bar graph presents the statistical significance of various biological processes, evaluated using p-value values by Metascape (Zhou et al., 2019). The results suggest the presence of key processes that could be regulating biological responses in the context studied.

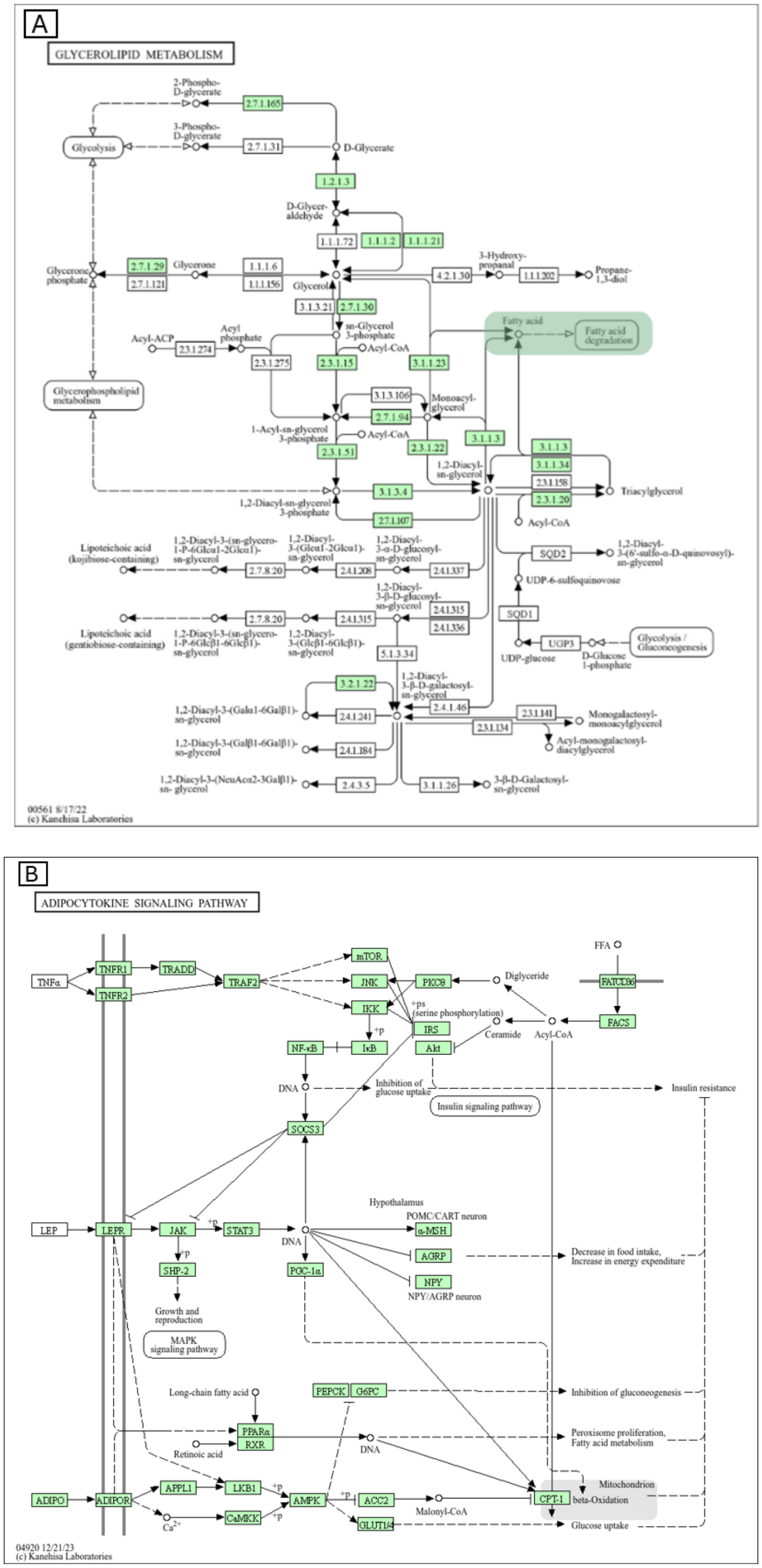

Fig. S23. KEGG Pathways of interacting genes for Brown skua and NAPF, NSTF in the context of lipid metabolism in *Gallus gallus* (chicken). (A) Description of the Glycerolipid metabolism process pathway (PATH: gga00561). (B) Peroxisome proliferator-

activated receptors (PPARs) are nuclear hormone receptors that are activated by fatty acids and their derivatives (PATH: gga03320).

A

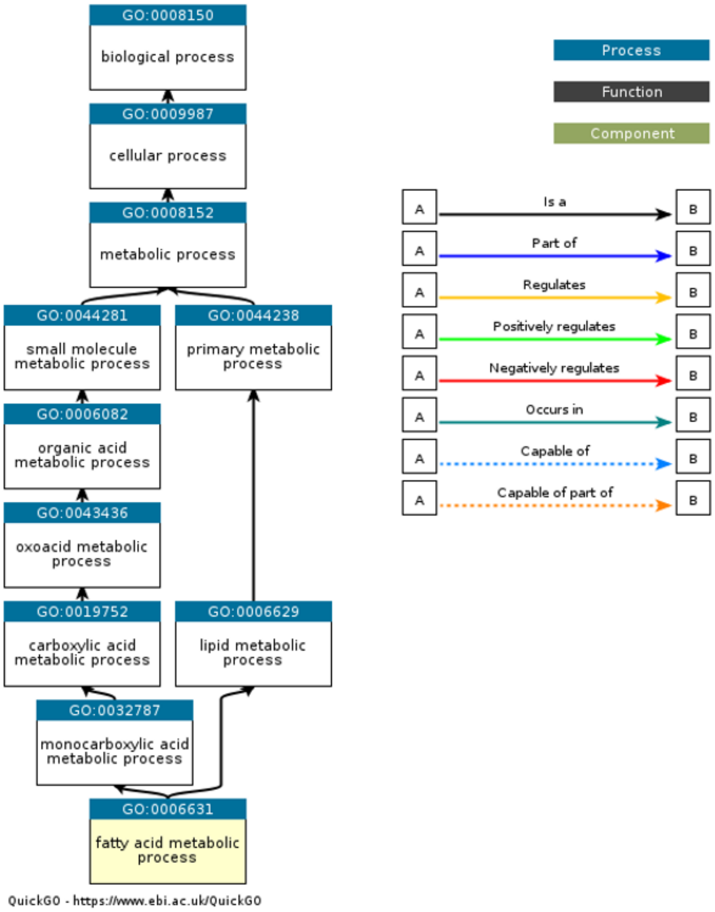

B

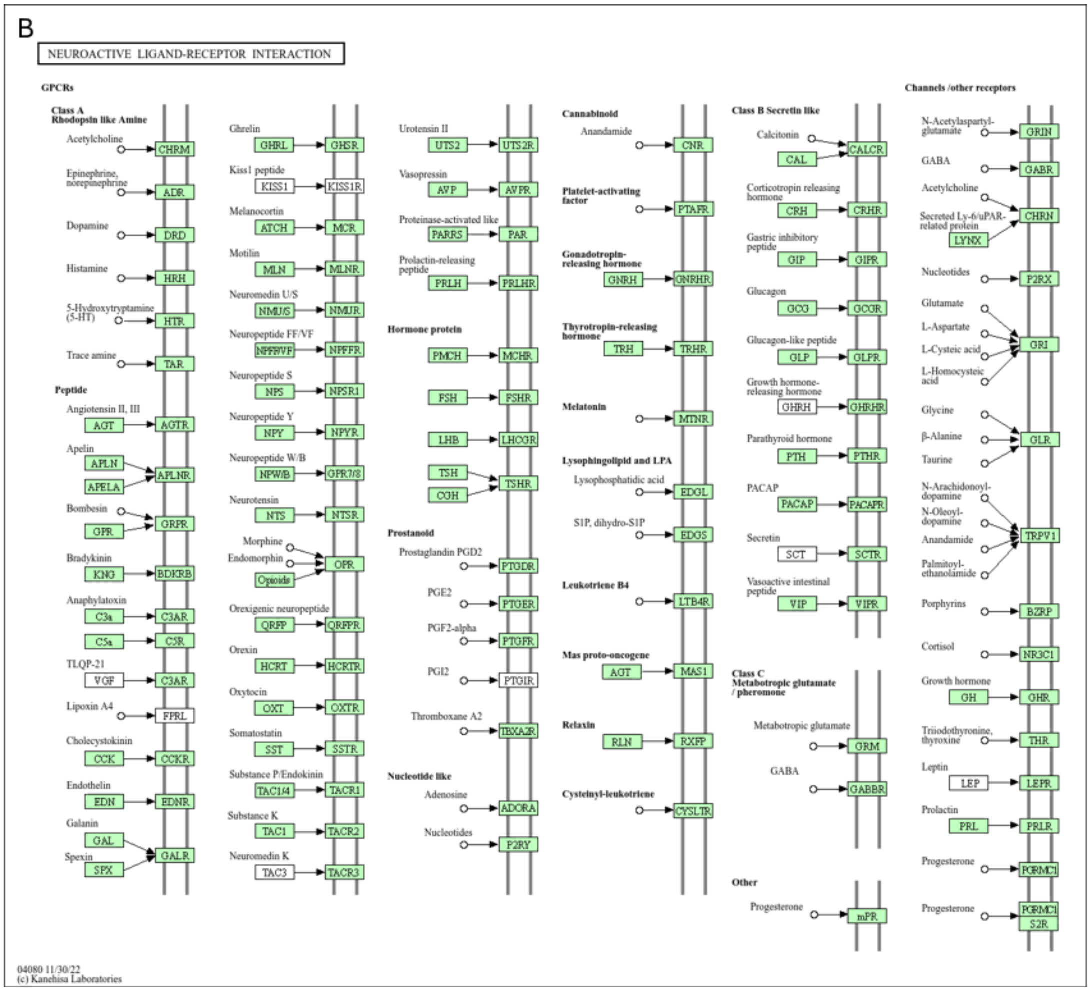
NEUROACTIVE LIGAND-RECEPTOR INTERACTION
GPCRs
Class A Rhodopsin like Amine
Peptide
Hormone protein
Prostanoid
Nucleotide like
Cannabinoid
Platelet-activating factor
Gonadotropin-releasing hormone
Thyrotropin-releasing hormone
Melatonin
Lysophospholipid and LPA
Leukotriene B4
Mas proto-oncogene
Relaxin
Cysteinyl-leukotriene
Class B Secretin like
Class C Metabotropic glutamate / pheromone
Other
Channels /other receptors
04080 11/30/22
(c) Kanehisa Laboratories

C

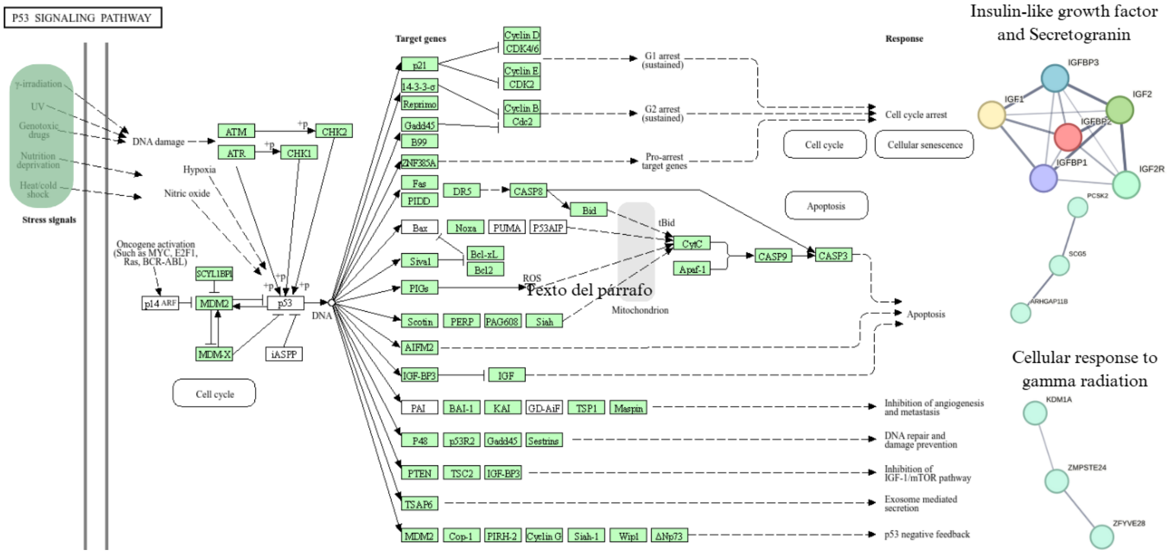
P53 SIGNALING PATHWAY
Stress signals
DNA damage
Target genes
Response
G1 arrest (sustained)
G2 arrest (sustained)
Pro-arrest target genes
Cell cycle arrest
Cell cycle
Cellular senescence
Apoptosis
Mitochondrion
Texto del párrafo
Inhibition of angiogenesis and metastasis
DNA repair and damage prevention
Inhibition of IGF-1/mTOR pathway
Exosome mediated secretion
p53 negative feedback
Insulin-like growth factor and Secretogranin
IGFBP3
IGF1
IGF2
IGFBP2
IGFBP1
IGF2R
Cellular response to gamma radiation
KDM1A
ZMPSTE24
ZFYVE28

D

Network STRING. Significant interaction between HOXC13, HOXC12, DNAJC24 genes

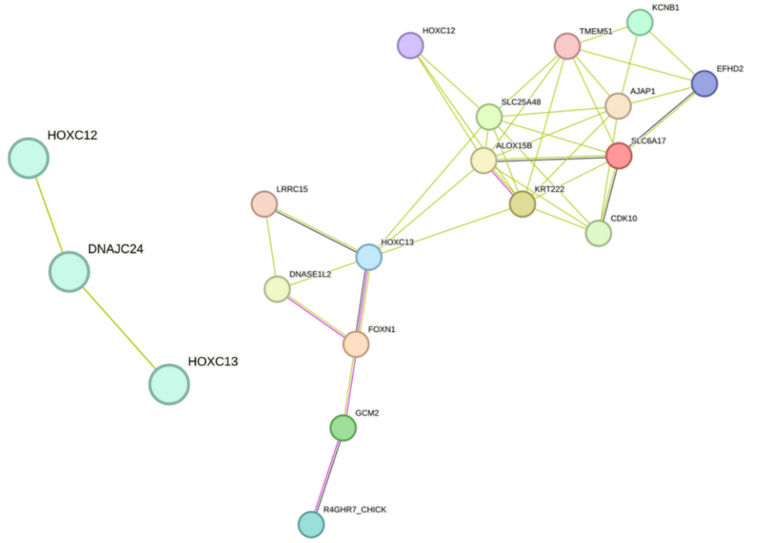

Fig. S24. STRING Interacting genes for South Polar skua and ANTC. (A) Biological process (QuickGO:Term GO:0006631) about the chemical reactions and pathways involving fatty acids, aliphatic monocarboxylic acids liberated from naturally occurring fats and oils by hydrolysis. (B) KEGG Pathways showing AMPA glutamate receptor complex, Neuroactive ligand-receptor interaction in *Gallus gallus* (chicken). (C) Enrichment about Secretogranin and insulin metabolism involved in lipid metabolism. (D) STRING interaction network showing significant interactions between the genes HOXC13, HOXC12 and DNAJC24, associated with the development of keratinized structures.

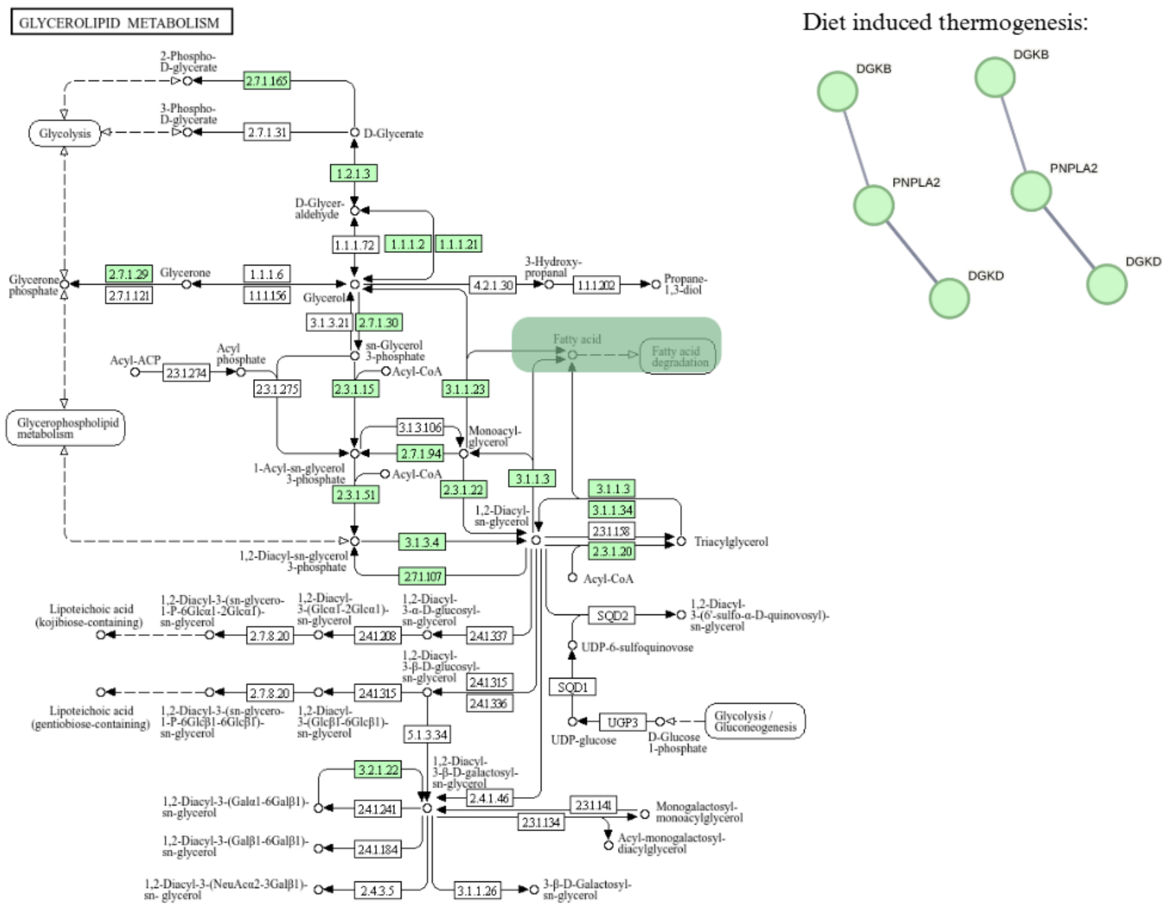

Fig. S25. STRING Interaction Network and KEGG Pathways in Chilean skuas. Diet-induced thermogenesis (STRING group, CL:21621), involving interaction significant between genes in cluster.

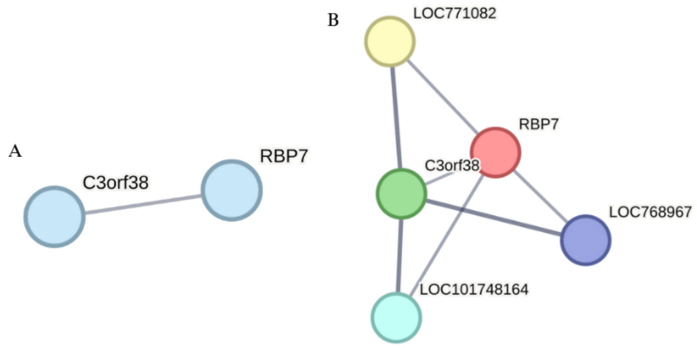

Fig. S26. STRING Interaction Network in Hyb-Br. (A) Significant interaction in fatty-acid metabolism with the genes RBP7 and C3orf38. (B) Cluster number CL:10516 of the NTF2-like domain superfamily and the lipocalin/cytosolic fatty-acid binding protein family

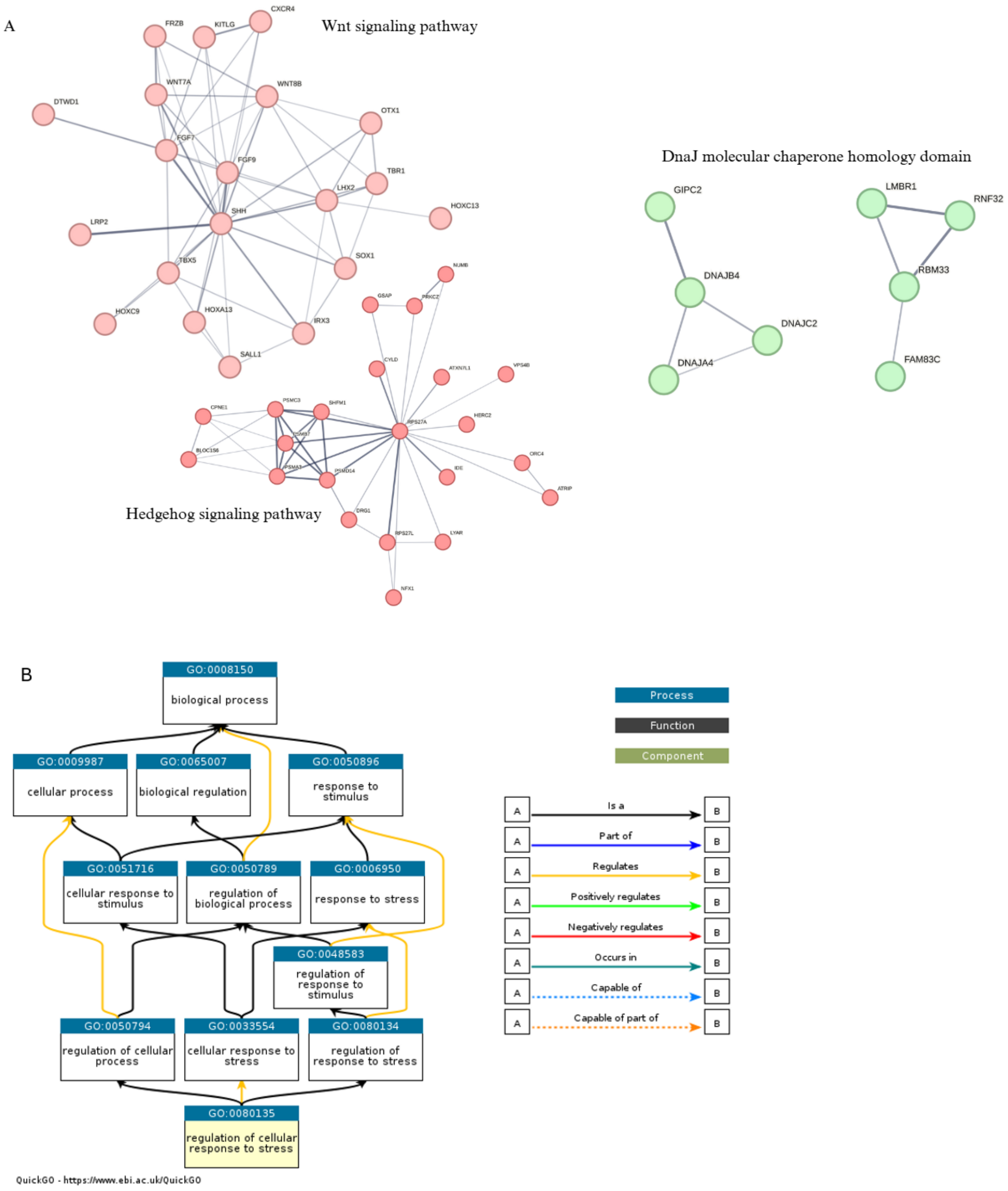
A
Wnt signaling pathway
CXCR4
FRZB
KITLG
WNT7A
WNT8B
DTWD1
OTX1
FGF7
FGF9
TBR1
LHX2
HOXC13
SHH
LRP2
SOX1
TBX5
NUMB
GSAP
PRKCZ
HOXC9
HOXA13
IRX3
SALL1
CYLD
ATXN7L1
VPS4B
CPNE1
PSMC3
SHFM1
RPS27A
HERC2
PSMB7
BLOC1S6
PSMA3
PSMD14
IDE
ORC4
ATRIP
Hedgehog signaling pathway
DRG1
RPS27L
LYAR
NFX1
DnaJ molecular chaperone homology domain
GIPC2
LMBR1
RNF32
DNAJB4
RBM33
DNAJC2
DNAJA4
FAM83C
B
GO:0008150
biological process
GO:0009987
cellular process
GO:0065007
biological regulation
GO:0050896
response to stimulus
GO:0051716
cellular response to stimulus
GO:0050789
regulation of biological process
GO:0006950
response to stress
GO:0048583
regulation of response to stimulus
GO:0050794
regulation of cellular process
GO:0033554
cellular response to stress
GO:0080134
regulation of response to stress
GO:0080135
regulation of cellular response to stress
QuickGO - https://www.ebi.ac.uk/QuickGO
Process
Function
Component
Is a
Part of
Regulates
Positively regulates
Negatively regulates
Occurs in
Capable of
Capable of part of

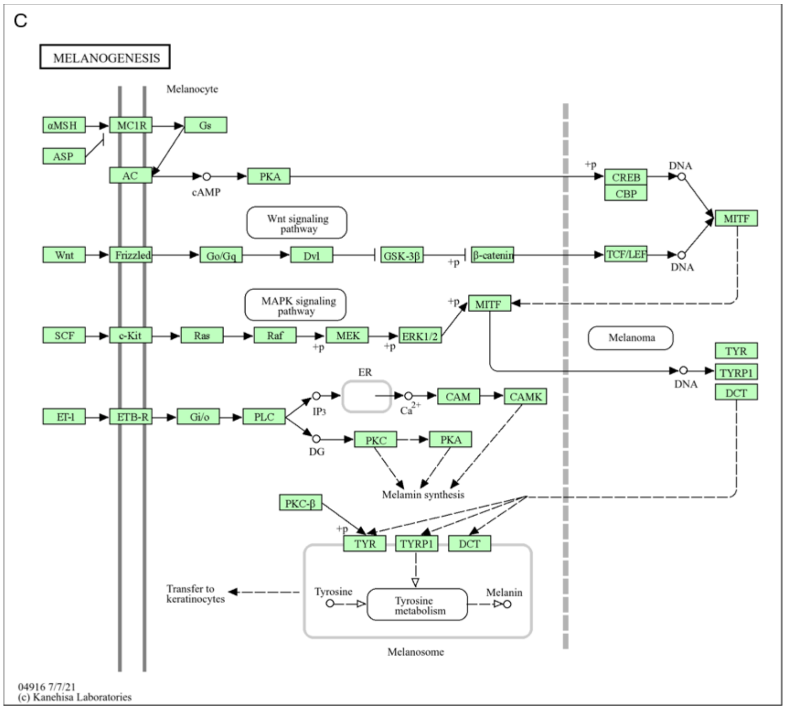

Fig. S27. Interacting Proteins for Admixed individuals with a higher proportion of Brown skuas (Hyb-Br). (A) STRING Interaction Network Preview of enrichment of interactions with clusters linked to the Wnt signaling pathway, Hedgehog signaling pathway (CL:13460), Homeodomain (CL:13739) and interactions among proteins with the DnaJ molecular chaperone homology domain (genes DNAJB4, DNAJC2, DNAJA4, GIPC2), and interaction observed between the Cul3-RING complex and the Hedgehog signaling pathway. Below image about KEGG pathways about melanogenesis (gga04916). (B) Biological process (QuickGO:Term GO:0080135) about any process that modulates the frequency, rate or extent of a cellular response to stress. Cellular response to stress is a change in state or activity of a cell (in terms of movement, secretion, enzyme production, gene expression, etc.) as a result of a stimulus indicating the organism is under stress. The stress is usually, but not necessarily, exogenous (e.g. temperature, humidity, ionizing radiation). (C) KEGG Pathways about interaction between Wnt genes and melagonesis genes (ASP, MC1R, TYR).

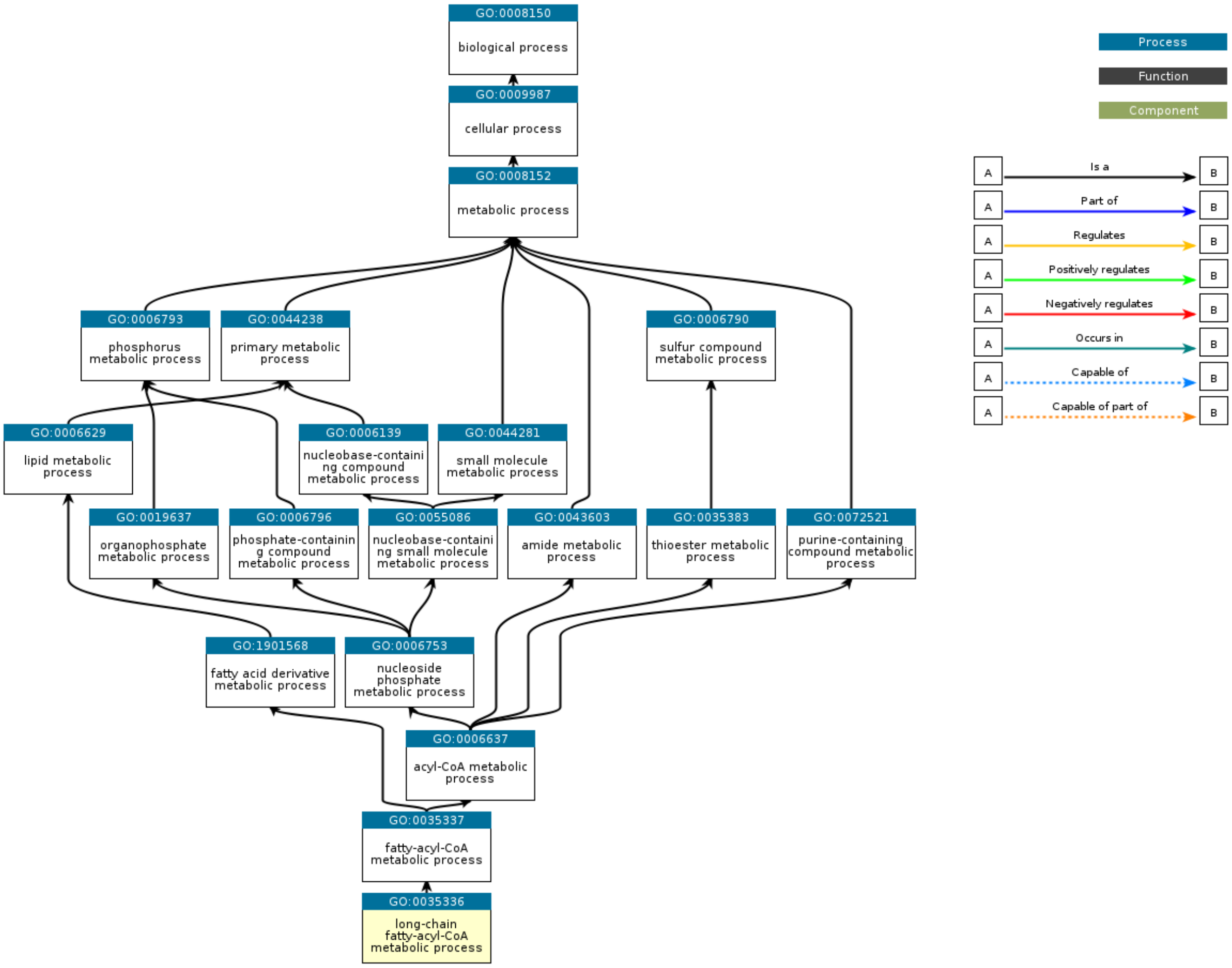

QuickGO - https://www.ebi.ac.uk/QuickGO

Fig. S29. Interacting Proteins for Admixed individuals with a higher proportion of south. polar (Hyb-SP). QuickGO term: GO:0035336, The chemical reactions and pathways involving long-chain fatty-acyl-CoAs, any derivative of coenzyme A in which the sulfhydryl group is in a thioester linkage with a long-chain fatty-acyl group.

A

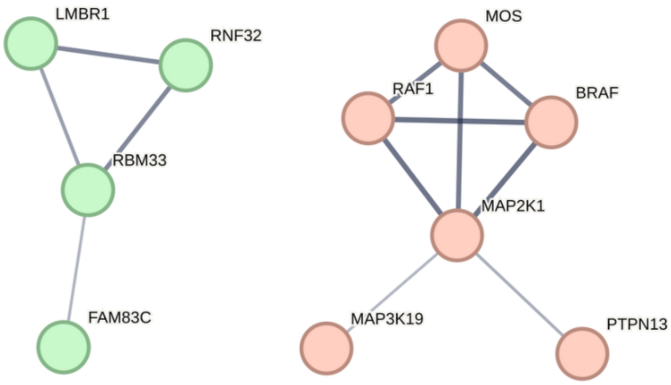

Network STRING. Estrogen-dependent gene expression, and progesterone-mediated oocyte maturation

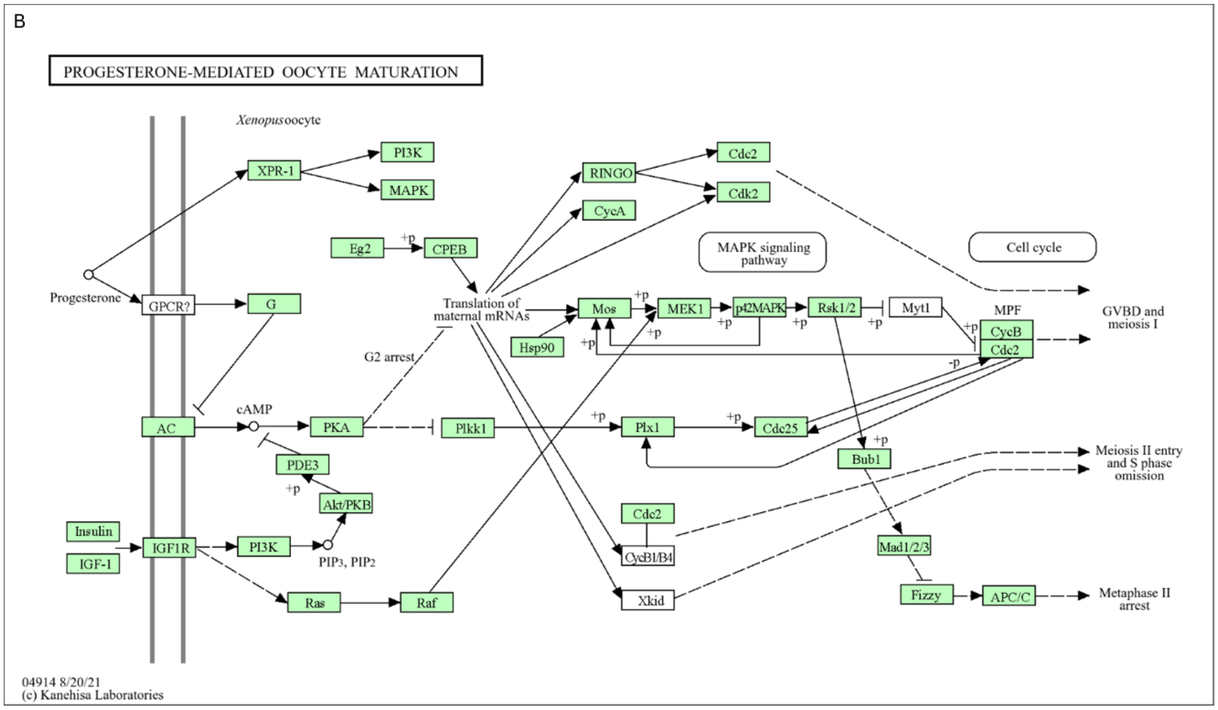

Fig. S29. STRING Interaction Network and KEGG Network Pathways in Hyb-Br. (A) STRING significant interaction of Hyb-Br of estrogen-dependent gene expression, and progesterone-mediated oocyte maturation. (B) KEGG Pathways for progesterone-mediated oocyte maturation.

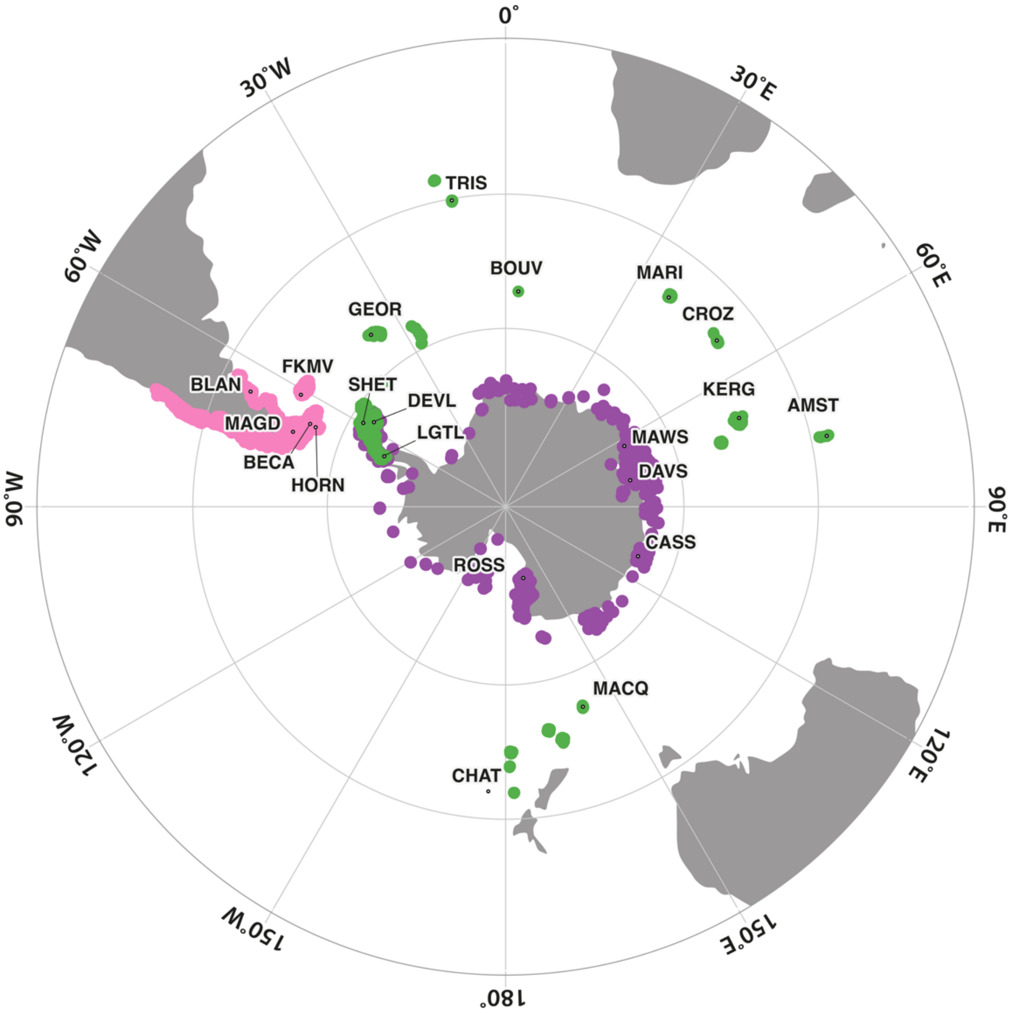

Fig. S30. Map of occurrences for the three species used for Species Distribution models. Each color represents each species, Chilean skua (pink), Brown skua (green) and South Polar (purple).

Table captions

Table S1. List of samples collected from the different species/subspecies and locality with respective acronym, sample size (n).

Table S2. List of all samples from this study with their respective biosample accession ID, locations, geographic position, sample type, date of collection, and stage of life cycle.

Table S3. The number of reads, average depth mapped against *Alca torda*, and against *Rissa tridactyla*, relative depth of Z, sex ID.

Table S4. SNPs datsets with *Alca torda* assembling and *Rissa tridactyla* assembling Including for each dataset the number of species, locations, number of chromosomes, outgroup, total number of SNPs, filters used for each dataset and its respective number of SNPs after each filter and the methodology used.

Table S5. ADMIXTURE results of K=3 with the percentage of the genome belonging to each of the three species for the hybrids individuals.

Table S6. The relationship statistics *D* and f_4 represent relationships between the applicable biallelic SNPs in four populations: P1, P2, P3, and O. The site patterns are ordered such that the pattern BBAA refers to P1 and P2 that share the derived allele, ABBA to P2 and P3 that share the derived allele, and BABA to P1 and P3 that share the derived allele. Under the null hypothesis, which assumes no gene flow, the ABBA and BABA patterns are expected to occur due to incomplete lineage sorting with equal frequencies, and a significant deviation from that expectation is consistent with introgression between P3 and P1 or P2.

Table S7. Results of the F-branch program. A matrix with f-branch statistics is generated for each branch of the tree, including internal branches, reflecting an excess allele shared with each P3 population.

Table S8. The number of genes under positive selection using RAiSD for different datasets (each of the three species, admixed indviduals, and each of the genetic group), and XP-NSL for each species and admixed individuals.

Table S9. List of genes obtained RAiSD for different datasets (each of the three species, admixed indviduals, and each of the genetic group), and XP-NSL for each species and admixed individuals.

Table S10. Enrichment genes obtained from RAiSD common for all three species. The genes are enriched with their respective category, GO biological process, fold enrichment, P-value, FDR, and GO ID number.

Table S11. The genes under selection obtained from RAiSD enriched with their respective category using metascape for Brown skuas.

Table S12. The genes under selection detected for brown skuas using the two methods, RAISD and XP-NSL, and enriched with their respective category using STRING.

Table S13. The genes under selection using RAISD exclusive for Brown skuas (not under selection in the other species) enriched with their respective category using String.

Table S14. The genes under selection obtained from RAiSD enriched with their respective category using metascape for **South Polar skuas**.

Table S15. The genes under selection detected for **South Polar skuas** using the two methods, RAISD and XP-NSL, and enriched with their respective category using Metascape.

Table S16. The genes under selection using RAISD exclusive for **South Polar skua** (not under selection in the other species) enriched with their respective category using String.

Table S17. The genes under selection obtained from RAiSD enriched with their respective category using metascape for **Chilean skuas**.

Table S18. The genes under selection detected for **Chilean skuas** using the two methods, RAISD and XP-NSL, and enriched with their respective category using STRING.

Table S19. The genes under selection using RAISD exclusive for **Chilean skuas** (not under selection in the other species) enriched with their respective category using String.

Table S20. The genes under selection obtained from RAiSD enriched with their respective category using metascape for admixed individuals with a higher proportion of brown skuas **(Hyb-BR)**.

Table S21. The genes under selection detected for **Hyb-BR** using the two methods, RAISD and XP-NSL, and enriched with their respective category using Metascape.

Table S22. The genes under selection using RAISD exclusive for **Hyb-BR** (not under selection in the other species) enriched with their respective category using String.

Table S23. The genes under selection obtained from RAiSD enriched with their respective category using metascape for **Hyb-SP**.

Table S24. The genes under selection detected for admixed individuals with a higher proportion of south polar skuas (**Hyb-SP**) using the two methods, RAISD and XP-NSL, and enriched with their respective category using STRING.

Table S25. The genes under selection using RAISD exclusive for **Hyb-SP** (not under selection in the other species) enriched with their respective category using String.

Table S26- ENM measures of performance: Training AUC, Test AUC, AUC Standard Deviation, Training omission rate, Test omission rate, and Prevalence for each of the six models for each species and the two types of hybrids.

Table S27- Summary information for the 7 populations (3 skua species and 4 hybridization statuses) x 4 climate change scenarios (present to 2025-RCP2.6, 2050-RCP8.5, 2100-RCP2.6 and

to 2100-RCP8.5) range shift changes with the percentage of niche gain, loss and stability, as well as the current and future range size future range size, and ultimately the net species range change (gains minus losses).

Table S28-List of software and algorithms used with its respective software version, reference and GitHub or URL.

References

Abbott R, Albach D, Ansell S, Arntzen JW, Baird SJE, Bierne N, Boughman J, Brelsford A, Buerkle CA, Buggs R, et al. 2013. Hybridization and speciation. *J Evol Biol.* 26:229–246.

Alachiotis N, Pavlidis P. 2018. RAiSD detects positive selection based on multiple signatures of a selective sweep and SNP vectors. *Commun Biol.* 1:79.

Alexander DH, Novembre J, Lange K. 2009. Fast model-based estimation of ancestry in unrelated individuals. *Genome Res.* 19:1655–1664.

Aljanabi SM, Martinez I. 1997. Universal and rapid salt-extraction of high-quality genomic DNA for PCR-based techniques. *Nucleic Acids Res.* 25:4692–4693.

Andersson M. 1999. Hybridization and skua phylogeny. *Proc R Soc Lond B Biol Sci.* 266:1579–1585.

Andrews S. 2010. FastQC: a quality control tool for high throughput sequence data. Available from: https://www.bioinformatics.babraham.ac.uk/projects/fastqc/, last accessed November 22, 2024.

Assis J, Tyberghein L, Bosch S, Verbruggen H, Serrão EA, De Clerck O. 2018. Bio-ORACLE v2.0: Extending marine data layers for bioclimatic modelling. *Glob Ecol Biogeogr.* 27:277–284.

Bald L, Gottwald J, Zeuss D. 2023. spatialMaxent: Adapting species distribution modeling to spatial data. *Ecol Evol.* 13:e10635.

Barnes-Vélez JA, Aksoy Yasar FB, Hu J. 2022. Myelin lipid metabolism and its role in myelination and myelin maintenance. *Innovation (Camb).* 4:100360.

Barnett RL, Austermann J, Dyer B, Telfer MW, Barlow NLM, Boulton SJ, Carr AS, Creel RC. 2023. Constraining the contribution of the Antarctic Ice Sheet to Last Interglacial sea level. *Sci Adv.* 9:eadf0198.

Blanco-Pastor JL. 2022. Alternative modes of introgression-mediated selection shaped crop adaptation to novel climates. *Genome Biol Evol.* 14:evac107.

Bornelöv S, Seroussi E, Yosefi S, Benjamini S, Miyara S, Ruzal M, Grabherr M, Rafati N, Molin AM, Pendavis K, et al. 2018. Comparative omics and feeding manipulations in chicken indicate a shift of the endocrine role of visceral fat towards reproduction. *BMC Genomics.* 19:295.

Campagna L, Toews DP. 2022. The genomics of adaptation in birds. *Curr Biol.* 32:R1173–R1186.

Carneiro APB, Polito MJ, Sander M, Trivelpiece WZ. 2010. Abundance and spatial distribution of sympatrically breeding *Catharacta* spp. (Skuas) in Admiralty Bay, King George Island, Antarctica. *Polar Biol.* 33:673–682.

Černý D, Natale R. 2022. Comprehensive taxon sampling and vetted fossils help clarify the time tree of shorebirds (Aves, Charadriiformes). *Mol Phylogenet Evol.* 177:107620.

Chen S, Zhou Y, Chen Y, Gu J. 2018. fastp: An ultra-fast all-in-one FASTQ preprocessor. *Bioinformatics.* 34:i884–i890.

Choi JY, Dai X, Alam O, Peng JZ, Rughani P, Hickey S, Harrington E, Juul S, Ayroles JF, Purugganan MD, et al. 2021. Ancestral polymorphisms shape the adaptive radiation of *Metrosideros* across the Hawaiian Islands. *Proc Natl Acad Sci USA.* 118:e2023801118.
Chondronikola M, Volpi E, Børsheim E, Porter C, Annamalai P, Enerbäck S, Lidell ME, Saraf MK, Labbe SM, Hurren NM, et al. 2014. Brown adipose tissue improves whole-body glucose homeostasis and insulin sensitivity in humans. *Diabetes.* 63:4089–4099.
Chu PC, Eisenschenk S, Zhu ST. 2009. Skeletal morphology and the phylogeny of skuas (Aves: Charadriiformes, Stercorariidae). *Zool J Linn Soc.* 157:612–621.
Cimino MA, Lynch HJ, Saba VS, Oliver MJ. 2016. Projected asymmetric response of Adélie penguins to Antarctic climate change. *Sci Rep.* 6:28785.
Cingolani P, Platts A, Wang LL, Coon M, Nguyen T, Wang L, Land SJ, Lu X, Ruden DM. 2012. A program for annotating and predicting the effects of single nucleotide polymorphisms, SnpEff: SNPs in the genome of *Drosophila melanogaster* strain w1118; iso-2; iso-3. *Fly (Austin).* 6:80–92.
Civel-Mazens M, Crosta X, Cortese G, Michel E, Mazaud A, Ther O, Ikehara M, Itaki T. 2021. Antarctic Polar Front migrations in the Kerguelen Plateau region, Southern Ocean, over the past 360 kyrs. *Glob Planet Change.* 202:103526.
Cohen BL, Baker AJ, Blechschmidt K, Dittmann DL, Furness HD, Gerwin JA, Helbig AJ, De Korte J, Marshall HD, Palma RL, et al. 1997. Enigmatic phylogeny of skuas (Aves: Stercorariidae). *Proc R Soc Lond B Biol Sci.* 264:181–190.
Costa ES, Alves MAS. 2012. Climatic changes, glacial retraction and the skuas (*Catharacta* sp.– Stercorariidae) in Hennequin Point (King George Island, Antarctic Peninsula). *Pesq Antart Bras.* 5:163–170.
Daković N, Térézol M, Pitel F, Maillard V, Elis S, Leroux S, Lagarrigue S, Gondret F, Klopp C, Baeza E, et al. 2014. The loss of adipokine genes in the chicken genome and implications for insulin metabolism. *Mol Biol Evol.* 31:2637–2646.
Danecek P, Bonfield JK, Liddle J, Marshall J, Ohan V, Pollard MO, Whitwham A, Keane T, McCarthy SA, Davies RM. 2021. Twelve years of SAMtools and BCFtools. *Gigascience.* 10:giab008.
Desktop EA. 2020. Release 10.8.1. Environmental Systems Research Institute: Redlands, CA, USA. (Software and reports are not formatted as journal articles but retain original citation style.)
Devillers P. 1977. The Skuas of the North American Pacific Coast. *Auk.* 94:417–429.
Devillers P. 1978. Distribution and relationships of South American skuas. *Gerfaut.* 68:374–417.
Ding Q, Hu Y, Xu S, Wang J, Jin L. 2014. Neanderthal introgression at chromosome 3p21.31 was under positive natural selection in East Asians. *Mol Biol Evol.* 31:683–695.
Durand EY, Patterson N, Reich D, Slatkin M. 2011. Testing for ancient admixture between closely related populations. *Mol Biol Evol.* 28:2239–2252.
Edelman NB, Frandsen PB, Miyagi M, Clavijo B, Davey J, Dikow RB, García-Accinelli G, Van Belleghem SM, Patterson N, Neafsey DE, et al. 2019. Genomic architecture and introgression shape a butterfly radiation. *Science.* 366:594–599.
Egan SP, Ragland GJ, Assour L, Powell THQ, Hood GR, Emrich S, Nosil P, Feder JL. 2015. Experimental evidence of genome-wide impact of ecological selection during early stages of speciation-with-gene-flow. *Ecol Lett.* 18:817–825.
Ewels P, Magnusson M, Lundin S, Käller M. 2016. MultiQC: summarize analysis results for multiple tools and samples in a single report. *Bioinformatics.* 32:3047–3050.
Felsenstein J. 1993. PHYLIP (phylogeny inference package), version 3.5c. Joseph Felsenstein. (Software and reports are cited without journal formatting.)

Feng Q, Lu D, Xu S. 2018. AncestryPainter: A graphic program for displaying ancestry composition of populations and individuals. *Genomics Proteomics Bioinformatics.* 16:382–385.
Fitak RR. 2021. OptM: Estimating the optimal number of migration edges on population trees using Treemix. *Biol Methods Protoc.* 6:bpab017.
Fraser CI, Nikula R, Ruzzante DE, Waters JM. 2012. Poleward bound: Biological impacts of Southern Hemisphere glaciation. *Trends Ecol Evol.* 27:462–471.
Frichot E, François O. 2015. LEA: An R package for landscape and ecological association studies. *Methods Ecol Evol.* 6:925–929.
Frugone MJ, Lowther A, Noll D, Ramos B, Pistorius P, Dantas GPM, Petry MV, Bonadonna F, Steinfurth A, Polanowski A, et al. 2018. Contrasting phylogeographic pattern among *Eudyptes* penguins around the Southern Ocean. *Sci Rep.* 8:1.
Gandini PA, Frere E. 1998. Seabird and shorebird diversity and associated conservation problems in Puerto Deseado, Patagonia, Argentina. *Ornitol Neotrop.* 9:13–22.
GBIF.Org User. 2023. Occurrence Download. The Global Biodiversity Information Facility.
Gómez GA, García J-L, Villagrán C, Lüthgens C, Abarzúa AM. 2022. Vegetation, glacier, and climate changes before the global last glacial maximum in the Isla Grande de Chiloé, southern Chile (42° S). *Quat Sci Rev.* 276:107301.
Griffiths HJ, Barnes DKA, Linse K. 2009. Towards a generalized biogeography of the Southern Ocean benthos. *J Biogeogr.* 36:162–177.
Hamlin JAP, Hibbins MS, Moyle LC. 2020. Assessing biological factors affecting postspeciation introgression. *Evol Lett.* 4:137–154.
Hanssen MJ, Hoeks J, Brans B, van der Lans AA, Schaart G, van den Driessche JJ, Jörgensen JA, Boekschoten MV, Hesselink MK, Havekes B, et al. 2015. Short-term cold acclimation improves insulin sensitivity in patients with type 2 diabetes mellitus. *Nat Med.* 21:863–865.
Hawks J. 2017. Introgression Makes Waves in Inferred Histories of Effective Population Size. *Hum Biol.* 89:67–80.
Hearing VJ. 2000. The melanosome: the perfect model for cellular responses to the environment. *Pigment Cell Res.* 13 Suppl 8:23–34.
Hemmings AD. 1984. Aspects of the breeding biology of McCormick's skua *Catharacta maccormicki* at Signy Island, South Orkney Islands. *Bull Br Antarct Surv.* 65:65–79.
Howarth C, Gleeson P, Attwell D. 2012. Updated Energy Budgets for Neural Computation in the Neocortex and Cerebellum. *J Cereb Blood Flow Metab.* 32:1222–1232.
Jäättelä M. 1999. Heat shock proteins as cellular lifeguards. *Ann Med.* 31:261–271.
Kanehisa M, Goto S. 2000. KEGG: Kyoto Encyclopedia of Genes and Genomes. *Nucleic Acids Res.* 28:27–30.
Karastergiou K, Fried SK, Xie H, Lee MJ, Divoux A, Rosencrantz MA, Chang RJ, Smith SR. 2013. Distinct developmental signatures of human abdominal and gluteal subcutaneous adipose tissue depots. *J Clin Endocrinol Metab.* 98:362–371.
Kersten O, Star B, Krabberød AK, Atmore LM, Tørresen OK, Anker-Nilssen T, Descamps S, Strøm H, Johansson US, Sweet PR, et al. 2023. Hybridization of Atlantic puffins in the Arctic coincides with 20th-century climate change. *Sci Adv.* 9:eadh1407.
Kim SH, Lee SJ, Jo E, Kim J, Kim JU, Kim JH, Park H, Chi YM. 2021. Genome of the Southern Giant Petrel Assembled Using Third-Generation DNA Sequencing and Linked Reads Reveals Evolutionary Traits of Southern Avian. *Animals.* 11:Article 7.
Kozlov AM, Darriba D, Flouri T, Morel B, Stamatakis A. 2019. RAxML-NG: A fast, scalable and user-friendly tool for maximum likelihood phylogenetic inference. *Bioinformatics.* 35:4453–4455.

Lehtonen J, Lanfear R. 2014. Generation time, life history and the substitution rate of neutral mutations. *Biol Lett.* 10:20140801.
Li H, Ma Z, Jia L, Li Y, Xu C, Wang T, Han R, Jiang R, Li Z, Sun G, et al. 2016. Systematic analysis of the regulatory functions of microRNAs in chicken hepatic lipid metabolism. *Sci Rep.* 6:31766.
Liang X, Pan J, Cao C, Zhang L, Zhao Y, Fan Y, Li K, Tao C, Wang Y. 2019. Transcriptional Response of Subcutaneous White Adipose Tissue to Acute Cold Exposure in Mice. *Int J Mol Sci.* 20:Article 16.
Liu J, Curry JA, Rossow WB, Key JR, Wang X. 2005. Comparison of surface radiative flux data sets over the Arctic Ocean. *J Geophys Res Oceans.* 110:C2.
Lois NA, Campagna L, Balza U, Polito MJ, Pütz K, Vianna JA, Morgenthaler A, Frere E, Sáenz-Samaniego R, Raya Rey A, et al. 2020. Metapopulation dynamics and foraging plasticity in a highly vagile seabird, the southern rockhopper penguin. *Ecol Evol.* 10:3346–3355.
Malinsky M, Matschiner M, Svardal H. 2021. Dsuite-Fast D-statistics and related admixture evidence from VCF files. *Mol Ecol Resour.* 21:584–595.
Mardones M, González L, King R, Campos E. 2011. Variaciones glaciales durante el Holoceno en Patagonia Central, Aisén, Chile: Evidencias geomorfológicas. *Andean Geol.* 38:371–392.
Masello JF, Quillfeldt P, Sandoval-Castellanos E, Alderman R, Calderón L, Cherel Y, Cole TL, Cuthbert RJ, Marin M, Massaro M, et al. 2019. Additive Traits Lead to Feeding Advantage and Reproductive Isolation, Promoting Homoploid Hybrid Speciation. *Mol Biol Evol.* 36:1671–1685.
Matsuoka K, Skoglund A, Roth G, de Pomereu J, Griffiths H, Headland R, Herried B, Katsumata K, Le Brocq A, Licht K, et al. 2021. Quantarctica, an integrated mapping environment for Antarctica, the Southern Ocean, and sub-Antarctic islands. *Environ Model Softw.* 140:105015.
McDowall RM. 2005. Falkland Islands biogeography: Converging trajectories in the South Atlantic Ocean. *J Biogeogr.* 32:49–62.
McKenna A, Hanna M, Banks E, Sivachenko A, Cibulskis K, Kernytsky A, Garimella K, Altshuler D, Gabriel S, Daly M. 2010. The Genome Analysis Toolkit: A MapReduce framework for analyzing next-generation DNA sequencing data. *Genome Res.* 20:1297–1303.
Meier JI, Marques DA, Mwaiko S, Wagner CE, Excoffier L, Seehausen O. 2017. Ancient hybridization fuels rapid cichlid fish adaptive radiations. *Nat Commun.* 8:14363.
Mi H, Muruganujan A, Casagrande JT, Thomas PD. 2013. Large-scale gene function analysis with the PANTHER classification system. *Nat Protoc.* 8:Article 8.
Mikkelsen EK, Irwin D. 2021. Ongoing production of low-fitness hybrids limits range overlap between divergent cryptic species. *Mol Ecol.* 30:4090–4102.
Mikkelsen EK, Weir JT. 2023. Phylogenomics Reveals that Mitochondrial Capture and Nuclear Introgression Characterize Skua Species Proposed to be of Hybrid Origin. *Syst Biol.* 72:78–91.
Milanesi M, Capomaccio S, Vajana E, Bomba L, Garcia JF, Ajmone-Marsan P, Colli L. 2017. BITE: An R package for biodiversity analyses. *bioRxiv.* Preprint:181610.
Min H, Yang YY, Yang Y. 2024. Cold induces brain region-selective neuronal activity-dependent lipid metabolism. *eLife.* 13:e98353.
Mota ACM, Costa ES, Torres JPM, de Araujo J, Tormena LC, Pires de Mendonça Dantas G. 2023. Brown Skua and south polar Skua (*Aves*: Stercorariidae) a hybridization case or same species? *Polar Biol.* 46:1191–1201.

Mota-Rojas D, Titto CG, Orihuela A, Martínez-Burnes J, Gómez-Prado J, Torres-Bernal F, Flores-Padilla K, Carvajal-de la Fuente V, Wang D. 2021. Physiological and Behavioral Mechanisms of Thermoregulation in Mammals. *Animals (Basel).* 11:1733.

Mund MJ, Miller GD. 1995. Diet of the south polar skua *Catharacta maccormicki* at Cape Bird, Ross Island, Antarctica. *Polar Biol.* 15:453–455.

Nadachowska-Brzyska K, Burri R, Smeds L, Ellegren H. 2016. PSMC analysis of effective population sizes in molecular ecology and its application to black-and-white *Ficedula* flycatchers. *Mol Ecol.* 25:1058–1072.

Okonechnikov K, Conesa A, García-Alcalde F. 2016. Qualimap 2: Advanced multi-sample quality control for high-throughput sequencing data. *Bioinformatics.* 32:292–294.

Orsi AH, Whitworth T, Nowlin WD. 1995. On the meridional extent and fronts of the Antarctic Circumpolar Current. *Deep Sea Res Part I Oceanogr Res Pap.* 42:641–673.

Parmelee DF. 1988. The hybrid skua: A southern ocean enigma. *Wilson Bull.* 100:345–356.

Pertierra LR, Segovia NI, Noll D, Martinez PA, Pliscoff P, Barbosa A, Aragón P, Raya Rey A, Pistorius P, Trathan P, et al. 2020. Cryptic speciation in gentoo penguins is driven by geographic isolation and regional marine conditions: Unforeseen vulnerabilities to global change. *Divers Distrib.* 26:958–975.

Pickrell J, Pritchard J. 2012. Inference of population splits and mixtures from genome-wide allele frequency data. *Nat Preced.* 1–1.

Pietz PJ. 1987. Feeding and Nesting Ecology of Sympatric South Polar and Brown Skuas. *Auk.* 104:617–627.

Purcell S, Neale B, Todd-Brown K, Thomas L, Ferreira MA, Bender D, Maller J, Sklar P, De Bakker PI, Daly MJ. 2007. PLINK: a tool set for whole-genome association and population-based linkage analyses. *Am J Hum Genet.* 81:559–575.

Quilodrán CS, Montoya-Burgos JI, Currat M. 2020. Harmonizing hybridization dissonance in conservation. *Commun Biol.* 3:Article 1.

Quinlan AR, Hall IM. 2010. BEDTools: A flexible suite of utilities for comparing genomic features. *Bioinformatics.* 26:841–842.

Reinhardt K, Blechschmidt K, Peter H-U, Montalti D. 1997. A hitherto unknown hybridization between Chilean and South Polar skua. *Polar Biol.* 17:114–118.

Ritz MS, Hahn S, Janicke T, Peter H-U. 2006. Hybridisation between South polar skua (*Catharacta maccormicki*) and Brown skua (*C. antarctica lonnbergi*) in the Antarctic Peninsula region. *Polar Biol.* 29:153–159.

Ritz MS, Millar C, Miller GD, Phillips RA, Ryan P, Sternkopf V, Liebers-Helbig D, Peter H-U. 2008. Phylogeography of the southern skua complex—Rapid colonization of the southern hemisphere during a glacial period and reticulate evolution. *Mol Phylogenet Evol.* 49:292–303.

Schmickl R, Marburger S, Bray S, Yant L. 2017. Hybrids and horizontal transfer: Introgression allows adaptive allele discovery. *J Exp Bot.* 68:5453–5470.

Schmidt AE, Lescroël A, Lisovski S, Elrod M, Jongsomjit D, Dugger KM, Ballard G. 2023. Sea ice concentration decline in an important Adélie penguin molt area. *Proc Natl Acad Sci USA.* 120:e2306840120.

Schumer M, Xu C, Powell DL, Durvasula A, Skov L, Holland C, Blazier JC, Sankararaman S, Andolfatto P, Rosenthal GG, et al. 2018. Natural selection interacts with recombination to shape the evolution of hybrid genomes. *Science.* 360:656–660.

Shen W, Le S, Li Y, Hu F. 2016. SeqKit: A Cross-Platform and Ultrafast Toolkit for FASTA/Q File Manipulation. *PLoS One.* 11:e0163962.

Singhal S, Derryberry GE, Bravo GA, Derryberry EP, Brumfield RT, Harvey MG. 2021. The dynamics of introgression across an avian radiation. *Evol Lett.* 5:568–581.

Solís-Lemus C, Ané C. 2016. Inferring Phylogenetic Networks with Maximum Pseudolikelihood under Incomplete Lineage Sorting. *PLoS Genet.* 12:e1005896.

Sonna LA, Fujita J, Gaffin SL, Lilly CM. 2002. Invited review: Effects of heat and cold stress on mammalian gene expression. *J Appl Physiol.* 92:1725–1742.

Sozzoni M, Ferrer Obiol J, Formenti G, Tigano A, Paris JR, Balacco JR, Jain N, Tilley T, Collins J, Sims Y. 2023. A chromosome-level reference genome for the black-legged kittiwake (*Rissa tridactyla*), a declining circumpolar seabird. *Genome Biol Evol.* 15:evad153.

Stauch G, Lehmkuhl F. 2010. Quaternary glaciations in the Verkhoyansk Mountains, northeast Siberia. *Quat Res.* 74:145–155.

Strycker N, Wethington M, Borowicz A, Forrest S, Witharana C, Hart T, Lynch HJ. 2020. A global population assessment of the Chinstrap penguin (*Pygoscelis antarctica*). *Sci Rep.* 10:Article 1.

Sun H, Jiang R, Xu S, Zhang Z, Xu G, Zheng J, Qu L. 2015. Transcriptome responses to heat stress in hypothalamus of a meat-type chicken. *J Anim Sci Biotechnol.* 6:6.

Szklarczyk D, Kirsch R, Koutrouli M, Nastou K, Mehryary F, Hachilif R, Gable AL, Fang T, Doncheva NT, Pyysalo S, et al. 2023. The STRING database in 2023: Protein–protein association networks and functional enrichment analyses for any sequenced genome of interest. *Nucleic Acids Res.* 51:D638–D646.

Szpiech ZA, Hernandez RD. 2014. selscan: an efficient multithreaded program to perform EHH-based scans for positive selection. *Mol Biol Evol.* 31:2824–2827.

Tao C, Huang S, Wang Y, Wei G, Zhang Y, Qi D, Wang Y, Li K. 2015. Changes in white and brown adipose tissue microRNA expression in cold-induced mice. *Biochem Biophys Res Commun.* 463:193–199.

Taylor HS. 2000. The role of HOX genes in the development and function of the female reproductive tract. *Semin Reprod Med.* 18:81–89.

Taylor SA, Larson EL. 2019. Insights from genomes into the evolutionary importance and prevalence of hybridization in nature. *Nat Ecol Evol.* 3:Article 2.

Techow NMSM, O'Ryan C, Phillips RA, Gales R, Marin M, Patterson-Fraser D, Quintana F, Ritz MS, Thompson DR, Wanless RM, et al. 2010. Speciation and phylogeography of giant petrels *Macronectes*. *Mol Phylogenet Evol.* 54:472–487.

Thornton K. 2003. The electrophysiological effects of a brain injury on auditory memory functioning. *Arch Clin Neuropsychol.* 18:363–378.

Thuiller W, Georges D, Engler R, Breiner F, Georges MD, Thuiller CW. 2016. Package 'biomod2'. Species Distribution Modeling within an Ensemble Forecasting Framework.

Trivelpiece W, Butler RG, Volkman NJ. 1980. Feeding Territories of Brown Skuas (*Catharacta lonnbergi*). *Auk.* 97:669–676.

Valavi R, Elith J, Lahoz-Monfort JJ, Guillera-Arroita G. 2019. blockCV: An r package for generating spatially or environmentally separated folds for k-fold cross-validation of species distribution models. *Methods Ecol Evol.* 10:225–232.

Vasimuddin M, Misra S, Li H, Aluru S. 2019. Efficient architecture-aware acceleration of BWA-MEM for multicore systems. *Proc IEEE Int Parallel Distrib Process Symp.* 314–324.

Veytia D, Corney S, Meiners KM, Kawaguchi S, Murphy EJ, Bestley S. 2020. Circumpolar projections of Antarctic krill growth potential. *Nat Clim Change.* 10:Article 6.

Vianna JA, Fernandes FAN, Frugone MJ, Figueiró HV, Pertierra LR, Noll D, Bi K, Wang-Claypool CY, Lowther A, Parker P, et al. 2020. Genome-wide analyses reveal drivers of penguin diversification. *Proc Natl Acad Sci USA.* 117:22303–22310.

Weir BS, Cockerham CC. 1984. Estimating F-statistics for the analysis of population structure. *Evolution.* 38:1358–1370.

Wickham H. 2011. *Ggplot2*. *Wiley Interdiscip Rev Comput Stat.* 3:180–185.

Wilmes SB, Ward S, Uehara K. 2023. Present day: Tides in a changing climate. In: Green M, Duarte JC, editors. *A Journey Through Tides*. Elsevier. p. 185–229.
Young EC. 1963. The Breeding Behaviour of the South Polar Skua *Catharacta maccormicki*. *Ibis.* 105:203–233.
Yu Y, Hill AP, McCormick DA. 2012. Warm body temperature facilitates energy efficient cortical action potentials. *PLoS Comput Biol.* 8:e1002456.
Zachary AS, Taylor EN, Novak TE, Bailey NP, Stevison LS. 2021. Application of a novel haplotype-based scan for local adaptation to study high-altitude adaptation in rhesus macaques. *Evol Lett.* 5:408–421.
